# Discovery of New Selective Butyrylcholinesterase (BChE) Inhibitors with Anti-Aβ Aggregation Activity: Structure-Based Virtual Screening, Hit Optimization and Biological Evaluation

**DOI:** 10.3390/molecules24142568

**Published:** 2019-07-15

**Authors:** Cheng-Shi Jiang, Yong-Xi Ge, Zhi-Qiang Cheng, Yin-Yin Wang, Hong-Rui Tao, Kongkai Zhu, Hua Zhang

**Affiliations:** 1 School of Biological Science and Technology, University of Jinan, Jinan 250022, China; 2 Drug Discovery and Design Center, State Key Laboratory of Drug Research, Shanghai Institute of Meteria Medica, Chinese Academy of Sciences, Shanghai 201203, China

**Keywords:** selective BChE inhibitor, virtual screening, structural optimization, molecular dynamics, anti-Aβ aggregation

## Abstract

In this study, a series of selective butyrylcholinesterase (BChE) inhibitors was designed and synthesized from the structural optimization of hit **1**, a 4-((3,4-dihydroisoquinolin-2(1*H*)-yl)methyl)benzoic acid derivative identified by virtual screening our compound library. The in vitro enzyme assay results showed that compounds **9** ((4-((3,4-dihydroisoquinolin-2(1*H*)-yl)methyl)phenyl)(pyrrolidin-1-yl)methanone) and **23** (*N*-(2-bromophenyl)-4-((3,4-dihydroisoquinolin-2(1*H*)-yl)methyl)benzamide) displayed improved BChE inhibitory activity and good selectivity towards BChE versus AChE. Their binding modes were probed by molecular docking and further validated by molecular dynamics simulation. Kinetic analysis together with molecular modeling studies suggested that these derivatives could target both the catalytic active site (CAS) and peripheral anionic site (PAS) of BChE. In addition, the selected compounds **9** and **23** displayed anti-Aβ_1–42_ aggregation activity in a dose-dependent manner, and they did not show obvious cytotoxicity towards SH-SY5Y neuroblastoma cells. Also, both compounds showed significantly protective activity against Aβ_1-42_-induced toxicity in a SH-SY5Y cell model. The present results provided a new valuable chemical template for the development of selective BChE inhibitors.

## 1. Introduction

Alzheimer’s disease (AD), one of the most common neurodegenerative disorders, accounts for about 60–80% of all cases of dementia [1,2]. According to the statistics, nearly 50 million people worldwide have AD or a related dementia, and the number of AD patients is expected to triple by 2050 [3]. AD has now been the third leading cause of death, outpaced only by cardiovascular diseases and cancer [4]. However, there is still no successful therapy or drug to reverse or even slow the course of this disease [5].

Although the pathogenesis of AD is complex and not fully understood, several important clinical hallmarks, such as low level of acetylcholine (ACh), beta-amyloid (Aβ) protein aggregation, and tau (τ)-protein phosphorylation, are involved in the occurrence and development of AD [6]. In recent years, therapies for anti-AD primarily focused on Aβ and tau have received more attention [7], however, various Aβ- and tau-targeting agents have failed in clinical trials [8]. Based on cholinergic dysfunction hypothesis, increasing the level of ACh in the brain to improve cholinergic neurotransmission is still the most effective therapy for AD treatment [9]. The ACh in the brain can be mainly hydrolyzed two types of cholinesterases (ChEs), namely acetylcholinesterase (AChE) and butyrylcholinesterase (BChE) [10]. In the brain of healthy adults, AChE is responsible for 80% of the ACh activity, nearly 10^13^ fold more active than BChE [11]. Four AChE inhibitors (tacrine, donepezil, galantamine, and rivastigmine) have been clinically employed for the management of AD [12]. These drugs have beneficial effects on improving the cognitive and memorial functions of AD patients at mild and moderate stages, but tacrine was withdrawn from the market in 2013 due to its hepatotoxicity and the others drugs cannot stop or reverse the course of AD. In addition, the side effects (e.g., nausea, vomiting, diarrhea) of AChE inhibitors also limit the administered doses of these drugs [13].

Although BChE played a minor role in regulating the brain ACh level in the health brain, increasing experimental evidence demonstrated that the function of BChE in termination of cholinergic neurotransmission significantly changed in brains of patients with progressive AD. In advanced AD patients, the activity of BChE progressively increases while the AChE activity remains unchanged or declines [14,15,16]. Moreover, BChE was believed to compensate the function of AChE based on the experiment that AChE knockout mice could still survive with the normal level and localization of BChE [17,18], while silent BChE mutations in humans showed a slower rate of cognitive decline [19]. Therefore, inhibition of BChE was also expected to contribute to ameliorating a cholinergic effect. This hypothesis was in agreement with the experiment that selective inhibition of BChE could significantly raise ACh levels and improve the cognitive performance of aged rats, without any adverse parasympathetic side effects, which are known to be the defect of traditional AChE inhibitors [15,20]. Taken together, selective inhibition on BChE could represent a promising clinical treatment strategy for AD.

However, the development of inhibitors selectively targeting BChE over AChE is a challenging task, since the two isoforms hAChE and hBChE are highly homologous proteins [21]. Although many structurally diverse selective BChE inhibitors, such as carbamate-based compounds [22], paltanic acid derivatives [23], naphthamide compounds [24], phenserine-based compounds [25], and isosorbide analogues [26], have been reported, very few of them (bisnorcymserine and eptastigmine) have entered into clinical trials [16,27]. Thus, the discovery of effective selective BChE inhibitors with novel molecular skeletons is urgently needed. Virtual screening has been proven to be a very powerful approach for discovering new drug hits with desired properties and structural diversity [28], while a structure-activity relationship (SAR) study based on enzyme or cell bioassay has emerged as the most versatile to in provide useful information for structural modification and improvement [29,30].

**Figure 1:**
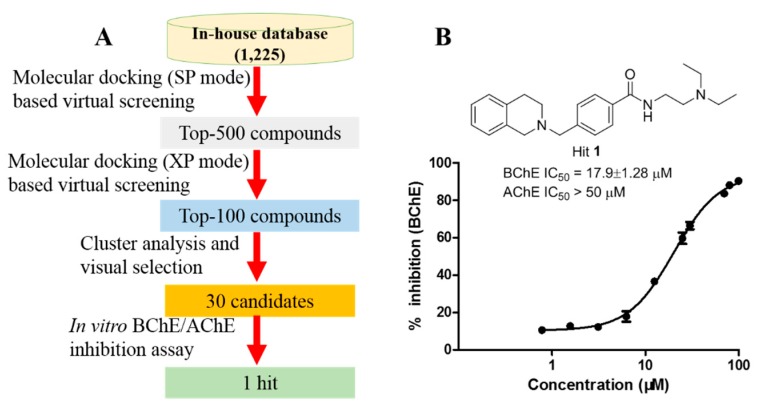
In silico screening procedure and ChE assay results of hit **1**. (**A**) The discovery workflow of the hit compound. (**B**) Chemical structure of hit **1** and its ChE inhibitory activity.

In our project for discovering new ChE inhibitors [31,32,33,34,35,36], a new selective BChE inhibitor **1** (BChE, IC_50_ = 17.94 ± 1.86 μM; AChE, IC_50_ > 50 μM) (Figure 1 and Table 1) was recently identified from our in-house compound library by performing virtual screening and in vitro BChE/AChE assay. The follow-up molecular docking-guided chemical optimization and SAR study starting from hit **1** resulted in some new potent selective BChE inhibitors. Among them, compounds **9** and **23** showed improved BChE inhibitory activity by 9 and 21 folds compared with **1**. In addition, both compounds **9** and **23** displayed anti-Aβ aggregation activity and nontoxicity against SH-SY5Y cells. Herein, we describe a series of new selective BChE inhibitors with anti-Aβ aggregation activity using the combination of virtual screening/molecular, synthesis and in vitro biological evaluation, as well as the molecular dynamics simulation study on the most potent compound **23**.

## 2. Results and Discussion

### 2.1. Molecular Docking-Based Virtual Screening to Identify Hit 1 as Promising Selective BChE Inhibitor

The BChE:tacrine complex crystal structure (PDB entry: 4BDS) [37] was used to perform molecular docking-based virtual screening. Residues in BChE centered on tacrine (15 Å) were defined as a binding pocket. An in-house database, containing 1225 compounds with different scaffold was screened in silico to identify the active compounds against BChE. The docking score of tacrine redocked to BChE was −8.498, while the docking scores of the top-100 compounds was higher than that of tacrine, so the top-100 compounds ranked according to the docking score were obtained for the following visual selection. After clustering the 100 compounds with “Clustering Molecules” protocols inserted in Pipeline Plot 7.5, we finally selected 30 compounds for experimental validation. Among them, compound 1, showed highest inhibitory activity against BChE with IC_50_ value of 17.94 ± 1.86 μM, without obvious activity towards AChE (inhibitory ratio = 26.6% at 50 μM). A literature survey showed that compound **1** was a new compound that was structurally different from the previously reported BChE inhibitor, which highlighted that compound **1** was used as a hit compound for subsequent structural optimization.

### 2.2. Binding mode of 1 Provides Insights for Hit Optimization

**Figure 2:**
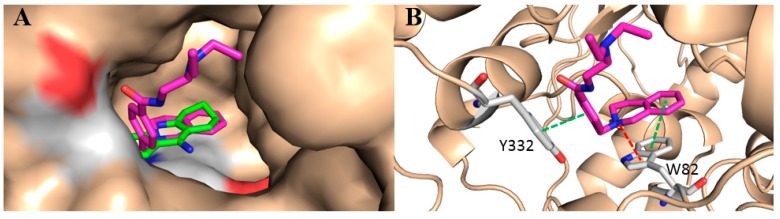
Proposed binding mode of hit **1** with BChE. (**A**) The predicted orientation of **1** (carbon in magenta) in the active pocket of BChE (PDB: 4BDS) with tacrine as ligand in green color; (**B**) The key interactions of **1** with residues in the active sites of BChE. π-π stacking and cation-π interaction are displayed in green and red dashed lines, respectively.

In order to get guidance for rational structural optimization of hit 1, the proposed binding mode of 1 with the active sites of BChE was explored using a molecular docking method. As depicted in Figure 2A, compound **1** fitted well in the active-site gorge of BChE, and its tertrahydroisoquinoline fragment was located in the catalytic active site (CAS) which had spatial overlapping with tacrine, while the amino side-chain was oriented towards the peripheral anionic site (PAS) of BChE. Figure 2B showed the important interactions involved between compound **1** and the residues of BChE, including π-π and cation-π interactions of tertrahydroisoquinoline fragment with W82 (a key residue in the CAS) and π-π interaction of benzene fragment with Y332 (a key residue in the PAS of BChE). Since the interactions with W82 and Y332 play an important role in BChE inhibition, the two key fragments (tertrahydroisoquinoline and benzene) should be retained before performing chemical modifications. With this in mind, the amino side-chain was then rationally modified based on the above molecular docking result in order to improve its inhibitory activity against BChE.

### 2.3. Chemcial Synthesis

**Scheme 1:**
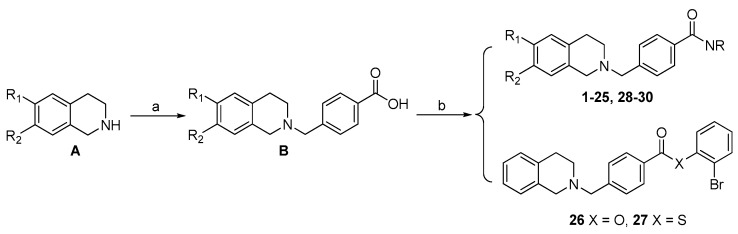
Synthesis of compounds **1**–**30**. (**a**) 4-(bromomethyl)benzoic acid or its derivatives, THF, reflux, 8 h; (**b**) agents including alkyl amines, aromatic amines, phenol, thiophenol or tryptamines, HATU, DIPEA, DMF, r.t., 8 h.

To improve the potency of hit 1 and to discuss the structure-activity relationship (SAR), a range of amide and ester derivatives of 4-((3,4-dihydroisoquinolin-2(1*H*)-yl)methyl)benzoic acid were designed and synthesized based on molecular docking analysis. The synthetic route outlined in Scheme 1 started from the commercial tertrahydroisoquinoline or its derivatives A, which reacted with 4-(bromomethyl)benzoic acid to give intermediates B. Subsequent condensation reaction of acids B with various alkyl amines, aromatic amines, phenol, thiophenol or tryptamines smoothly yielded the target compounds **1**–**30**.

### 2.4. Biological Evaluation and SAR Study

The Ellman’s method was used to determine all the compounds’ BChE and AChE inhibitory activities, and tacrine was used as the reference control. The SAR investigation was mainly focused on the amino side-chain in the first round of structural modification. From Table 1, modification of the terminal amino group in compound **1** using homologue (compounds **2**–**4**) or isostere (compound **5**) led to a significant loss of BChE inhibitory activity or selectivity towards BChE. Scaffold hopping-based cyclization of the diethylamino group was tried to yield analogs 6–10, of which the compounds **7**–**9** possessing tetrahydropyrrole ring showed improved inhibition and selectivity towards BChE, with the best IC_50_ value of 2.68 ± 0.28 μM for 9.

With compound **9** in hand, the substituent effect at C-6 and C-7 of tetrahydroisoquinoline on the BChE inhibitory activity was studied through the evaluation of analogs 11–14. The results in Table 2 indicated that both monosubstitution and disubstitution made the BChE inhibitory activity and selectivity decrease, compared with **9**, suggesting that it might not accommodate the substitution at C-6 and C-7 of a tetrahydroisoquinoline fragment.

Next, the tetrahydroisoquinoline ring was unchanged and the tetrahydropyrrole group was later replaced by various benzene groups. The results were shown in Table 3. Among compounds **15**–**18**, only one compound (**16**) with 2-fluoroanilino group showed BChE activity (IC_50_ = 6.9 μM), indicating that 2-subsititution at benzene ring might be optimal for BChE activity and 3-/4-subsititution was inadvisable. Notably, replacement of 2-F by 2-Br led to an obvious increase of the activity of 23 by about 3- and 8-fold compared with that of **9** and **16**, respectively. Changing the position of Br from C-2 to C-3 (24) or C-4 (25) obviously made its BChE inhibitory activity lose, which was in accordance with the SAR obtained from compounds **16**–**18**. Then, replacement of 2-bromoanilino moitey in compound 23 by 2-bromophenol (26) or 2-bromophenylthio (27) also led to a significant loss of activity, suggesting that the amide group takes an important role in its activity. Recently, Mahdavi et al. reported a group of potent selective tryptamine-derived BChE inhibitors [38], thus the effect of tryptamine fragment fused into our compounds **28**–**30** on BChE activity was evaluated. Unfortunately, all these three target compounds were inactive toward BChE.

### 2.5. Kinetic Study on BChE Inhibition

**Figure 3:**
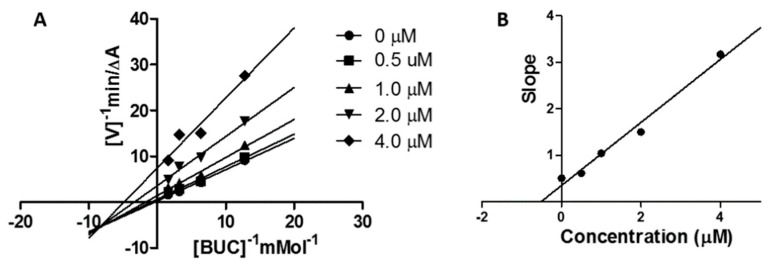
Kinetic study of **23** on BChE inhibition. (**A**) The Lineweaver–Burk reciprocal plots records enzyme velocity curves-substrate concentration in the presence of **23** at different concentrations; (**B**) Plot of slopes versus the concentrations of **23**.

To further understand the action mechanism for BChE inhibition of this series of compounds, the kinetic assay was performed with the optimal compound **23** as a model compound. The enzyme kinetic result reveals that increasing slope (decreasing *V*_max_) and increased *K*_max_ with increasing concentrations of **23** (Figure 3A). This pattern clearly indicates compound **23** is mixed-type inhibitor that simultaneously interacts with both CAS and PAS of BChE. The result was confirmed by the subsequent molecular docking study of **23**. Replots of the slope against the concentrations of **23** indicates that the *K*_I_ value is 0.52 μM, which fits its IC_50_ value for BChE inhibition.

### 2.6. Binding Mode Analysis

**Figure 4:**
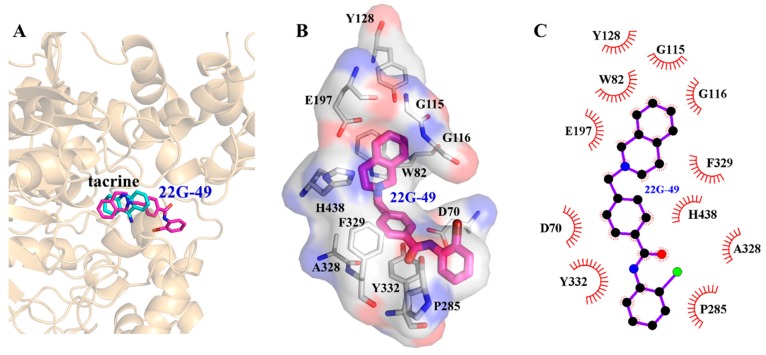
Predicted binding mode of **23** with BChE. (**A**) Superimposition of the binding modes of **23** and tacrine. BChE, **23** and tacrine are shown as light-orange cartoon, magenta, and cyan sticks. (**B**) Detailed interactions between **23** and BChE. Compound **23** is shown as magenta sticks, while the carbon atoms of the interacting residues are colored in white. (**C**) Key interactions involved in stabilizing **23** at the tacrine binding site.

A probable binding mode of **23** with BChE was obtained by conducting molecular docking. As illustrated in Figure 4A, compound **23** could well occupy the tacrine binding site and displayed similar binding mode with tacrine. In addition, the bromophenyl of **23** extended outside of tacrine binding site. As we can see from Figure 4B,C, the binding pocket of **23** was a hydrophobic pocket composed of D70, W82, G115, G116, Y128, E197, P285, A328, F329, Y332, and H438 residues, and the π-π interactions of **23** with W82 and Y332 were retained. Hydrophobic interaction is the main interaction in stabilizing **23** to the tacrine binding site.

### 2.7. Molecular Dynamics

To further validate the binding mode of **23** with BChE, 100 ns molecular dynamics (MD) simulation was employed on the complex model of BChE: **23** that was constructed using molecular docking. The root-mean-square deviations (RMSDs) values for the heavy atoms of BChE and 23 (Figure S1) were steady, which indicated that MD trajectory is reliable for further analysis. Then the binding free energy of **23** with BChE was calculated by Molecular Mechanics Poisson-Boltzmann Surface Area (MM-PBSA) method, as shown in Table 4, the value (−25.93 kcal/mol) indicated a stable binding of **23** with BChE. Besides, based on MD trajectories, the occupancy rate of hydrophobic interactions between **23** and the active site residues was calculated and shown in Table 5. This result indicated that all the hydrophobic interactions between **23** and BChE could be well retained during MD simulation.

### 2.8. Inhibition on Aβ Aggregation

**Figure 5:**
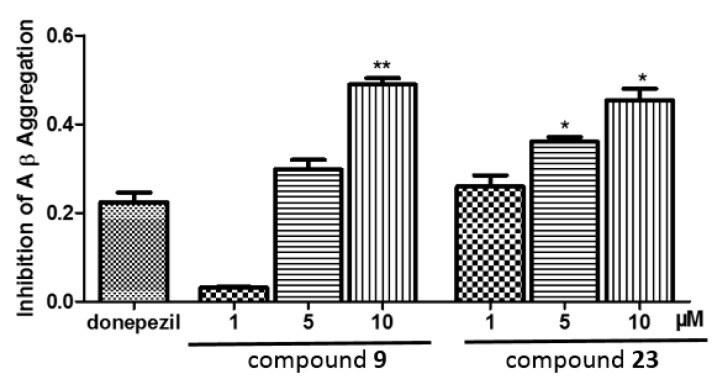
Inhibitory activity against Aβ_1-42_ produced by **9** (1, 5, 10 μM), **23** (1, 5, 10 μM), and donepezil (10 μM). Data are shown as mean ± SD (*n* = 3), with * *p* < 0.05 and ** *p* < 0.01 versus donepezil.

The Aβ peptide aggregation in the brain is known to trigger the onset of neurodegenerative disease and considered as a key pathological hallmark of AD [39,40]. The aggregation and accumulation of the Aβ peptide could form oligomers and plaques, which is a step-by-step process starting from a soluble monomer and proceeding through oligomeric structures, protofibrils, and eventually amyloid fibrils. Of them, the soluble oligomers are the more toxic species [41,42]. BChE was found to be enriched within Aβ plaques in brain of AD patients and to promote the formation of Aβ fibrils, while BChE-knockout could reduce Aβ deposition in brain of AD mouse model [43,44]. Thus, BChE inhibitor could reduce the Aβ aggregation. Based on these observations, inhibitors 9 and **23** were evaluated for their in vitro ability to inhibit Aβ aggregation using the thioflavin T (ThT) method. Donepezil was used as a positive control, and the results was shown in Figure 5. Both compounds exhibited anti-Aβ_1-42_ aggregation activity in a dose-dependent manner from the concentration of **1** to **10** μM. Especially, the inhibition ratios of **9** and **23** (at 10 μM) towards Aβ_1-42_ aggregation are 45.5 ± 6.2% and 44.2 ± 3.3%, respectively, indicating that the activity of both compounds are significantly more potent than that of donepezil (25.8 ± 6.2% at 10 μM).

### 2.9. Cell Viability Assay

**Figure 6:**
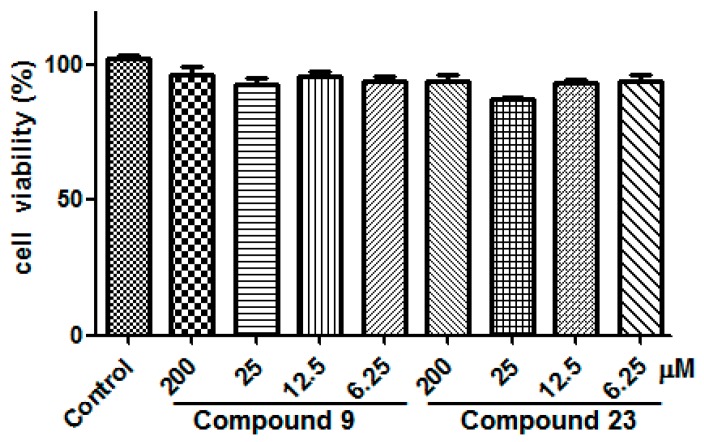
The cytotoxicity of **9** and **23** against SH-SY5Y cells. Date are expressed as the percent variation with respect to the viability recorded in control cells.

Next, to determine the potential cytotoxic activity of selected compounds **9** and **23** towards SH-SY5Y neuroblastoma cells, the viability of cells treated by **9** and **23** was evaluated by MTT assay. The result presented in Figure 6 revealed that both compounds did not show obvious cytotoxicity in the range of tested concentration, even at 200 μM.

### 2.10. Protective Activity of 9 and 23 Against Aβ_1-42_-Induced Toxicity in SH-SY5Y Cells

**Figure 7:**
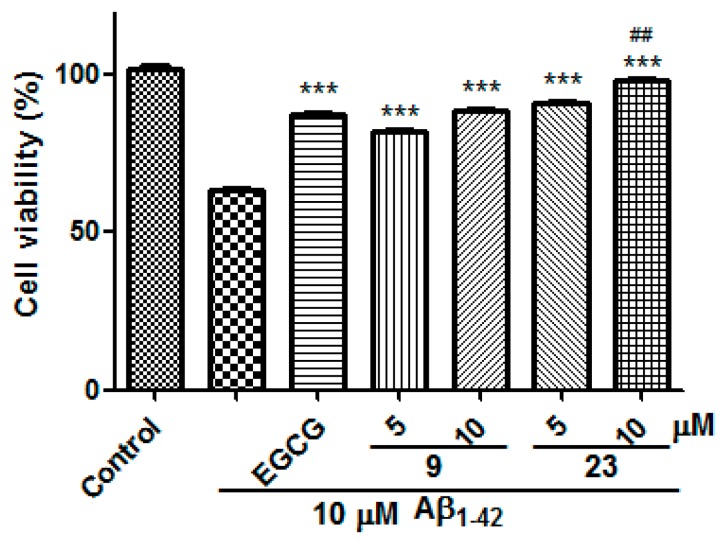
Protective effects of compounds **9** and **23** on Aβ_1-42_-induced toxicity in SH-SY5Y cells. Data are expressed as mean ± SD (*n* = 3), *** *p* < 0.001 versus Aβ_1–42_ treated only, and ^##^ *p* < 0.01 versus positive control EGCG.

Since compounds **9** and **23** showed anti-Aβ_1-42_ aggregation activity and nontoxicity towards SH-SY5Y cells, their in vitro ability to protect SH-SY5Y cells from Aβ_1-42_-induced toxicity was evaluated using the MTT method with epigallocatechin gallate (EGCG) as a positive control. As shown in Figure 7, after the SH-SY5Y cells was exposed to 10 μM Aβ_1-42_ for 24h, the cell viability of Aβ_1-42_-treated group decreased to 63.21 ± 1.30%, while in compound-treated groups both **9** and **23** could significantly protect SH-SY5Y cells from Aβ_1-42_-induced toxicity compared with the Aβ-injured group, by increasing cell viability to 84.74 ± 1.77% and 91.14 ± 1.25 at 5 μM, and 88.80 ± 0.81% and 98.04 ± 1.70 at 10 μM, respectively. More importantly, the protective activity of compound **23** was found to be significantly better than that of the positive control EGCG (87.18 ± 1.29%) at 10 μM. Based on this observation, compound **23** might be a more promising protective agent against Aβ_1-42_-induced toxicity on the SH-SY5Y cell model.

## 3. Materials and Methods

### 3.1. Virtural Screening

An in-house database containing 1225 compounds was screened in silico against the BChE protein structure (PDB code: 4BDS) [37] through the GLIDE 5.5 software [45,46]. The BChE protein coordinates were first processed with the Protein Preparation Wizard Workflow inserted in Maestro, and the default settings were used for this step. Then the docking grids were created by defining residues within 15 Å centered on tacrine in BChE. The parameters for the cutoff, neutralization, scaling, etc. were adopted with default settings. All the in-house database compounds were docked into the defined binding pocket and were ranked by G-score. The top 500 compounds were selected for further molecular docking validation with the extra precision (XP) mode to further enrich the potential active compounds [47]. Finally, after clustering, visual selection was made to yield the 30 candidate compounds, which were subjected to the BChE inhibition assay.

### 3.2. Chemistry

#### 3.2.1. General Methods

Commercially available reagents were used without further purification. Organic solvents were evaporated with reduced pressure using a Büchi R-100 evaporator (Büchi, Flawil, Switzerland). Reactions were monitored by TLC using Yantai Jingyou (Yantai, China) GF254 silica gel plates. Silica gel column chromatography was performed on Biotage Isolera One (Biotage, Uppsala, Sweden) using silica gel (200–300 mesh) from Qingdao Hailang (Qingdao, China). NMR spectra were measured on Bruker Avance III 600 MHz spectrometers (Bruker, Fällanden, Switzerland). Chemical shifts were expressed in *δ* (ppm) and coupling constants (*J*) in Hz with solvent signals as internal standards (CDCl_3_, *δ*_H_ 7.26 ppm and *δ*_C_ 77.0 ppm; DMSO-*d*_6_, *δ*_H_ 2.50 ppm and *δ*_C_ 39.5 ppm). ESI-MS (electrospray ionization mass spectrometry) analyses were performed on an Agilent 1260–6460 Triple Quard LC-MS instrument (Agilent, Waldbronn, Germany), and HR-ESIMS data were acquired on an Agilent Q-TOF 6520 (Agilent, Waldbronn, Germany).

#### 3.2.2. General Procedure for the Synthesis of B

The mixtures of 4-(bromomethyl)benzoic acid (200 mg, 0.93 mmol, 1 equiv.) and corresponding tetrahydroisoquinolines **A** (1.86 mmol, 2 equiv.) in 8 mL anhydrous THF were refluxed for 8 h, and the progress of the reaction was followed using TLC. After completion of the reaction, the reaction mixture was cooled to room temperature and the solvent was removed under vacuo. The resultant solid was dissolved in 1N NaOH (20 mL) and then extracted with CH_2_Cl_2_ (3 × 20 mL). The aqueous layer was acidified to pH = 1 using 10% HCl (20 mL), resulting in precipitation of the final compounds.

*4-((3,4-dihydroisoquinolin-2(1H)-yl)methyl)benzoic acid* (**B-1**). Yellow solid (yield 90%). ^1^H-NMR (600 MHz, DMSO-*d*_6_) *δ* 11.54 (brs, 1H, COOH), 8.02 (d, *J* = 8.2 Hz, 2H), 7.82 (d, *J* = 8.2 Hz, 2H), 7.28-7.15 (m, 4H), 4.53 (d, *J* = 8.5 Hz, 2H), 4.29 (d, *J* = 18.0 Hz, 2H), 3.63 (s, 2H), 3.03 (s, 2H). ^13^C-NMR (150 MHz, DMSO-*d*_6_) *δ* 166.9, 134.5, 131.8, 131.7, 131.5, 129.7, 128.6, 128.3, 127.7, 126.7, 126.6, 57.8, 51.5, 48.6, 39.5, 24.9. ESI-MS *m/z* 268.1 [M + H]^+^.

*4-((6-bromo-3,4-dihydroisoquinolin-2(1H)-yl)methyl)benzoic acid* (**B-2**). Yellow solid (yield 92.3%). ^1^H-NMR (600 MHz, DMSO-*d*_6_) *δ* 11.54 (brs, 1H, COOH), 8.02 (d, *J* = 7.9 Hz, 2H), 7.83-7.77 (m, 2H), 7.49 (s, 1H), 7.42 (d, *J* = 8.3 Hz, 1H), 7.16 (d, *J* = 8.3 Hz, 1H), 4.52 (d, *J* = 18.9 Hz, 2H), 4.26 (s, 2H), 3.33-3.20 (m, 3H), 3.06 (s, 1H). ^13^C-NMR (150 MHz, DMSO-*d*_6_) *δ* 166.9, 134.3, 131.8, 131.6, 131.1, 129.7, 129.5, 128.9, 127.8, 120.6, 57.7, 51.0, 48.1, 39.5, 24.6. ESI-MS *m/z* 346.0 [M + H]^+^.

*4-((7-bromo-3,4-dihydroisoquinolin-2(1H)-yl)methyl)benzoic acid* (**B-3**). White solid (yield 87.5%). ^1^H-NMR (600 MHz, DMSO-*d*_6_) *δ* 11.66 (brs, 1H, COOH), 8.02 (d, *J* = 8.1 Hz, 2H), 7.81 (d, *J* = 8.1 Hz, 2H), 7.50-7.42 (m, 2H), 7.20 (d, *J* = 8.2 Hz, 1H), 4.58-4.44 (m, 2H), 4.28 (s, 2H), 3.63 (d, *J* = 10.7 Hz, 2H), 3.34-3.17 (m, 2H). ^13^C-NMR (150 MHz, DMSO-*d*_6_) *δ* 166.9, 134.3, 131.7, 131.6, 131.0, 130.7, 130.4, 129.6, 129.3, 119.3, 57.7, 50.8, 48.3, 39.5, 24.4. ESI-MS *m/z* 346.0 [M + H]^+^.

*4-((6-methoxy-3,4-dihydroisoquinolin-2(1H)-yl)methyl)benzoic acid* (**B-4**). White solid (yield, 87.7%). ^1^H-NMR (600 MHz, DMSO-*d*_6_) *δ* 7.83 (d, *J* = 8.0 Hz, 2H), 7.24 (d, *J* = 8.0 Hz, 2H), 6.91-6.87 (m, 2H), 6.61 (d, *J* = 2.3 Hz, 1H), 3.69 (d, *J* = 2.2 Hz, 5H), 3.61 (s, 2H), 2.87 (t, *J* = 5.8 Hz, 2H), 2.78 (t, *J* = 5.8 Hz, 2H). ^13^C-NMR (150 MHz, DMSO-*d*_6_) *δ* 170.0, 157.4, 139.4, 135.3, 129.0, 127.5, 127.3, 127.0, 126.9, 113.5, 111.8, 61.9, 55.0, 54.9, 50.2, 39.5, 29.0. ESI-MS *m/z* 298.1 [M + H]^+^.

*4-((6,7-dimethoxy-3,4-dihydroisoquinolin-2(1H)-yl)methyl)benzoic acid* (**B-5**). White solid (yield, 79.9%). ^1^H-NMR (600 MHz, DMSO-*d*_6_) *δ* 7.83 (d, *J* = 7.8 Hz, 2H), 7.24 (d, *J* = 7.8 Hz, 2H), 6.65 (s, 1H), 6.61 (s, 1H), 3.72 (s, 2H), 3.68 (d, *J* = 5.2 Hz, 8H), 2.87 (t, *J* = 5.8 Hz, 2H), 2.71 (d, *J* = 5.8 Hz, 2H). ^13^C-NMR (150 MHz, DMSO-*d*_6_) *δ* 170.0, 147.1, 146.9, 146.8, 129.0, 127.6, 126.7, 126.6, 125.8, 112.4, 109.7, 61.9, 55.5, 55.4, 50.2, 39.5, 28.3. ESI-MS *m/z* 328.1 [M + H]^+^.

#### 3.2.3. General Procedure for the Synthesis of **1**–**30**

The intermediates **B** (0.374 mmol, 1 equiv.) were dissolved in 5 mL DMF in a vial. 2-(7-azabenzotriazol-1-yl)-*N*,*N*,*N*’,*N*’-tetramethyluronium hexafluorophosphate (HATU) (156.4 mg, 0.411 mmol, 1.1 equiv.) were added to the reaction mixture. A series of corresponding amines or others ((4-fluorophenyl)hydrazine, 2-bromophenol and 2-bromobenzenethiol) (0.374 mmol, 1 equiv.) and DIPEA (193.3 mg, 1.496 mmol, 4 equiv.) were made in a separate vial. This solution was added to the reaction mixture drop wise and the reaction mixture was stirred overnight at room temperature. Upon completion of the reaction as detected by TLC, water (10 mL) was added and the product was extracted with EtOAc (3 × 10 mL). The combined organic layers were dried using MgSO_4_, then concentrated under vacuum, and the residues were purified by flash chromatography using PE:EtOAc = 2:1 as an eluent to give the target compounds **1**–**30**.

*N-(2-(diethylamino)ethyl)-4-((3,4-dihydroisoquinolin-2(1H)-yl)methyl)benzamide* (**1**). Yellow oil (yield 62.5%). ^1^H-NMR (600 MHz, DMSO-*d*_6_) *δ* 8.34 (t, *J* = 5.8 Hz, 1H), 7.80 (d, *J* = 8.2 Hz, 2H), 7.44 (d, *J* = 8.2 Hz, 2H), 7.13-7.06 (m, 3H), 6.99 (d, *J* = 7.3 Hz, 1H), 3.69 (s, 2H), 3.53 (s, 2H), 3.32-3.29 (m, 2H), 2.81 (t, *J* = 5.8 Hz, 2H), 2.67 (t, *J* = 5.8 Hz, 2H), 2.57-2.50 (m, 6H), 0.97 (t, *J* = 7.1 Hz, 6H). ^13^C-NMR (150 MHz, DMSO-*d*_6_) *δ* 165.9, 141.7, 134.7, 134.1, 133.4, 128.5, 127.1, 126.3, 126.0, 125.5, 61.4, 55.4, 51.5, 50.3, 46.8, 39.5, 37.5, 28.7, 11.9. ESI-MS *m*/*z* 366.2 [M + H]^+^. HR-ESIMS: [M + H]^+^ calcd for C_23_H_32_N_3_O^+^ 366.2545, found 366.2549.

*4-((3,4-dihydroisoquinolin-2(1H)-yl)methyl)-N-(2-(diisopropylamino)ethyl)benzamide* (**2**). Yellow oil (yield 54.7%). ^1^H-NMR (600 MHz, DMSO-*d*_6_) *δ* 8.34 (t, *J* = 5.8 Hz, 1H), 7.81 (d, *J* = 8.2 Hz, 2H), 7.44 (d, *J* = 8.2 Hz, 2H), 7.12-7.06 (m, 3H), 6.99 (d, *J* = 7.3 Hz, 1H), 3.69 (s, 2H), 3.53 (s, 2H), 3.21-3.17 (m, 2H), 3.00-2.94 (m, 2H), 2.81 (t, *J* = 5.8 Hz, 2H), 2.67 (t, *J* = 5.8 Hz, 2H), 2.53-2.50 (m, 2H), 0.98 (d, *J* = 6.6 Hz, 12H). ^13^C-NMR (150 MHz, DMSO-*d*_6_) *δ* 165.8, 141.6, 134.7, 134.0, 133.4, 128.5, 127.1, 126.3, 126.0, 125.5, 61.4, 55.4, 50.3, 48.5, 44.2, 41.0, 39.5, 28.7, 20.7. ESI-MS *m*/*z* 394.2 [M + H]^+^. HR-ESIMS: [M + H]^+^ calcd for C_25_H_36_N_3_O^+^ 394.2858, found 394.2853.

*4-((3,4-dihydroisoquinolin-2(1H)-yl)methyl)-N-(2-(dimethylamino)ethyl)benzamide* (**3**). Yellow solid (yield 55.5%). ^1^H-NMR (600 MHz, DMSO-*d*_6_) *δ* 8.35 (t, *J* = 5.7 Hz, 1H), 7.81 (d, *J* = 8.2 Hz, 2H), 7.44 (d, *J* = 8.2 Hz, 2H), 7.13-7.06 (m, 3H), 6.99 (d, *J* = 7.3 Hz, 1H), 3.69 (s, 2H), 3.53 (s, 2H), 2.81 (t, *J* = 5.8 Hz, 2H), 2.67 (t, *J* = 5.8 Hz, 2H), 2.50 (d, *J* = 1.8 Hz, 1H), 2.50 (d, *J* = 1.8 Hz, 1H), 2.41 (t, *J* = 6.8 Hz, 2H), 2.19 (s, 6H). ^13^C-NMR (150 MHz, DMSO-*d*_6_) *δ* 166.0, 141.7, 134.7, 134.1, 133.3, 128.5, 127.2, 126.4, 126.0, 125.5, 61.4, 58.2, 55.4, 50.3, 45.2, 39.5, 37.3, 28.7. ESI-MS *m*/*z* 338.2 [M + H]^+^. HR-ESIMS: [M + H]^+^ calcd for C_21_H_28_N_3_O^+^ 338.2232, found 338.2236.

*4-((3,4-dihydroisoquinolin-2(1H)-yl)methyl)-N-(2-(ethylamino)ethyl)benzamide* (**4**). Yellow solid (yield 60%). ^1^H-NMR (600 MHz, DMSO-*d*_6_) *δ* 8.39 (t, *J* = 5.6 Hz, 1H), 7.82 (d, *J* = 8.2 Hz, 2H), 7.43 (d, *J* = 8.2 Hz, 2H), 7.13-7.06 (m, 3H), 6.99 (d, *J* = 7.3 Hz, 1H), 3.69 (s, 2H), 3.53 (s, 2H), 2.81 (t, *J* = 5.8 Hz, 2H), 2.69-2.65 (m, 4H), 2.56 (q, *J* = 7.1 Hz, 2H), 2.50 (d, *J* = 1.8 Hz, 2H), 2.50 (d, *J* = 1.8 Hz, 1H), 1.01 (t, *J* = 7.1 Hz, 3H). ^13^C-NMR (150 MHz, DMSO-*d*_6_) *δ* 166.1, 141.7, 134.7, 134.1, 133.4, 128.5, 128.4, 127.2, 126.4, 126.0, 125.5, 61.4, 55.4, 50.3, 48.5, 43.3, 39.5, 28.7, 15.1. ESI-MS *m*/*z* 338.2 [M + H]^+^. HR-ESIMS: [M + H]^+^ calcd for C_21_H_28_N_3_O^+^ 338.2232, found 338.2213.

*4-((3,4-dihydroisoquinolin-2(1H)-yl)methyl)-N-(2,2-dimethoxyethyl)benzamide* (**5**). Yellow oil (yield 64.3%). ^1^H-NMR (600 MHz, DMSO-*d*_6_) *δ* 8.52 ((t, *J* = 5.8 Hz, 1H), 7.84 (d, *J* = 8.2 Hz, 2H), 7.45 (d, *J* = 8.2 Hz, 2H), 7.13-7.05 (m, 3H), 6.99 (d, *J* = 7.3 Hz, 1H), 4.52 (t, *J* = 5.5 Hz, 1H), 3.69 (s, 2H), 3.53 (s, 2H), 3.38-3.32 (m, 6H), 2.81 (t, *J* = 5.8 Hz, 2H), 2.69-2.65 (m, 2H). ^13^C-NMR (150 MHz, DMSO-*d*_6_) *δ* 166.2, 141.9, 134.7, 134.1, 133.0, 128.5, 128.5, 127.3, 126.4, 126.0, 125.5, 101.9, 61.4, 55.4, 53.2, 53.2, 50.3, 41.1, 39.5, 28.7. ESI-MS *m*/*z* 355.1 [M + H]^+^. HR-ESIMS: [M + H]^+^ calcd for C_21_H_27_N_2_O_3_^+^ 355.2022, found 355.2026.

*4-((3,4-dihydroisoquinolin-2(1H)-yl)methyl)-N-(2-morpholinoethyl)benzamide* (**6**). Yellow solid (yield 57%). ^1^H-NMR (600 MHz, DMSO-*d*_6_) *δ* 8.37 (t, *J* = 5.6 Hz, 1H), 7.80 (d, *J* = 8.2 Hz, 2H), 7.44 (d, *J* = 8.2 Hz, 2H), 7.13-7.04 (m, 3H), 6.99 (d, *J* = 7.3 Hz, 1H), 3.69 (s, 2H), 3.57 (t, *J* = 4.5 Hz, 4H), 3.53 (s, 2H), 3.38 (q, *J* = 6.6 Hz, 2H), 2.81 (t, *J* = 5.8 Hz, 2H), 2.67 (t, *J* = 5.8 Hz, 2H), 2.50 (s, 1H), 2.46 (t, *J* = 6.9 Hz, 2H), 2.41 (s, 3H). ^13^C-NMR (150 MHz, DMSO-*d*_6_) *δ* 166.0, 141.8, 134.7, 134.1, 133.3, 128.5, 128.5, 127.2, 126.4, 126.0, 125.5, 66.2, 61.4, 57.4, 55.4, 53.3, 50.3, 39.5, 36.5, 28.7. ESI-MS *m*/*z* 380.2 [M + H]^+^. HR-ESIMS: [M + H]^+^ calcd for C_23_H_30_N_3_O_2_^+^ 380.2338, found 380.2330.

*4-((3,4-dihydroisoquinolin-2(1H)-yl)methyl)-N-(2-(pyrrolidin-1-yl)ethyl)benzamide* (**7**). White solid (yield 60%). ^1^H-NMR (600 MHz, DMSO-*d*_6_) *δ* 8.39 (t, *J* = 5.6 Hz, 1H), 7.81 (d, *J* = 8.2 Hz, 2H), 7.44 (d, *J* = 8.2 Hz, 2H), 7.13-7.06 (m, 3H), 6.99 (d, *J* = 7.3 Hz, 1H), 3.69 (s, 2H), 3.53 (s, 2H), 3.37 (q, *J* = 6.8 Hz, 2H), 2.81 (t, *J* = 5.8 Hz, 2H), 2.67 (t, *J* = 5.8 Hz, 2H), 2.56 (t, *J* = 7.0 Hz, 2H), 2.49-2.45 (m, 4H), 1.67 (p, *J* = 3.1 Hz, 4H). ^13^C-NMR (150 MHz, DMSO-*d*_6_) *δ* 165.9, 141.7, 134.7, 134.1, 133.3, 128.5, 127.2, 126.3, 126.0, 125.5, 61.4, 55.4, 54.9, 53.7, 50.3, 39.5, 38.6, 28.7, 23.1. ESI-MS *m*/*z* 337.1 [M + H]^+^. HR-ESIMS: [M + H]^+^ calcd for C_23_H_30_N_3_O^+^ 364.2389, found 364.2365.

*4-((3,4-dihydroisoquinolin-2(1H)-yl)methyl)-N-((1-ethylpyrrolidin-2-yl)methyl)benzamide* (**8**). Yellow oil (yield 74.5%). ^1^H-NMR (600 MHz, DMSO-*d*_6_) *δ* 8.34 (t, *J* = 5.7 Hz, 1H), 7.80 (d, *J* = 8.2 Hz, 2H), 7.44 (d, *J* = 8.2 Hz, 2H), 7.13-7.05 (m, 3H), 6.99 (d, *J* = 7.3 Hz, 1H), 3.69 (s, 2H), 3.53 (s, 2H), 3.43-3.38 (m, 1H), 3.08-3.00 (m, 2H), 2.87-2.82 (m, 1H), 2.81 (t, *J* = 5.8 Hz, 2H), 2.67 (t, *J* = 5.8 Hz, 2H), 2.57 (m, 1H), 2.27 (m, 1H), 2.15-2.08 (m, 1H), 1.77 (m, 1H), 1.67-1.55 (m, 3H), 1.04 (t, *J* = 7.2 Hz, 3H). ^13^C-NMR (150 MHz, DMSO-*d*_6_) *δ* 166.1, 141.7, 134.7, 134.1, 133.4, 128.5, 127.2, 126.3, 126.0, 125.5, 62.7, 61.4, 55.4, 53.3, 50.3, 48.2, 43.2, 39.5, 28.7, 28.6, 22.4, 13.9. ESI-MS *m*/*z* 378.2 [M + H]^+^. HR-ESIMS: [M + H]^+^ calcd for C_24_H_32_N_3_O^+^ 378.2545, found 378.2563.

*(4-((3,4-dihydroisoquinolin-2(1H)-yl)methyl)phenyl)(pyrrolidin-1-yl)methanone* (**9**). Yellow oil (yield 74%). ^1^H-NMR (600 MHz, DMSO-*d*_6_) *δ* 7.48 (d, *J* = 8.1 Hz, 2H), 7.41 (d, *J* = 8.1 Hz, 2H), 7.09 (m, 3H), 6.99 (d, *J* = 7.3 Hz, 1H), 3.67 (s, 2H), 3.54 (s, 2H), 3.48-3.39 (m, 4H), 2.81 (t, *J* = 5.8 Hz, 2H), 2.68 (t, *J* = 5.8 Hz, 2H), 1.88-1.83 (m, 2H), 1.82-1.77 (m, 2H). ^13^C-NMR (150 MHz, DMSO-*d*_6_) *δ* 168.2, 140.2, 135.9, 134.7, 134.1, 128.5, 128.4, 127.1, 126.4, 126.0, 125.5, 61.5, 55.4, 50.3, 49.0, 45.9, 39.5, 28.7, 26.0, 23.9. ESI-MS *m*/*z* 321.1 [M + H]^+^. HR-ESIMS: [M + H]^+^ calcd for C_21_H_25_N_2_O^+^ 321.1967, found 321.1997.

*(4-((3,4-dihydroisoquinolin-2(1H)-yl)methyl)phenyl)(morpholino)methanone* (**10**). Yellow oil (yield 68%). ^1^H-NMR (600 MHz, DMSO-*d*_6_) *δ* 7.43 (d, *J* = 8.1 Hz, 2H), 7.38 (d, *J* = 8.1 Hz, 2H), 7.13-7.06 (m, 3H), 7.00 (d, *J* = 7.2 Hz, 1H), 3.68 (s, 2H), 3.59 (s, 4H), 3.55 (s, 2H), 2.82 (t, *J* = 5.8 Hz, 2H), 2.68 (t, *J* = 5.8 Hz, 2H). ^13^C-NMR (150 MHz, DMSO-*d*_6_) *δ* 169.1, 140.1, 134.7, 134.2, 134.1, 128.6, 128.5, 127.1, 126.4, 126.0, 125.5, 66.1, 61.4, 55.4, 50.2, 39.5, 28.7. ESI-MS *m*/*z* 337.1 [M + H]^+^. HR-ESIMS: [M + H]^+^ calcd for C_21_H_25_N_2_O_2_^+^ 337.1916, found 337.1909.

*(4-((6-bromo-3,4-dihydroisoquinolin-2(1H)-yl)methyl)phenyl)(pyrrolidin-1-yl)methanone* (**11**). Yellow oil (yield 64%). ^1^H-NMR (600 MHz, DMSO-*d*_6_) *δ* 7.48 (d, *J* = 8.1 Hz, 2H), 7.40 (d, *J* = 8.1 Hz, 2H), 7.32 (d, *J* =2.1Hz, 1H), 7.26 (dd, *J* = 8.2, 2.1Hz, 1H), 6.99 (d, *J* = 8.2 Hz, 1H), 3.68 (s, 2H), 3.50 (s, 2H), 3.45 (t, *J* = 6.7 Hz, 2H), 3.38 (t, *J* = 6.7 Hz, 2H), 2.82 (t, *J* = 5.8 Hz, 2H), 2.66 (t, *J* = 5.8 Hz, 2H), 1.88-1.83 (m, 2H), 1.82-1.77 (m. 2H). ^13^C-NMR (150 MHz, DMSO-*d*_6_) *δ* 168.1, 140.0, 137.1, 135.9, 134.2, 131.0, 128.6, 128.4, 128.3, 127.1, 118.8, 61.2, 54.8, 49.7, 49.0, 45.9, 39.5, 28.4, 26.0, 23.9. ESI-MS *m*/*z* 399.0 [M + H]^+^. HR-ESIMS: [M + H]^+^ calcd for C_21_H_24_BrN_2_O^+^ 399.1072, found 399.1056.

*(4-((7-bromo-3,4-dihydroisoquinolin-2(1H)-yl)methyl)phenyl)(pyrrolidin-1-yl)methanone* (**12**). Yellow oil (yield 65%). ^1^H-NMR (600 MHz, DMSO-*d*_6_) *δ* 7.48 (d, *J* = 7.8 Hz, 2H), 7.40 (d, *J* = 7.8 Hz, 2H), 7.29 (d, *J* = 8.2 Hz, 1H), 7.25 (s, 1H), 7.07 (d, *J* = 8.2 Hz, 1H), 3.67 (s, 2H), 3.55 (s, 2H), 3.46 (t, *J* = 6.7 Hz, 2H), 3.39 (t, *J* = 6.7 Hz, 2H), 2.77 (t, *J* = 5.7 Hz, 2H), 2.67 (t, *J* = 5.7 Hz, 2H), 1.89-1.83 (m, 2H), 1.82-1.77 (m, 2H). ^13^C-NMR (150 MHz, DMSO-*d*_6_) *δ* 168.1, 140.0, 137.5, 135.9, 133.6, 130.7, 129.0, 128.8, 128.3, 127.1, 118.3, 61.1, 54.8, 49.8, 48.9, 45.9, 39.5, 28.1, 26.0, 23.9. ESI-MS *m*/*z* 399.0 [M + H]^+^. HR-ESIMS: [M + H]^+^ calcd for C_21_H_24_BrN_2_O^+^ 399.1072, found 399.1062.

*(4-((6-methoxy-3,4-dihydroisoquinolin-2(1H)-yl)methyl)phenyl)(pyrrolidin-1-yl)methanone* (**13**). Brown oil (yield 71%). ^1^H-NMR (600 MHz, DMSO-*d*_6_) *δ* 7.48 (d, *J* = 8.0 Hz, 2H), 7.40 (d, *J* = 8.0 Hz, 2H), 6.91 (d, *J* = 9.2 Hz, 1H), 6.67 (d, *J* = 6.6 Hz, 2H), 3.69 (s, 3H), 3.66 (s, 2H), 3.48-3.44 (m, 4H), 3.39 (t, *J* = 6.5 Hz, 2H), 2.79 (t, *J* = 5.8 Hz, 2H), 2.65 (t, *J* = 5.8 Hz, 2H), 1.88-1.83 (m, 2H), 1.82-1.77 (m, 2H). ^13^C-NMR (150 MHz, DMSO-*d*_6_) *δ* 168.1, 157.5, 140.2, 135.9, 135.2, 128.3, 127.3, 127.1, 126.8, 113.0, 111.9, 61.5, 54.9, 50.2, 48.9, 45.9, 39.5, 29.0, 26.0, 23.9. ESI-MS *m*/*z* 351.1 [M + H]^+^. HR-ESIMS: [M + H]^+^ calcd for C_22_H_27_N_2_O_2_^+^ 351.2073, found 351.2079.

*(4-((6,7-dimethoxy-3,4-dihydroisoquinolin-2(1H)-yl)methyl)phenyl)(pyrrolidin-1-yl)methanone* (**14**). Yellow oil (yield 60.3%). ^1^H-NMR (600 MHz, DMSO-*d*_6_) *δ* 7.48 (d, *J* = 8.0 Hz, 2H), 7.40 (d, *J* = 8.0 Hz, 2H), 6.66 (s, 1H), 6.58 (s, 1H), 3.69 (s, 3H), 3.66 (s, 5H), 3.46 (t, *J* = 6.9 Hz, 2H), 3.43 (s, 2H), 3.39 (t, *J* = 6.5 Hz, 2H), 2.73 (t, *J* = 5.6 Hz, 2H), 2.66 (t, *J* = 5.6 Hz, 2H), 1.88-1.84 (m, 2H), 1.82-1.77 (m, 2H). ^13^C-NMR (151 MHz, DMSO-*d*_6_) *δ* 168.1, 147.2, 146.9, 140.3, 135.9, 128.4, 127.1, 126.4, 125.7, 111.8, 109.9, 61.6, 55.5, 55.4, 55.0, 50.6, 49.0, 45.9, 28.3, 26.0, 23.9. ESI-MS *m*/*z* 381.1 [M + H]^+^. HR-ESIMS: [M + H]^+^ calcd for C_23_H_29_N_2_O_3_^+^ 381.2178, found 381.2170.

*4-((3,4-dihydroisoquinolin-2(1H)-yl)methyl)-N-phenylbenzamide* (**15**). White solid (yield 56%). ^1^H-NMR (600 MHz, DMSO-*d*_6_) *δ* 10.22 (brs, 1H, NH), 7.93 (d, *J* = 8.2 Hz, 2H), 7.77 (d, *J* = 7.6 Hz, 2H), 7.52 (d, *J* = 8.2 Hz, 2H), 7.37-7.32 (m, 2H), 7.14-7.06 (m, 4H), 7.01 (s, 1H), 3.73 (s, 2H), 3.56 (s, 2H), 2.83 (t, *J* = 5.9 Hz, 2H), 2.70 (t, *J* = 5.9 Hz, 2H). ^13^C-NMR (150 MHz, DMSO-*d*_6_) *δ* 165.5, 142.4, 139.2, 134.7, 134.1, 133.7, 128.6, 128.6, 128.5, 127.8, 126.4, 126.1, 125.5, 123.7, 120.4, 61.4, 55.5, 50.3, 39.5, 28.7. ESI-MS *m*/*z* 343.1 [M + H]^+^. HR-ESIMS: [M + H]^+^ calcd for C_23_H_23_N_2_O^+^ 343.1810, found 343.1827.

*4-((3,4-dihydroisoquinolin-2(1H)-yl)methyl)-N-(2-fluorophenyl)benzamide* (**16a**). Yellow oil (yield 37%). ^1^H-NMR (600 MHz, DMSO-*d*_6_) *δ* 10.09 (brs, 1H, NH), 7.96 (d, *J* = 8.2 Hz, 2H), 7.61-7.57 (m, 1H), 7.52 (d, *J* = 8.2 Hz, 2H), 7.32-7.25 (m, 2H), 7.24-7.2 (m, 1H), 7.15-7.06 (m, 3H), 7.01 (d, *J* = 7.3 Hz, 1H), 3.74 (s, 2H), 3.56 (s, 2H), 2.83 (t, *J* = 5.8 Hz, 2H), 2.70 (t, *J* = 5.8 Hz, 2H). ^13^C-NMR (150 MHz, DMSO-*d*_6_) *δ* 165.3, 156.7, 155.0, 142.7, 134.7, 134.1, 132.7, 128.6, 128.5, 127.9, 127.2, 126.4, 126.0, 125.5, 124.3, 124.3, 115.9, 115.8, 61.4, 55.4, 50.3, 39.5, 28.7. ESI-MS *m*/*z* 361.0 [M + H]^+^. HR-ESIMS: [M + H]^+^ calcd for C_23_H_22_FN_2_O^+^ 361.1716, found 361.1757.

*4-((3,4-dihydroisoquinolin-2(1H)-yl)methyl)-N-(3-fluorophenyl)benzamide* (**17**). Yellow oil (yield 71.6%). ^1^H-NMR (600 MHz, DMSO-*d*_6_) *δ* 10.40 (brs, 1H, NH), 7.93 (d, *J* = 8.2 Hz, 2H), 7.78-7.73 (m, 1H), 7.56 (dd, *J* = 8.1, 1.9 Hz, 1H), 7.53 (d, *J* = 8.1 Hz, 2H), 7.38 (q, *J* = 8.1 Hz, 1H), 7.13-7.07 (m, 3H), 7.00 (d, *J* = 7.2 Hz, 1H), 6.96-6.89 (m, 1H), 3.73 (s, 2H), 3.56 (s, 2H), 2.83 (t, *J* = 5.7 Hz, 2H), 2.71-2.68 (m, 2H). ^13^C-NMR (150 MHz, DMSO-*d*_6_) *δ* 165.7, 162.9, 161.3, 142.7, 141.1, 141.0, 134.7, 134.1, 133.4, 130.3, 130.2, 128.6, 128.5, 127.8, 126.4, 126.0, 125.5, 115.9, 115.9, 61.4, 55.5, 50.3, 39.5, 28.7. ESI-MS *m*/*z* 360.9 [M + H]^+^. HR-ESIMS: [M + H]^+^ calcd for C_23_H_22_FN_2_O^+^ 361.1716, found 361.1722.

*4-((3,4-dihydroisoquinolin-2(1H)-yl)methyl)-N-(4-fluorophenyl)benzamide* (**18**). White solid (yield 60%). ^1^H-NMR (600 MHz, DMSO-*d*_6_) *δ* 10.30 (brs, 1H, NH), 7.93 (d, *J* = 8.2 Hz, 2H), 7.79 (m, 2H), 7.52 (d, *J* = 8.2 Hz, 2H), 7.22–7.17 (m, 2H), 7.10 (m, 3H), 7.00 (d, *J* = 7.3 Hz, 1H), 3.73 (s, 2H), 3.56 (s, 2H), 2.83 (t, *J* = 5.8 Hz, 2H), 2.69 (t, *J* = 5.8 Hz, 2H). ^13^C-NMR (150 MHz, DMSO-*d*_6_) *δ* 165.4, 159.1, 157.5, 142.5, 135.6, 135.6, 134.7, 134.1, 133.6, 128.6, 128.5, 127.8, 126.4, 126.1, 125.6, 122.2, 122.1, 115.3, 115.2, 61.4, 55.5, 50.3, 39.5, 28.7. ESI-MS *m*/*z* 361.0 [M + H]^+^. HR-ESIMS: [M + H]^+^ calcd for C_23_H_22_FN_2_O^+^ 361.1716, found 361.1707.

*4-((3,4-dihydroisoquinolin-2(1H)-yl)methyl)-N-(4-fluorobenzyl)benzamide* (**19**). Yellow solid (yield 61.2%). ^1^H-NMR (600 MHz, DMSO-*d*_6_) *δ* 9.03 (t, *J* = 6.0 Hz, 1H), 7.87 (d, *J* = 8.3 Hz, 2H), 7.46 (d, *J* = 8.3 Hz, 2H), 7.40-7.31 (m, 2H), 7.17-7.13 (m, 2H), 7.12-7.05 (m, 3H), 6.99 (d, *J* = 7.3 Hz, 1H), 4.46 (d, *J* = 6.0 Hz, 2H), 3.69 (s, 2H), 3.54 (s, 2H), 2.81 (t, *J* = 5.8 Hz, 2H), 2.67 (t, *J* = 5.8 Hz, 2H). ^13^C-NMR (150 MHz, DMSO-*d*_6_) *δ* 166.1, 161.9, 160.3, 142.0, 135.9, 135.9, 134.7, 134.0, 133.0, 129.2, 129.2, 128.5, 128.5, 127.3, 126.3, 126.0, 125.5, 115.0, 114.9, 61.4, 55.4, 50.3, 41.9, 39.5, 28.7. ESI-MS *m*/*z* 375.1 [M + H]^+^. HR-ESIMS: [M + H]^+^ calcd for C_24_H_24_FN_2_O^+^ 375.1873, found 375.1890.

*4-((3,4-dihydroisoquinolin-2(1H)-yl)methyl)-N2019-(4-fluorophenyl)benzohydrazide* (**20**). Yellow solid (yield 50%). ^1^H-NMR (600 MHz, DMSO-*d*_6_) *δ* 10.39 (d, *J* = 3.0 Hz, 1H), 7.92 (d, *J* = 3.0 Hz, 1H), 7.90 (d, *J* = 8.1 Hz, 2H), 7.49 (d, *J* = 8.1 Hz, 2H), 7.16-7.05 (m, 3H), 7.00 (t, *J* = 8.3 Hz, 3H), 6.84-6.73 (m, 2H), 3.71 (s, 2H), 3.55 (s, 2H), 2.82 (t, *J* = 5.8 Hz, 2H), 2.69 (t, *J* = 5.8 Hz, 2H). ^13^C-NMR (150 MHz, DMSO-*d*_6_) *δ* 166.3, 156.7, 155.1, 146.2, 142.5, 134.7, 134.1, 131.7, 128.7, 128.5, 127.4, 126.4, 126.1, 125.6, 115.3, 115.2, 113.6, 113.5, 61.4, 55.5, 50.3, 39.5, 28.7. ESI-MS *m*/*z* 376.0 [M + H]^+^. HR-ESIMS: [M + H]^+^ calcd for C_23_H_23_FN_3_O^+^ 376.1825, found 376.1845.

*N-(4-chloro-2-fluorophenyl)-4-((3,4-dihydroisoquinolin-2(1H)-yl)methyl)benzamide* (**21**). Yellow oil (yield 34%). ^1^H-NMR (600 MHz, DMSO-*d*_6_) *δ* 8.71 (dd, *J* = 4.3, 1.2 Hz, 1H), 8.50 (dd, *J* = 8.3, 1.2 Hz, 1H), 7.92 (d, *J* = 8.3 Hz, 2H), 7.50 (d, *J* = 8.3 Hz, 2H), 7.49-7.47 (m, 1H), 7.13-7.07 (m, 3H), 7.00 (d, *J* = 7.3 Hz, 1H), 3.76 (s, 2H), 3.58 (s, 2H), 2.84 (t, *J* = 5.8 Hz, 2H), 2.73 (t, *J* = 5.8 Hz, 2H). ^13^C-NMR (150 MHz, DMSO-*d*_6_) *δ* 167.2, 150.7, 139.6, 134.6, 133.9, 129.6, 129.4, 128.8, 128.7, 128.5, 126.4, 126.1, 125.6, 120.5, 61.2, 55.3, 50.2, 39.5, 28.5. ESI-MS *m*/*z* 395.0 [M + H]^+^. HR-ESIMS: [M + H]^+^ calcd for C_23_H_21_ClFN_2_O^+^ 395.1326, found 395.1301.

*N-(4-bromo-2-fluorophenyl)-4-((3,4-dihydroisoquinolin-2(1H)-yl)methyl)benzamide* (**22**). White solid (yield 40%). ^1^H-NMR (600 MHz, DMSO-*d*_6_) *δ* 10.19 (brs, 1H, NH), 7.95 (d, *J* = 8.2 Hz, 2H), 7.66 (dd, *J* = 10.0, 2.1 Hz, 1H), 7.58 (t, *J* = 8.4 Hz, 1H), 7.52 (d, *J* = 8.2 Hz, 2H), 7.46-7.43 (m, 1H), 7.13-7.07 (m, 3H), 7.00 (d, *J* = 7.3 Hz, 1H), 3.73 (s, 2H), 3.56 (s, 2H), 2.83 (t, *J* = 5.8 Hz, 2H), 2.70 (t, *J* = 5.8 Hz, 2H). ^13^C-NMR (150 MHz, DMSO-*d*_6_) *δ* 165.3, 156.5, 154.8, 142.9, 134.7, 134.1, 132.4, 128.7, 128.5, 128.4, 128.0, 127.5, 126.4, 126.1, 125.6, 119.4, 119.2, 117.6, 117.6, 61.4, 55.5, 50.3, 39.5, 28.7. ESI-MS *m*/*z* 439.0 [M + H]^+^. HR-ESIMS: [M + H]^+^ calcd for C_23_H_21_BrFN_2_O^+^ 439.0821, found 439.0802.

*N-(2-bromophenyl)-4-((3,4-dihydroisoquinolin-2(1H)-yl)methyl)benzamide* (**23**). Yellow oil (yield 51%). ^1^H-NMR (600 MHz, DMSO-*d*_6_) *δ* 8.69 (dd, *J* = 4.4, 1.4 Hz, 1H), 8.48 (dd, *J* = 8.4, 1.4 Hz, 1H), 7.93 (d, *J* = 8.2 Hz, 2H), 7.50 (d, *J* = 8.2 Hz, 2H), 7.46 (dd, *J* = 8.4, 4.4 Hz, 1H), 7.14-7.07 (m, 3H), 7.01 (d, *J* = 7.3 Hz, 1H), 3.79 (s, 2H), 3.61 (s, 2H), 2.84 (t, *J* = 5.8 Hz, 2H), 2.75 (t, *J* = 5.8 Hz, 2H). ^13^C-NMR (150 MHz, DMSO-*d*_6_) *δ* 167.21, 150.59, 139.59, 134.60, 134.10, 133.79, 129.74, 129.39, 128.92, 128.61, 128.46, 126.38, 126.16, 125.58, 120.44, 61.10, 55.14, 50.19, 39.5, 28.35. ESI-MS *m*/*z* 421.0 [M + H]^+^. HR-ESIMS: [M + H]^+^ calcd for C_23_H_22_BrN_2_O^+^ 421.0916, found 421.0910.

*N-(3-bromophenyl)-4-((3,4-dihydroisoquinolin-2(1H)-yl)methyl)benzamide* (**24**). Yellow oil (yield 61.1%). ^1^H-NMR (600 MHz, DMSO-*d*_6_) *δ* 10.38 (brs, 1H, NH), 8.12 (d, *J* = 1.9 Hz, 1H), 7.95–7.92 (m, 2H), 7.76 (d, *J* = 7.9 Hz, 1H), 7.53 (d, *J* = 8.0 Hz, 2H), 7.31 (m, 2H), 7.13-7.07 (m, 3H), 7.00 (d, *J* = 7.2 Hz, 1H), 3.73 (s, 2H), 3.57 (s, 2H), 2.83 (t, *J* = 5.7 Hz, 2H), 2.70 (m, 2H). ^13^C-NMR (150 MHz, DMSO-*d*_6_) *δ* 165.7, 142.7, 140.9, 134.7, 134.1, 133.3, 130.6, 128.7, 128.6, 128.5, 127.8, 126.4, 126.2, 125.5, 122.5, 121.4, 119.0, 61.4, 55.5, 50.3, 39.5, 28.7. ESI-MS *m*/*z* 421.0 [M + H]^+^. HR-ESIMS: [M + H]^+^ calcd for C_23_H_22_BrN_2_O^+^ 421.0916, found 421.0915.

*N-(4-bromophenyl)-4-((3,4-dihydroisoquinolin-2(1H)-yl)methyl)benzamide* (**25**). White solid (yield 38%). ^1^H-NMR (600 MHz, DMSO-*d*_6_) *δ* 10.36 (brs, 1H, NH), 7.93 (d, *J* = 8.2 Hz, 2H), 7.80-7.73 (m, 2H), 7.53 (m, 4H), 7.14-7.07 (m, 3H), 7.00 (d, *J* = 7.3 Hz, 1H), 3.73 (s, 2H), 3.56 (s, 2H), 2.83 (t, *J* = 5.8 Hz, 2H), 2.69 (t, *J* = 5.8 Hz, 2H). ^13^C-NMR (150 MHz, DMSO-*d*_6_) *δ* 165.6, 142.6, 138.7, 134.7, 134.1, 133.5, 131.5, 128.7, 128.5, 127.8, 126.4, 126.1, 125.6, 122.2, 115.3, 61.4, 55.5, 50.3, 39.5, 28.7. ESI-MS *m*/*z* 421.0 [M + H]^+^. HR-ESIMS: [M + H]^+^ calcd for C_23_H_22_BrN_2_O^+^ 421.0916, found 421.0922.

*2-bromophenyl 4-((3,4-dihydroisoquinolin-2(1H)-yl)methyl)benzoate* (**26**). Yellow oil (yield 64%). ^1^H-NMR (600 MHz, DMSO-*d*_6_) *δ* 8.14 (d, *J* = 8.3 Hz, 2H), 7.78 (dd, *J* = 8.1, 1.5 Hz, 1H), 7.63 (d, *J* = 8.3 Hz, 2H), 7.52-7.49 (m, 1H), 7.46 (dd, *J* = 8.1, 1.5 Hz, 1H), 7.31-7.28 (m, 1H), 7.13-7.08 (m, 3H), 7.02 (d, *J* = 7.3 Hz, 1H), 3.78 (s, 2H), 3.58 (s, 2H), 2.84 (t, *J* = 5.8 Hz, 2H), 2.72 (t, *J* = 5.8 Hz, 2H). ^13^C-NMR (150 MHz, DMSO-*d*_6_) *δ* 163.6, 148.0, 145.7, 134.6, 134.0, 133.2, 130.1, 129.2, 128.5, 128.1, 127.0, 126.4, 126.0, 125.5, 124.5, 115.7, 61.3, 55.4, 50.3, 39.5, 28.7. ESI-MS *m*/*z* 422.0 [M + H]^+^. HR-ESIMS: [M + H]^+^ calcd for C_23_H_21_BrNO_2_^+^ 422.0756, found 422.0757.

*S-(2-bromophenyl) 4-((3,4-dihydroisoquinolin-2(1H)-yl)methyl)benzothioate* (**27**). Yellow oil (yield 61.3%). ^1^H-NMR (600 MHz, DMSO-*d*_6_) *δ* 7.99 (d, *J* = 8.3 Hz, 2H), 7.86 (dd, *J* = 7.9, 1.3 Hz, 1H), 7.71 (dd, *J* = 7.6, 1.7 Hz, 1H), 7.61 (d, *J* = 8.3 Hz, 2H), 7.54-7.51 (m, 1H), 7.49-7.46 (m, 1H), 7.13-7.07 (m, 3H), 7.01 (d, *J* = 7.3 Hz, 1H), 3.76 (s, 2H), 3.57 (s, 2H), 2.83 (t, *J* = 5.8 Hz, 2H), 2.70 (t, *J* = 5.8 Hz, 2H). ^13^C-NMR (150 MHz, DMSO-*d*_6_) *δ* 187.1, 145.9, 138.0, 134.6, 134.3, 134.0, 133.5, 132.1, 129.6, 129.4, 128.7, 128.5, 128.3, 127.4, 126.4, 126.0, 125.5, 61.2, 55.4, 50.3, 28.7. ESI-MS *m*/*z* 438.0 [M + H]^+^. HR-ESIMS: [M + H]^+^ calcd for C_23_H_21_BrNOS^+^ 438.0527, found 438.0557.

*N-(2-(1H-indol-3-yl)ethyl)-4-((3,4-dihydroisoquinolin-2(1H)-yl)methyl)benzamide* (**28**). ^1^H-NMR (600 MHz, DMSO-*d*_6_) *δ* 10.81 (brs, 1H, NH), 8.58 (t, *J* = 5.7 Hz, 1H), 7.83 (d, *J* = 8.2 Hz, 2H), 7.59 (d, *J* = 8.0 Hz, 1H), 7.44 (d, *J* = 8.2 Hz, 2H), 7.34 (d, *J* = 8.0 Hz, 1H), 7.18 (d, *J* = 2.2 Hz, 1H), 7.15-7.03 (m, 4H), 7.02-6.93 (m, 2H), 3.69 (s, 2H), 3.58-3.51 (m, 4H), 2.95 (t, *J* = 7.5 Hz, 2H), 2.82 (t, *J* = 5.8 Hz, 2H), 2.68 (t, *J* = 5.8 Hz, 2H). ^13^C-NMR (150 MHz, DMSO-*d*_6_) *δ* 166.0, 141.7, 136.3, 134.7, 134.1, 133.5, 128.5, 127.3, 127.2, 126.4, 126.0, 125.5, 122.6, 120.9, 118.3, 118.2, 111.9, 111.4, 61.4, 55.5, 50.3, 40.2, 39.5, 28.7, 25.2. ESI-MS *m*/*z* 410.1 [M + H]^+^. HR-ESIMS: [M + H]^+^ calcd for C_27_H_28_N_3_O^+^ 410.2232, found 410.2250.

*4-((3,4-dihydroisoquinolin-2(1H)-yl)methyl)-N-(2-(5-methyl-1H-indol-3-yl)ethyl)benzamide* (**29**). White solid (yield 63.2%). ^1^H-NMR (600 MHz, DMSO-*d*_6_) *δ* 10.67 (brs, 1H, NH), 8.57 (t, *J* = 5.6 Hz, 1H), 7.83 (d, *J* = 8.2 Hz, 2H), 7.44 (d, *J* = 8.2 Hz, 2H), 7.34 (s, 1H), 7.22 (d, *J* = 8.2 Hz, 1H), 7.16-7.04 (m, 4H), 6.99 (d, *J* = 7.2 Hz, 1H), 6.89 (dd, *J* = 8.2, 1.2 Hz, 1H), 3.69 (s, 2H), 3.53 (d, *J* = 7.8 Hz, 4H), 2.92 (t, *J* = 7.5 Hz, 2H), 2.82 (t, *J* = 5.8 Hz, 2H), 2.68 (t, *J* = 5.8 Hz, 2H), 2.35 (s, 3H). ^13^C-NMR (150 MHz, DMSO-*d*_6_) *δ* 166.0, 141.7, 134.7, 134.6, 134.1, 133.5, 128.5, 128.4, 127.6, 127.2, 126.5, 126.3, 126.0, 125.5, 122.7, 122.5, 118.0, 111.5, 111.1, 61.4, 55.4, 50.3, 40.4, 39.5, 28.7, 25.2, 21.3. ESI-MS *m*/*z* 424.2 [M + H]^+^. HR-ESIMS: [M + H]^+^ calcd for C_28_H_30_N_3_O^+^ 424.2389, found 424.2404.

*4-((3,4-dihydroisoquinolin-2(1H)-yl)methyl)-N-(2-(5-methoxy-1H-indol-3-yl)ethyl)benzamide* (**30**). White solid (yield 58%). ^1^H-NMR (600 MHz, DMSO-*d*_6_) *δ* 10.64 (brs, 1H, NH), 8.57 (t, *J* = 5.7 Hz, 1H), 7.83 (d, *J* = 8.3 Hz, 2H), 7.44 (d, *J* = 8.3 Hz, 2H), 7.22 (d, *J* = 8.8 Hz, 1H), 7.14 (d, *J* = 2.3 Hz, 1H), 7.13-7.06 (m, 3H), 7.05 (d, *J* = 2.3 Hz, 1H), 6.99 (d, *J* = 7.2 Hz, 1H), 6.71 (dd, *J* = 8.7, 2.4 Hz, 1H), 3.71 (s, 3H), 3.69 (s, 2H), 3.57–3.50 (m, 4H), 2.92 (t, *J* = 7.4 Hz, 2H), 2.82 (t, *J* = 5.8 Hz, 2H), 2.70–2.65 (m, 2H). ^13^C-NMR (150 MHz, DMSO-*d*_6_) *δ* 166.0, 153.0, 141.7, 134.7, 134.1, 133.5, 131.4, 128.5, 128.5, 127.7, 127.2, 126.4, 126.0, 125.5, 123.3, 112.0, 111.8, 111.1, 100.2, 61.4, 55.4, 55.3, 50.3, 48.6, 40.2, 39.5, 28.7, 25.2. ESI-MS *m*/*z* 440.2 [M + H]^+^. HR-ESIMS: [M + H]^+^ calcd for C_28_H_30_N_3_O_2_^+^ 440.2338, found 440.2324.

### 3.3. BChE and AChE Bioassay

BChE (EC 3.1.1.8, from human serum), AChE (EC 3.1.1.8, from human erythrocyte), butyrylthiocholine (BUC) iodide, and acetylthiocholine (ATC) iodide were purchased from Sigma-Aldrich (St. Louis, MO, USA). Enzyme was dissolved and pre-prepared at 2.0 U/mL. 10 μL of enzyme, 20 μL of 0.01 M 5,5′-dithiobis(2-nitrobenzoic acid), 10 μL of tested compound, and 40 μL of PBS were pre-incubated together for 5 min. Io initiate the reaction, 20 μL of 0.01 M BUC or ATC was added. The activity was determined by measuring the increase in absorbance at 410 nm at 37 °C in 2 min intervals using Tecan Spark multimode microplate reader (Mannedorf, Switzerland). The percentage of inhibition (I) was calculated according to the formula: I = (Ac − Ai)/Ac × 100%, with Ai and Ac representing the change in the absorbance in the presence of an inhibitor and without an inhibitor, respectively [28,29].

### 3.4. Kinetic Assay

Kinetic study of BChE inhibition was performed by using Ellman’s method [28,29] as described in Section 3.3.

### 3.5. Molecular Docking

The molecular docking procedure to study the binding mode of **23** against BChE was performed according to our previously reported protocol [28,29].

### 3.6. Molecular Dynamics Simulation

After the BChE:**23** complex was obtained by molecular docking, MD simulation was subsequently employed to probe this complex. The protonation states of ionizable residues in BChE were determined using the H++ program [48]. A periodic box of TIP3P [49] water molecules that extend 10 Å from the protein atoms was added. Then counter-ions were added to neutralize the simulation model. The AMBER force field (parm99SB) was used on the protein. The restrained electrostatic potential method [50] encoded in the AMBER suite of programs [51] at the RHF/6-31G* level was used to calculate the atom charges of **23**. Those already present in the AMBER force field covalent and nonbonded parameters were assigned for **23**.

All the MD simulations (at constant temperature and pressure and periodic boundary conditions) were conducted with SANDER program (time step = 2 fs). All hydrogen atom bonds in the simulation system were constrained by the SHAKE algorithm [52]. The particle-mesh Ewald method was used to calculate electrostatic interactions [53].

### 3.7. Binding Free Energy Calculation with MM-PBSA Method

The binding free energy of **23** with BChE was calculated using the MM-PBSA method encoded in the AMBER 10.0 program. Based on the 100 ns equilibrated dynamic trajectory, 1000 snapshots from the trajectory were extracted every 100 ps, and were used for the binding free energy calculation. The MMPBSA.PY module in AMBER 10.0 was employed to perform the calculation.

### 3.8. Determination of the Inhibitory Potency on Aβ_1–42_ Aggregation

The ThT-based fluorometric assay [54] was performed for the determination of compounds’ anti-Aβ_1–42_ aggregation potency. The detailed procedure can be found in Supplementary Materials.

### 3.9. Cell Viability Assay and Neuroprotective Activity Evaluation

The MTT method [55] was used to test the cytotoxicity and neuroprotective activity evaluation of compounds. The detailed procedure was included in Supplementary Materials.

### 3.10. Statistical Analyses

All of the experimental values were expressed as mean ± standard deviation (SD) of at least three independent experiments. GraphPad Prism 5.0 statistical software package (GraphPad Software, San Diego, CA, USA) was used to perform all of the statistical analysis.

## 4. Conclusions

In the present study, a series of novel selective BChE inhibitors were derived from the structural optimization of hit **1**. Compounds **9** and **23** displayed improved BChE inhibitory activity and good selectivity towards BChE. The current preliminary SAR study mainly concerned the effects of amide side-chain and substituents at tetrahydroisoquinoline on the BChE inhibitory activity. From the bioassay results, it could be summarized as follows: (1) for compounds **1**–**10**, tetrahydropyrrole ring could improve the BChE activity, (2) for compounds **11**–**14**, the substitution at C-6 and C-7 position of tetrahydroisoquinoline led to the decrease of BChE activity and selectivity, and (3) for compounds **15**–**30**, the substitution at C-2 of benzene ring should be optimal for BChE activity and amide group was found to be greatly important for the binding of inhibitor to BChE. Kinetic analysis together with molecular modeling studies suggested that these derivatives are a mixed-type of BChE inhibitors. Further, the selected compounds **9** and **23** displayed inhibitory activity against Aβ_1-42_ aggregation by a dose-dependent manner. Moreover, these two compounds are non-toxic towards SH-SY5Y cells even at 200 μM, indicating they are probably safe on SH-SY5Y cells. Of course, this discussion on safety needs more in vitro and even in vivo evaluations. More importantly, compounds **9** and **23** showed significantly in vitro protective activity against Aβ_1-42_-induced toxicity in SH-SY5Y cell model at 5 and 10 μM, and compound **23** even showed much better activity than the positive control EGCG at 10 μM. In summary, the present in vitro biological evaluation together with computational analyses provided a new valuable chemical template, which deserves further optimization for an anti-AD drug discovery.

## Figures and Tables

**Table 1:** The inhibitory activities of compounds 1–10 against ChEs.

No.	R	BChE			AChE
		IR ^a^(%)	IC_50_ (μM)	IR(%)	IC_50_ (μM)
**1**	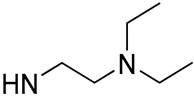	68.4	17.9 ± 1.86	26.6	-^b^
**2**	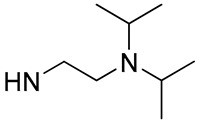	76.4	9.98 ± 0.89	56.1	21.46 ± 0.98
**3**	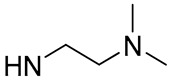	36.9	-	11.4	-
**4**	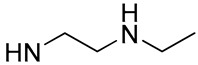	27.2	-	8.8	-
**5**	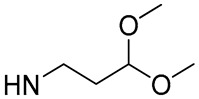	56.6	38.10 ± 1.68	2.7	-
**6**	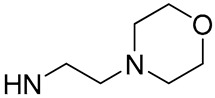	19.1	-	13.4	-
**7**	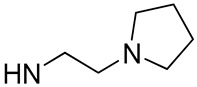	45.4	15.2 ± 2.33	4.4	-
**8**	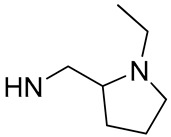	63.0	24.98 ± 1.27	13.3	-
**9**	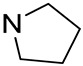	95.1	2.68 ± 0.28	29.1	-
**10**	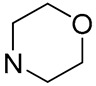	11.2	-	3.4	-
Tacrine		98.4	0.14 ± 0.01	96.2	0.11 ± 0.03

IC_50_ values are expressed as mean ± SD of three separate experiments. ^a^ Inhibition ratio (IR) against ChE at 50 μM. ^b^ Not tested.

**Table 2:** The inhibitory activities of compounds **11**–**14** against ChEs.

No.	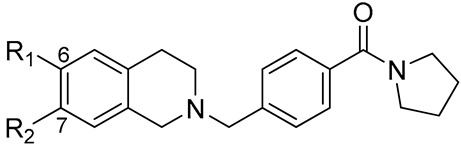	BChE IC_50_ (μM)	AChE IC_50_ (μM)
**11**	R_1_ = Br, R_2_ = H	8.38 ± 0.65	30.61 ± 1.82
**12**	R_1_ = H, R_2_ = Br	39.44 ± 2.25	>50
**13**	R_1_ = OMe, R_2_ = H	12.62 ± 0.88	12.13 ± 0.90
**14**	R_1_ = OMe, R_2_ = OMe	-	18.24 ± 0.86

IC_50_ values are expressed as mean ± SD of three separate experiments.

**Table 3:** The inhibitory activities of compounds **15**–**30** against ChEs.

No.	R	BChE		AChE	
		IR ^a^(%)	IC_50_ (μM)	IR(%)	IC_50_ (μM)
**15**	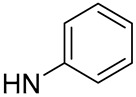	23.7	-^b^	8	^-^
**16**	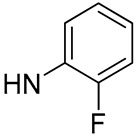	74.7	6.93 ± 0.48	2.1	-
**17**	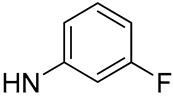	10.0	-	7.7	-
**18**	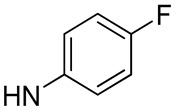	14.1	-	7.0	-
**19**	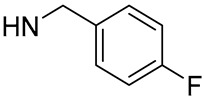	83.9	14.78 ± 0.58	2.3	-
**20**	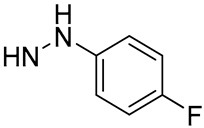	13.2	-	21.8	-
**21**	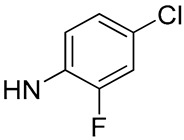	38.6	-	5	-
**22**	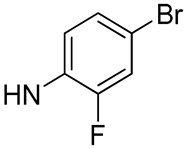	15.4	-	28.3	-
**23**	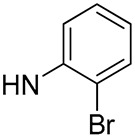	97.9	0.83 ± 0.08	22.7	-
**24**	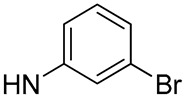	35.1	-	21.6	-
**25**	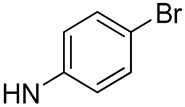	8.1	-	40.6	-
**26**	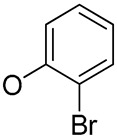	11	-	26.2	-
**27**	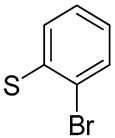	1.5	-	22.8	-
**28**	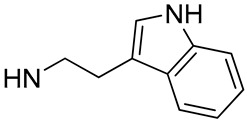	1.3	-	8.4	-
**29**	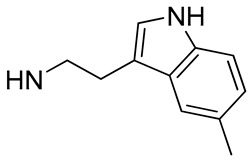	6.5	-	4.6	-
**30**	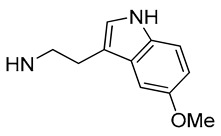	1.1	-	8.3	-

IC_50_ values are expressed as mean ± SD of three separate experiments. ^a^ Inhibition ratio (IR) against ChE at 50 μM. ^b^ Not tested.

**Table 4:** The binding free energy calculated by MM-PBSA method. ΔG_gas_ represents the binding free energy in vacuum and ΔG_solv_ represents the solvation free energy changes. ΔG = ΔG_gas_ + ΔG_solv_. The data were shown in kcal/mol.

Inhibitor	ΔG_gas_	ΔG_solv_	ΔG
**23**	−59.52 ± 10.41	33.59 ± 9.06	−25.93 ± 5.07

**Table 5:** The hydrophobic interactions residues in BChE and their corresponding occupancy rates.

Residue	Occupancy Rate (%)	Residue	Occupancy Rate (%)
**D70**	79	**P285**	61
**W82**	100	**A328**	57
**G115**	58	**F329**	80
**G116**	56	**Y332**	78
**Y128**	68	**H438**	59
**E197**	55		

## Supplementary Materials

The following are available online, Figure S1, ^1^H, ^13^C, HR-ESIMS spectra for compounds **1**–**30**, methods for determination of the inhibitory potency on Aβ_1–42_ aggregation, Cell viability assay, neuroprotective activity evaluation.

## Abbreviations

Alzheimer’s disease	(AD)
acetylcholine	(ACh)
beta-amyloid	(Aβ)
cholinesterase	(ChE)
butyrylcholinesterase	(BChE)
acetylcholinesterase	(AChE)
catalytic active site	(CAS)
peripheral anionic site	(PAS)
structure-activity relationship	(SAR)
molecular dynamics	(MD)
Molecular Mechanics Poisson-Boltzmann Surface Area	(MM-PBSA)
root-mean-square deviations	(RMSDs)
thioflavin T	(ThT)
epigallocatechin gallate	(EGCG)
butyrylthiocholine	(BUC)
acetylthiocholine	(ATC)
