# Supplementary material for: Discovery of New Selective Butyrylcholinesterase (BChE) Inhibitors with Anti-Aβ Aggregation Activity: Structure-Based Virtual Screening, Hit Optimization and Biological Evaluation
Source: Molecules. 2019 Jul 15;24(14):2568. doi: 10.3390/molecules24142568 (PMC6680840; doi:10.3390/molecules24142568)

**Supporting Information for**

# Discovery of new selective butyrylcholinesterase (BChE) inhibitors with anti-Aβ aggregation activity: structure-based virtual screening, hit optimization and biological evaluation

Cheng-Shi Jiang[a,*], Yong-Xi Ge[a], Zhi-Qiang Cheng[a], Yin-Yin Wang[a], Hong-Rui Tao[a,b], Kongkai Zhu[a,*], Hua Zhang[a,*]

[a] *School of Biological Science and Technology, University of Jinan, Jinan 250022, China*

[b] *Shanghai Institute of Material Medica, Chinese Academy of Sciences, Shanghai 201203, China*

*Corresponding authors

E-mail addresses: bio_jiangcs@ujn.edu.cn (C.S. Jiang); hkhhh.k@163.com (K.K. Zhu), bio_zhangh@ujn.edu.cn (H. Zhang)

Tel: 0086-531-89736199, Fax: 0086-531-89736818

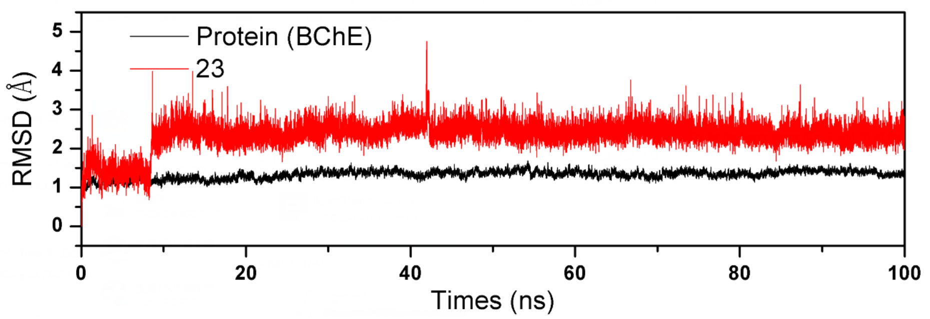


**Figure. S1** Time dependencies of RMSDs for the heavy atoms (Cα, C and N) of the protein and compound **23**.

***Determination of the inhibitory potency on Aβ$_{1–42}$ aggregation***

1,1,1,3,3,3-hexafluoro-2-propanol (HFIP) pre-treated Aβ$_{1–42}$ (GL Biochem Ltd, shanghai, China) was dissolved in DMSO to make a 200 μM stock solution. The stock solution was centrifuged at the speed of 13,500 rpm for 10 min. The above supernatant was used for further experiments. The compounds for testing were dissolved in DMSO at concentrations of 0.8 mM. A screening assay for the compounds to inhibit Aβ aggregation was performed by measuring ThT fluorescence emission. Compounds (2 μL) and 2 μL of 200 μM Aβ$_{1–42}$ were added into 76 μL of phosphate-bufered saline (PBS at pH 7.4) in a 96-well microtiter plate. After incubation for 24 h at room temperature, 80 μL of 5 μM ThT solution (in 50 mM glycine-NaOH at pH 8.5) was added to the reaction solution. Fluorescence emission was measured at 490 nm with an excitation wavelength of 450 nm on a Tecan Spark multimode microplate reader. The fluorescence intensities were compared and the % inhibition was calculated by the following equation: $100 - [(F_i - F_b)/(F_o - F_b) \times 100]$ where $F_i$, $F_o$ and $F_b$ are the fluorescence intensities obtained for Aβ aggregation in the presence of inhibitors, Aβ$_{1–42}$ and ThT; in the presence of Aβ$_{1–42}$ and THT but no inhibitors; and the blanks containing ThT only.

***Cell viability assay***

SH-SY5Y cells were inoculated into 96-well plates, and each well was seeded with $10^4$ cells. After incubation for 24 h, the medium was removed and replaced with 100 μL medium containing the different concentrations of tested compounds for 24 h, and then incubated with 10 μL of 3-(4,5-dimethylthiazol-2-yl)-2,5-diphenyltetrazolium bromide (MTT) at 37 °C for 3 h. In this step, 100 μL DMSO was added to dissolve the

formazan crystals. The formazan dye product was measured by the absorbance at 490 nm on a Tecan Spark multimode microplate reader (Tecan, Switzerland).

***Neuroprotective activity assay***

SH-SY5Y cells were maintained at 37 °C in a humidified atmosphere containing 5% $CO_2$. Cells were seeded into multi-well plates at a density of 2~2.5 × $10^5$ cells/mL in DMEM medium, supplemented with 10% heat-inactivated bovine calf serum (Gibco, South America Origin). Experiments were carried out in 24 h after cells were seeded. $A\beta_{1–42}$ were stored at 4 °C until 0.1 mM stock solutions were prepared in phosphate bufer saline (PBS) on the day of application to cultures. The compounds were first dissolved with DMSO and then diluted with DMEM medium. After pretreatment with the compounds for 2 h, $A\beta_{1–42}$ were added to SH-SY5Y cell cultures in 24 h. Assays for cell viability were performed in 24 h after cultured in fresh medium.

**Figure 1.** $^{1}$H NMR spectrum of **1**

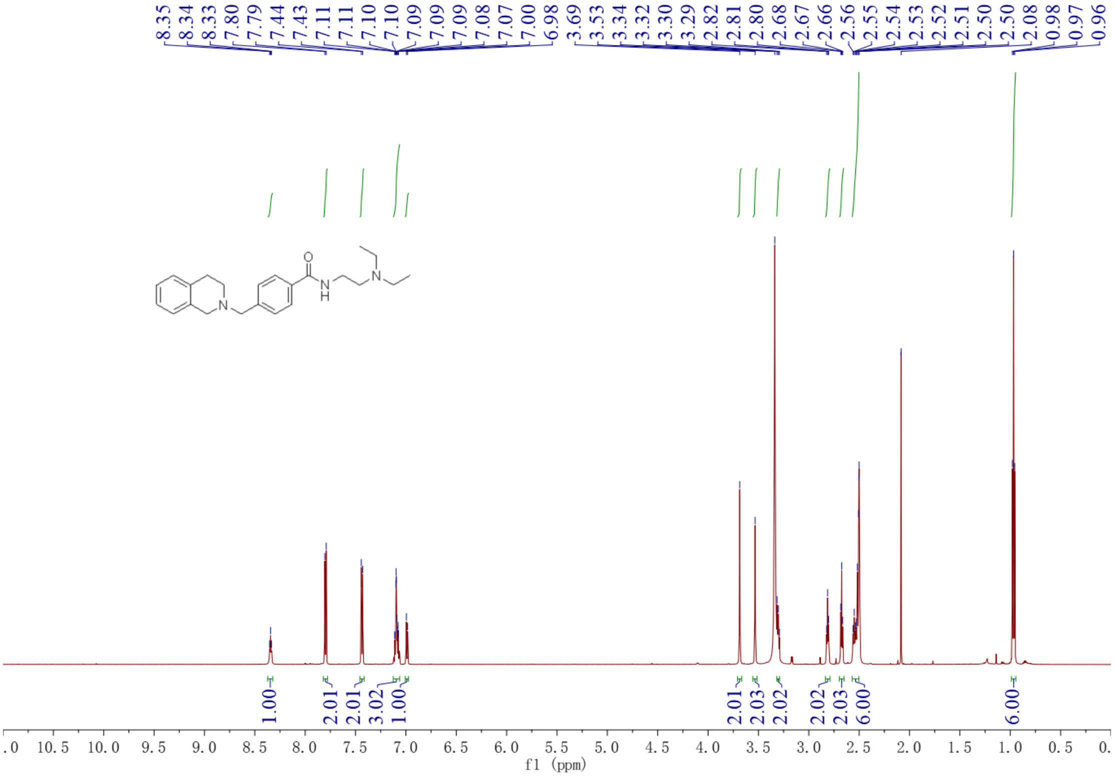


**Figure 2.** $^{13}$C NMR spectrum of **1**

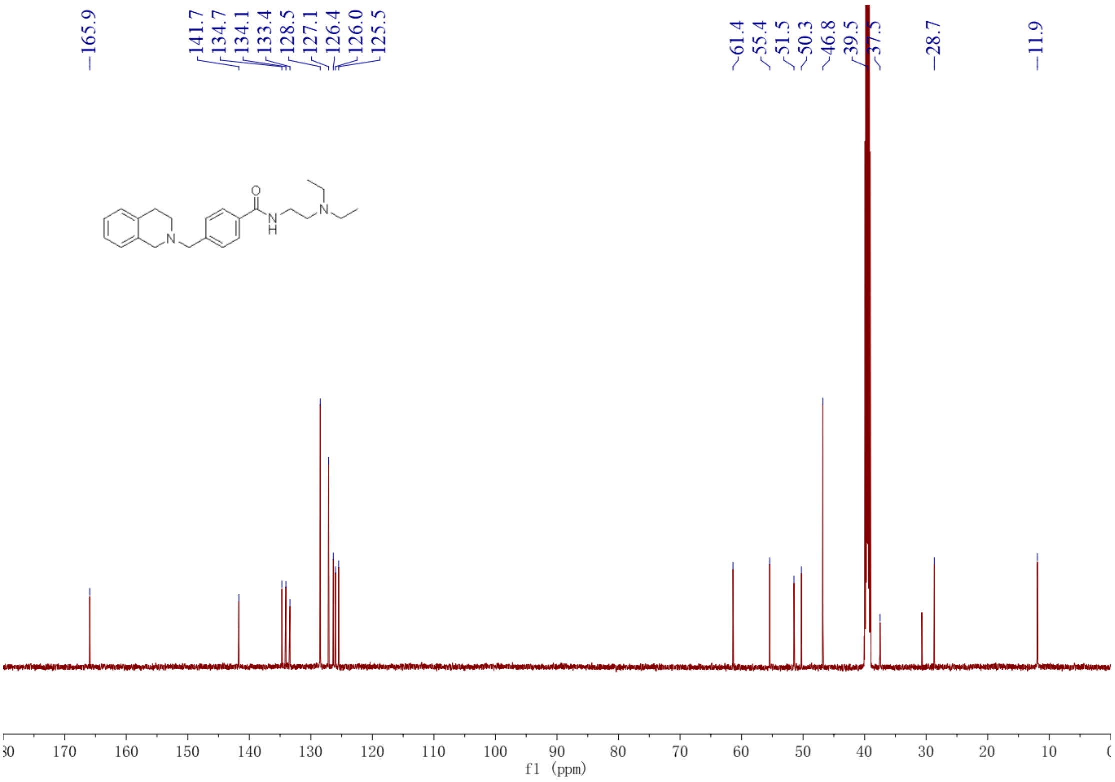

**Figure 3.** HRMS spectrum of **1**

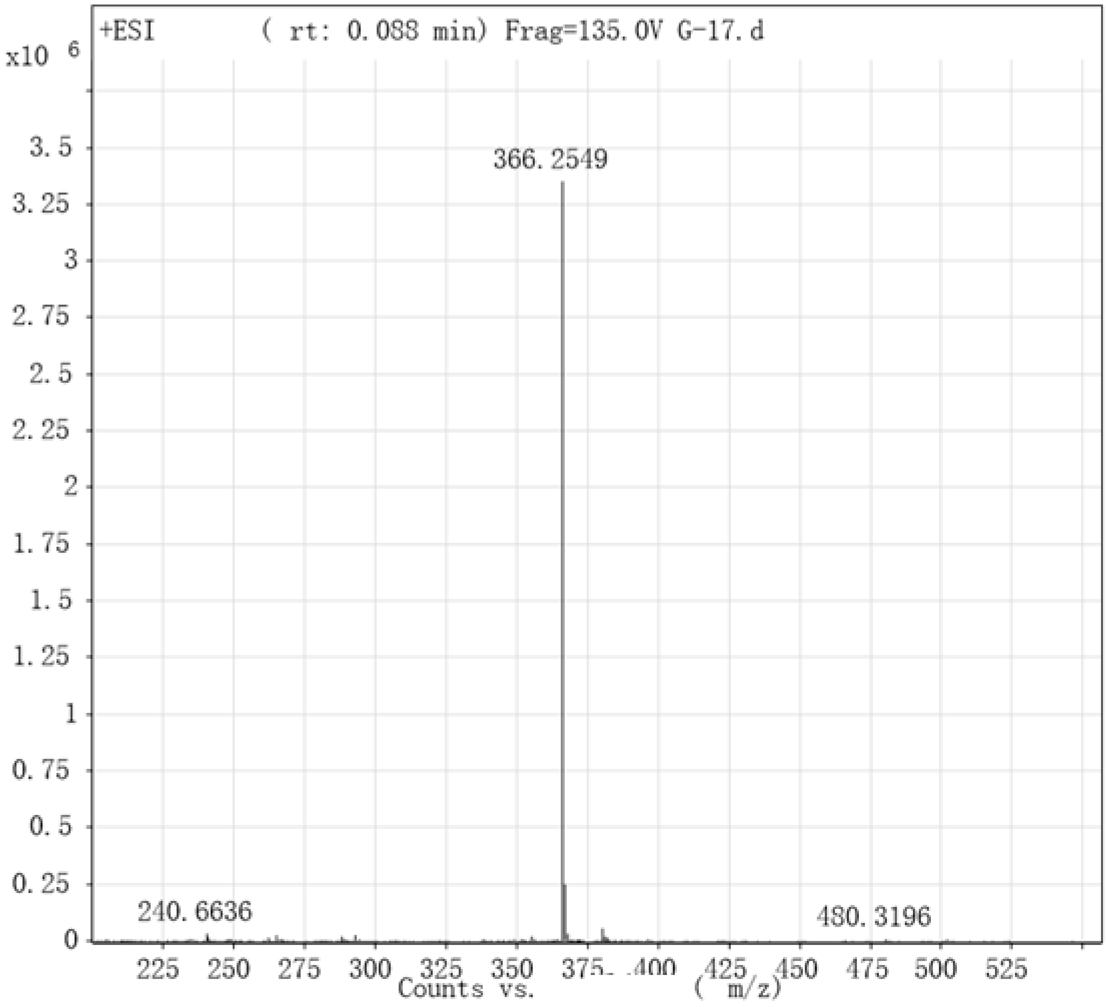

**Figure 4.** $^{1}$H NMR spectrum of **2**

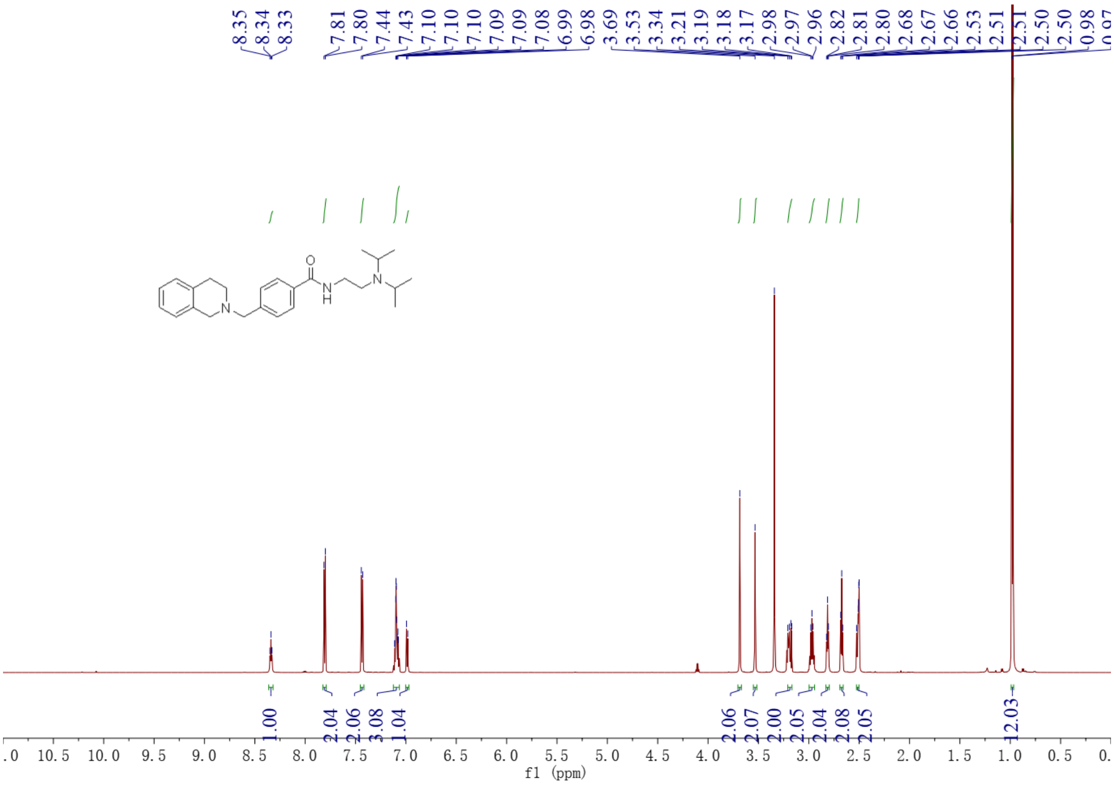


**Figure 5.** $^{13}$C NMR spectrum of **2**

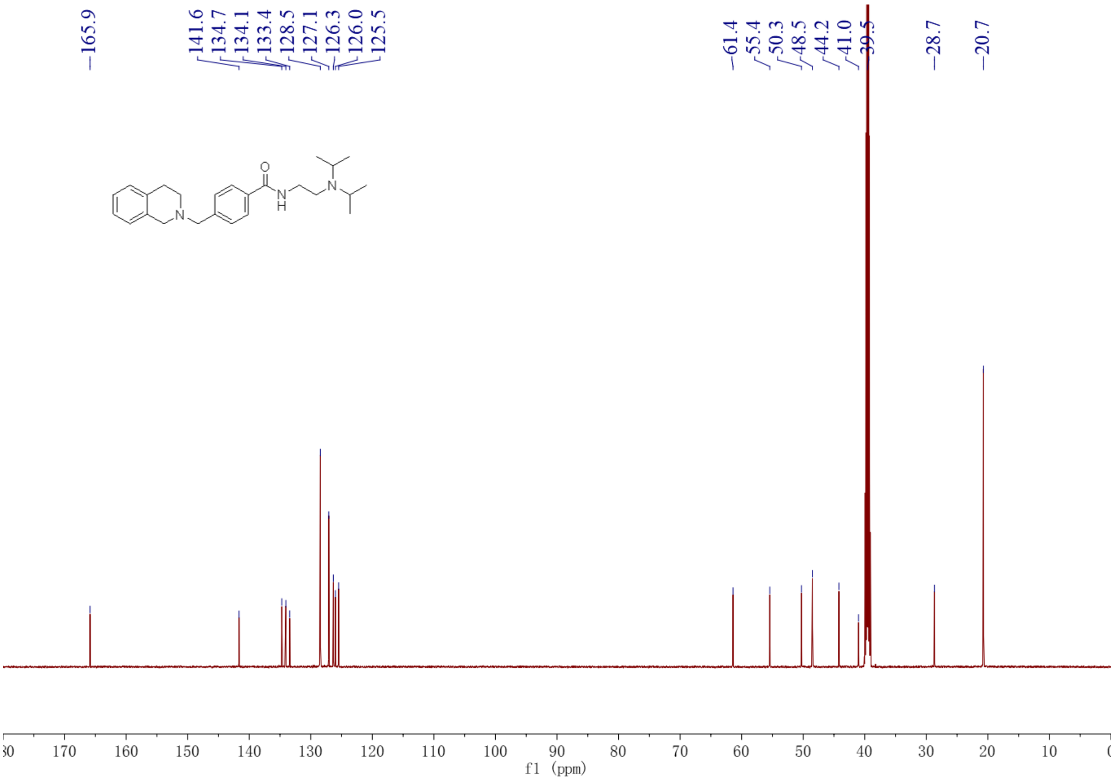

**Figure 6.** HRMS spectrum of **2**

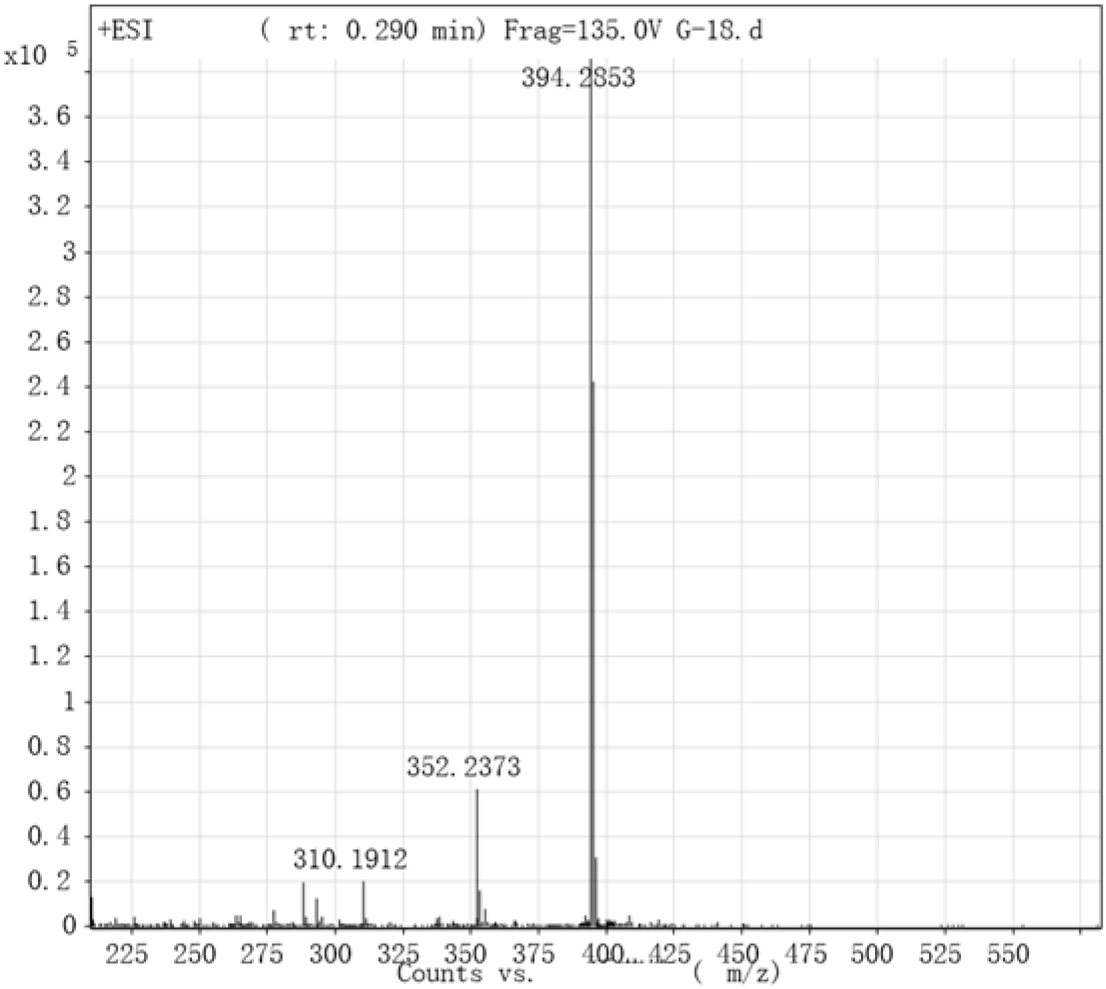

**Figure 7.** $^{1}$H NMR spectrum of **3**

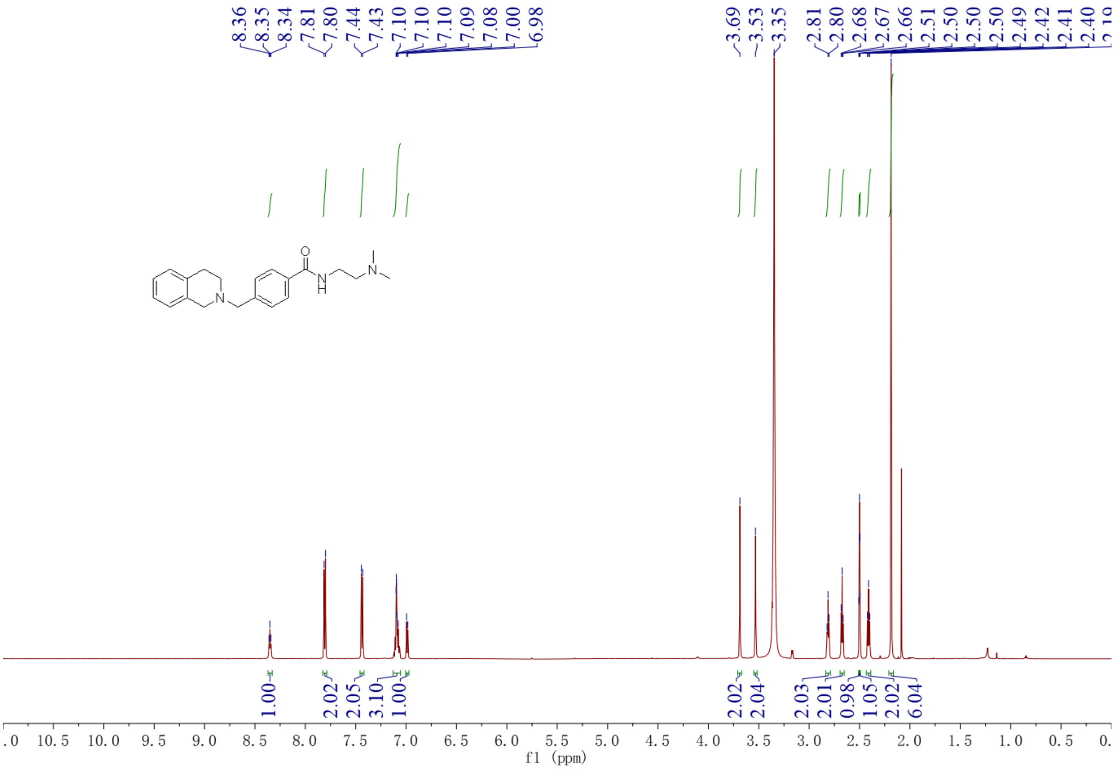


**Figure 8.** $^{13}$C NMR spectrum of **3**

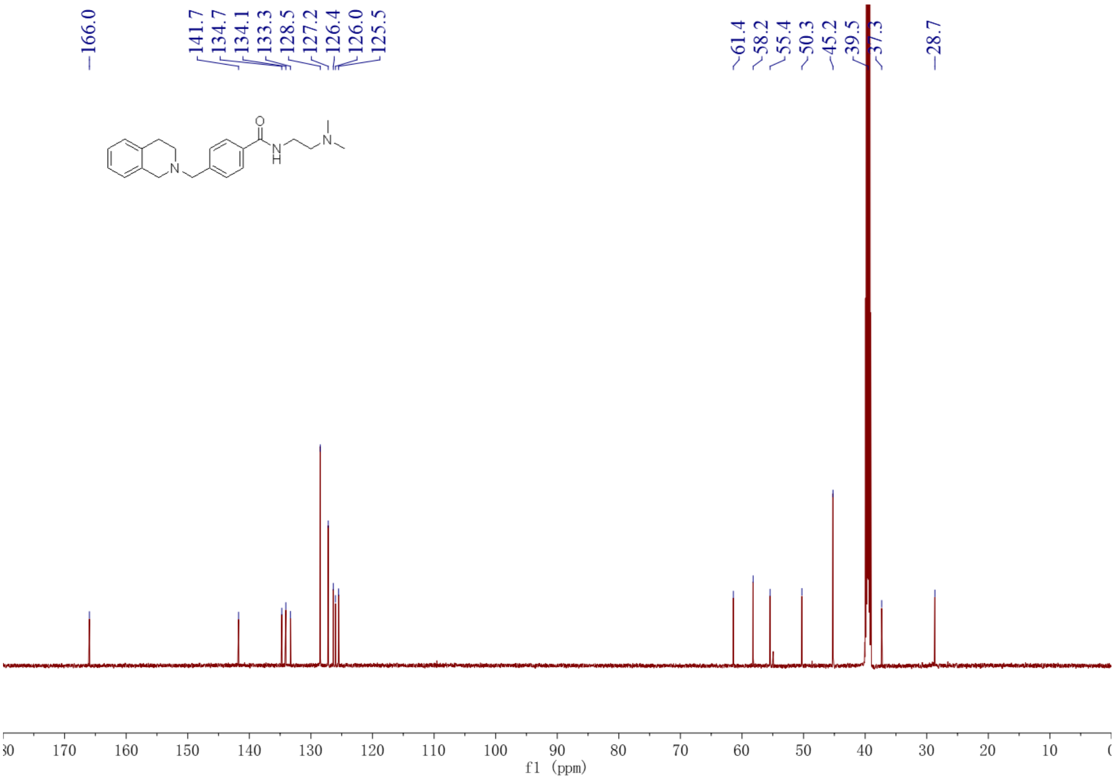

**Figure 9.** HRMS spectrum of **3**

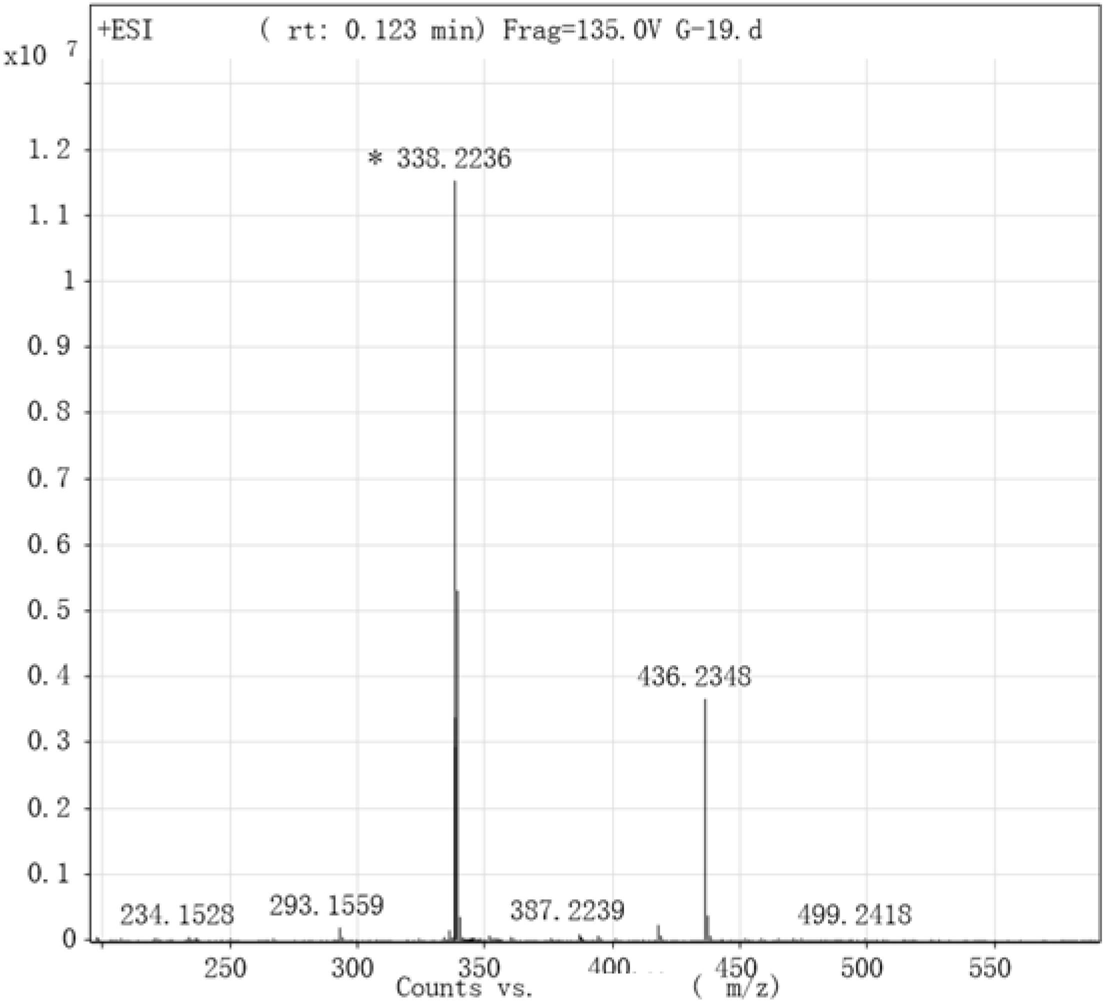

**Figure 10.** $^{1}$H NMR spectrum of **4**

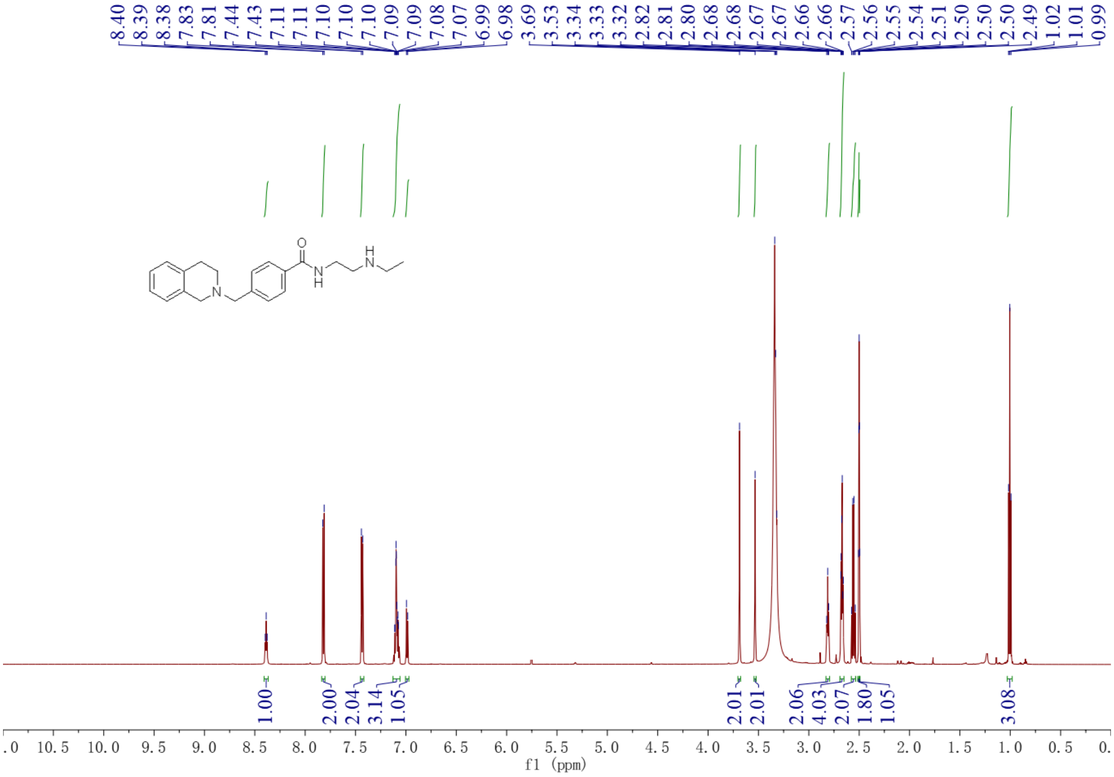


**Figure 11.** $^{13}$C NMR spectrum of **4**

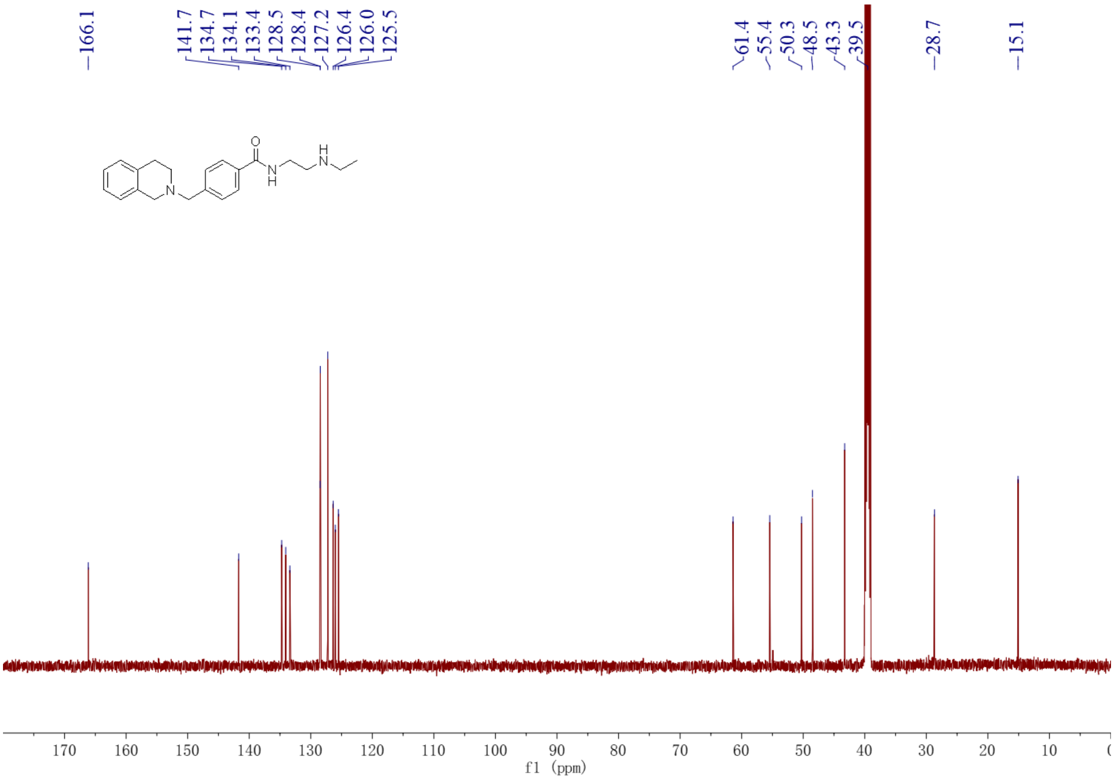

**Figure 12.** HRMS spectrum of **4**

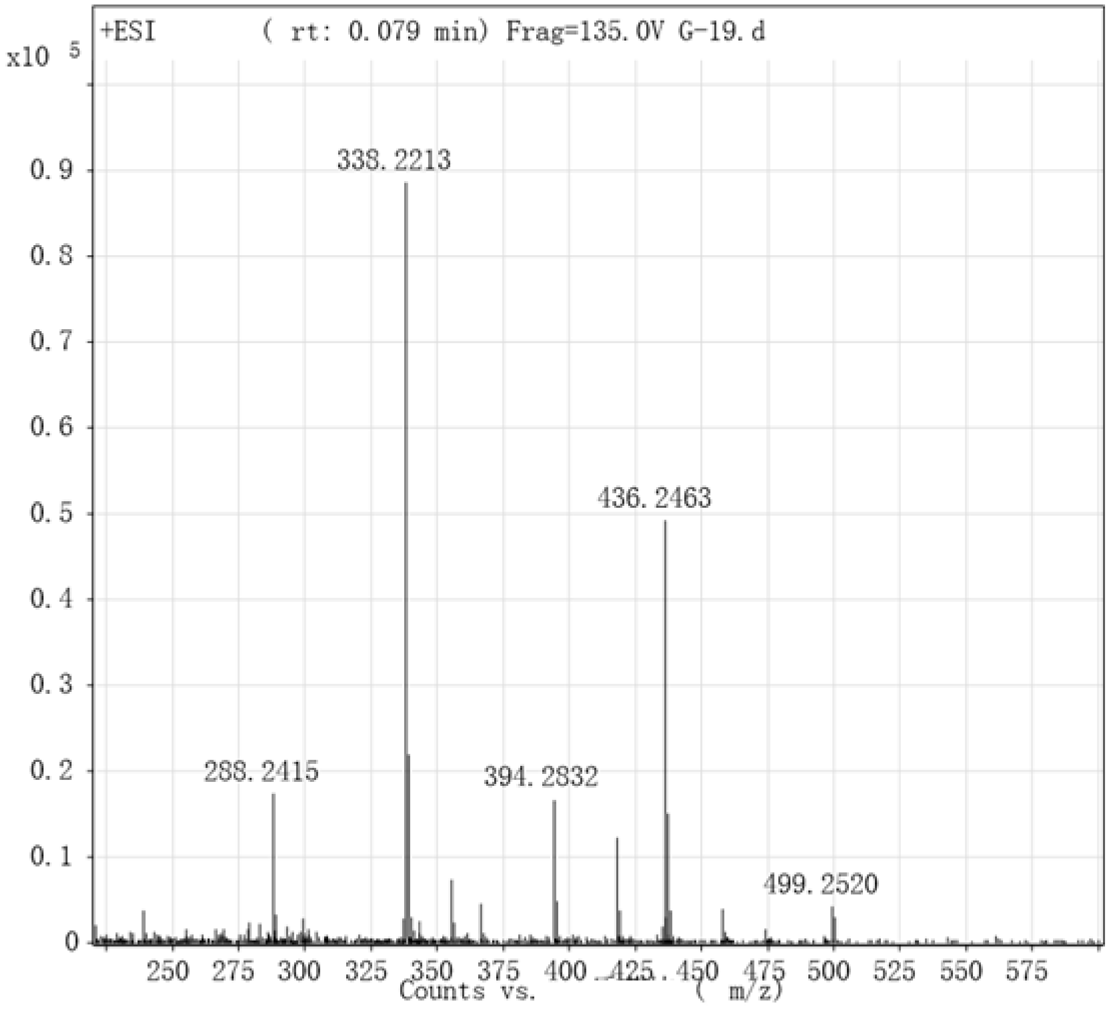

**Figure 13.** $^{1}$H NMR spectrum of **5**

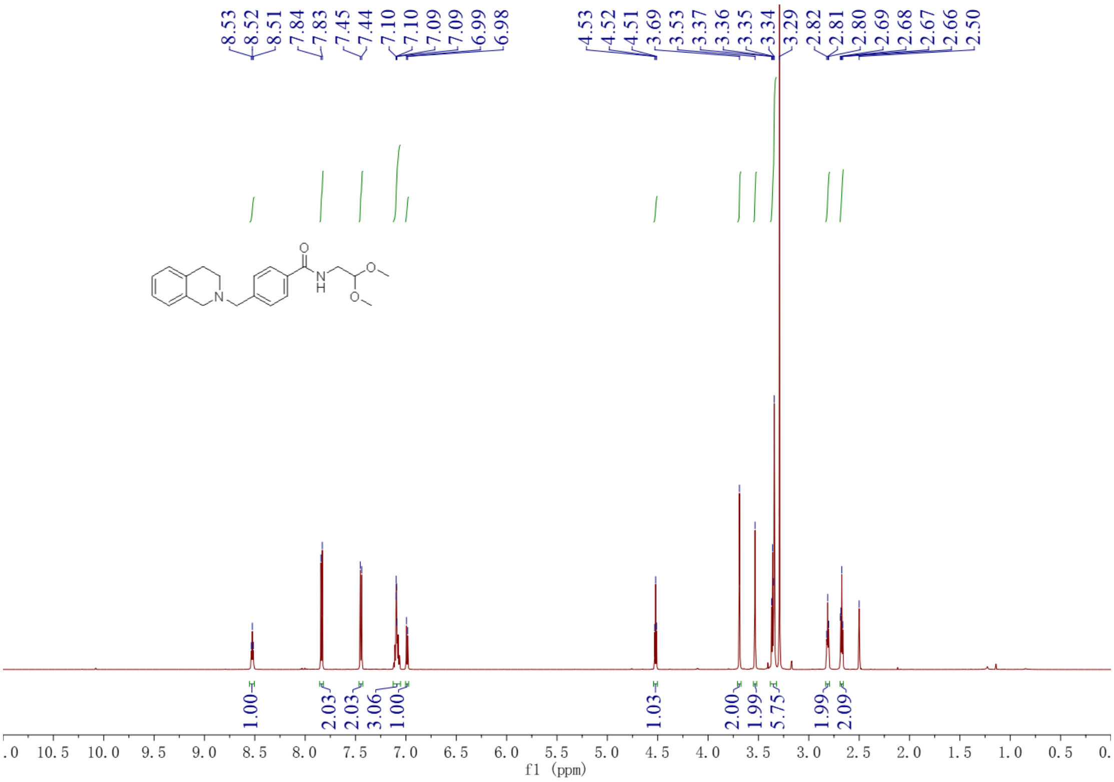

**Figure 14.** $^{13}$C NMR spectrum of **5**

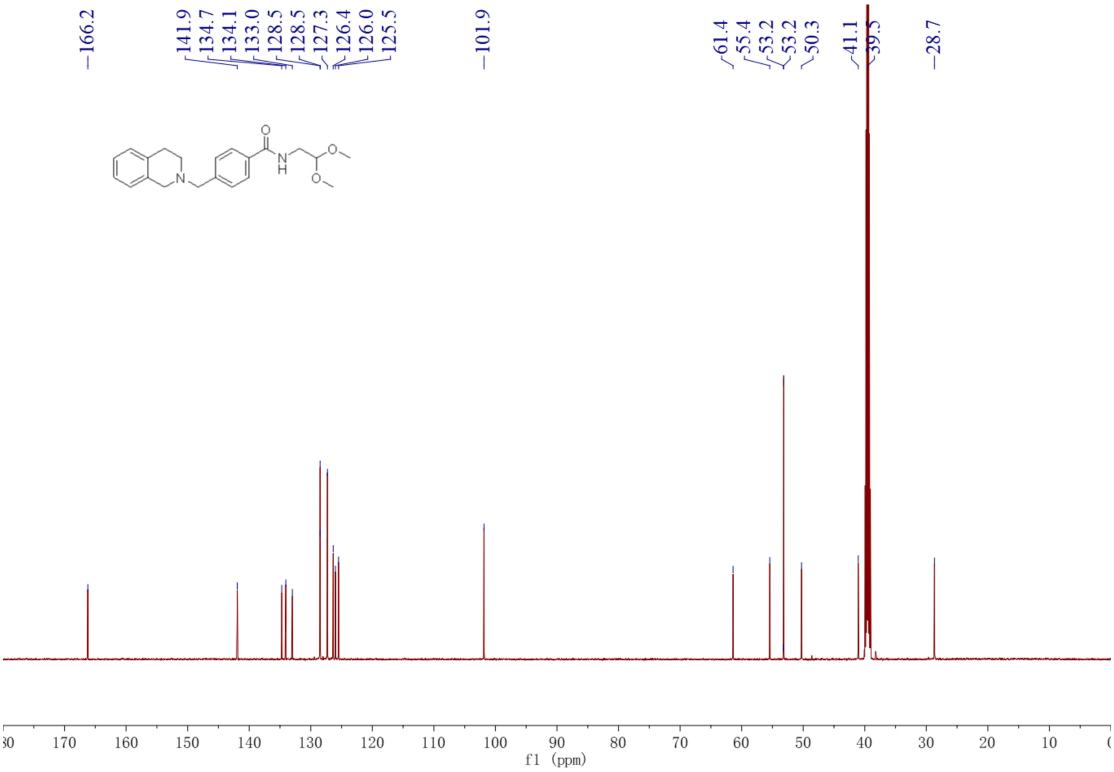

**Figure 15.** HRMS spectrum of **5**

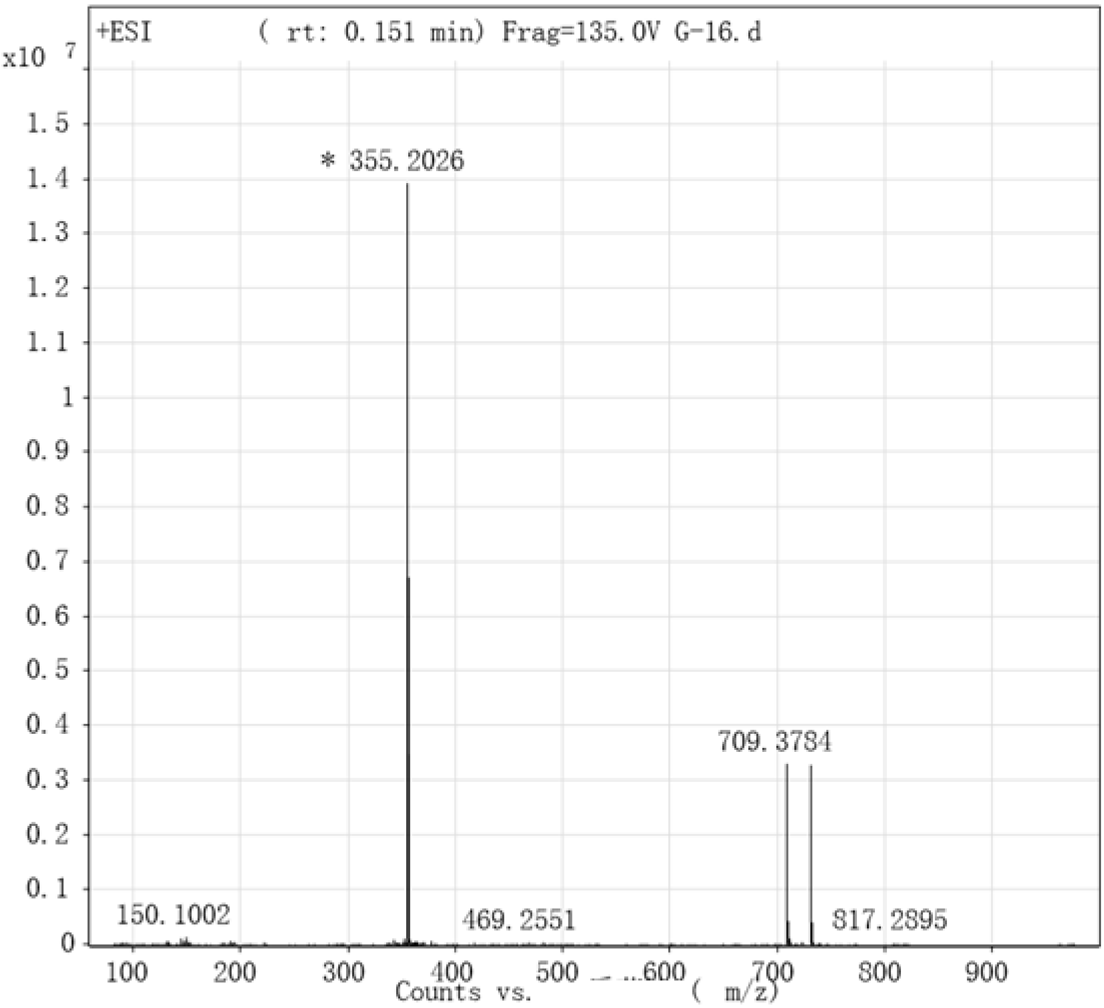

**Figure 16.** $^{1}$H NMR spectrum of **6**

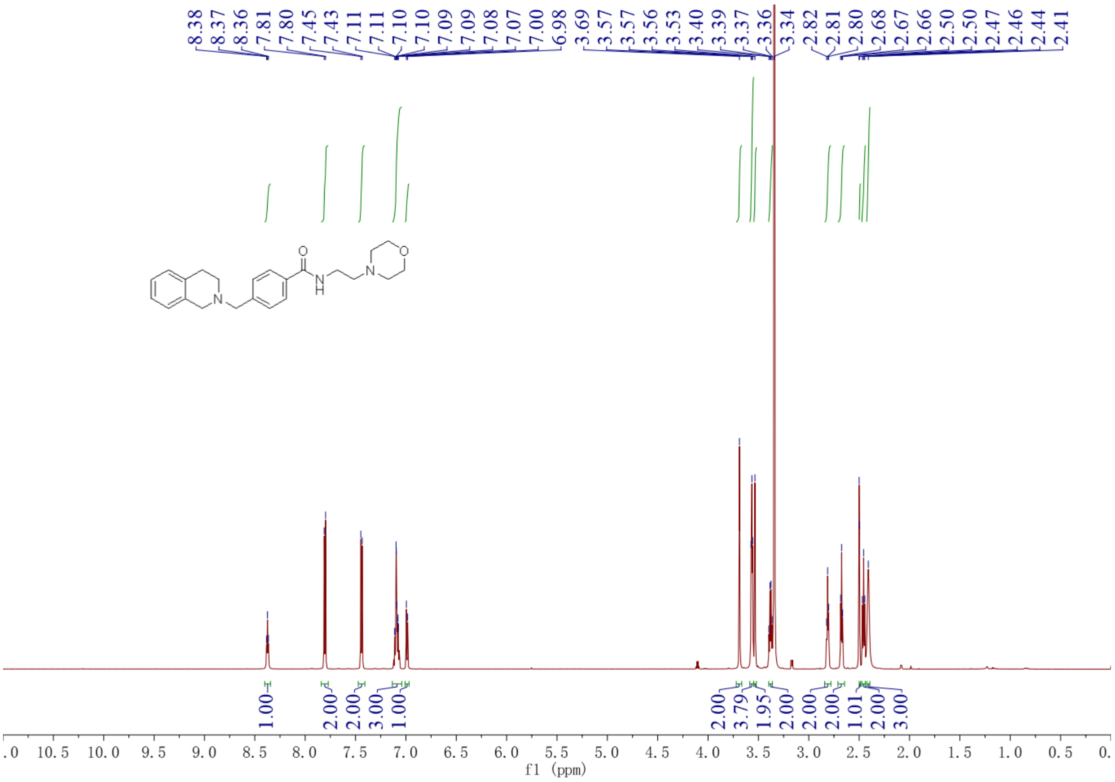


**Figure 17.** $^{13}$C NMR spectrum of **6**

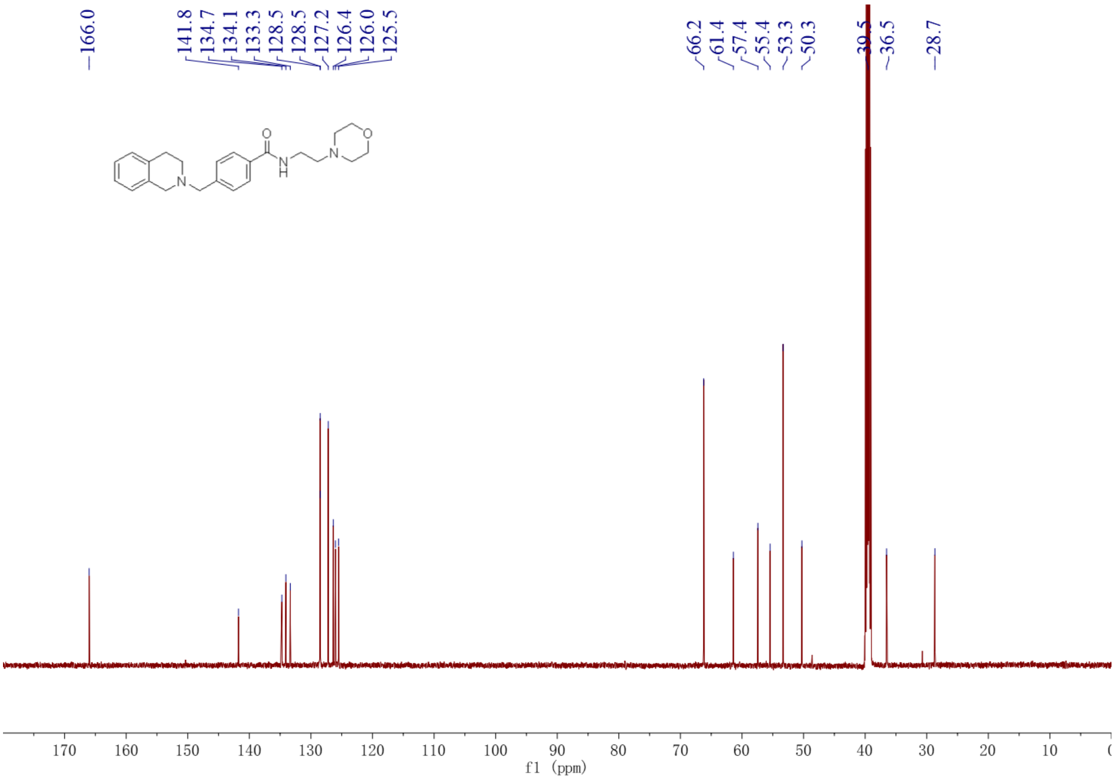

**Figure 18.** HRMS spectrum of **6**

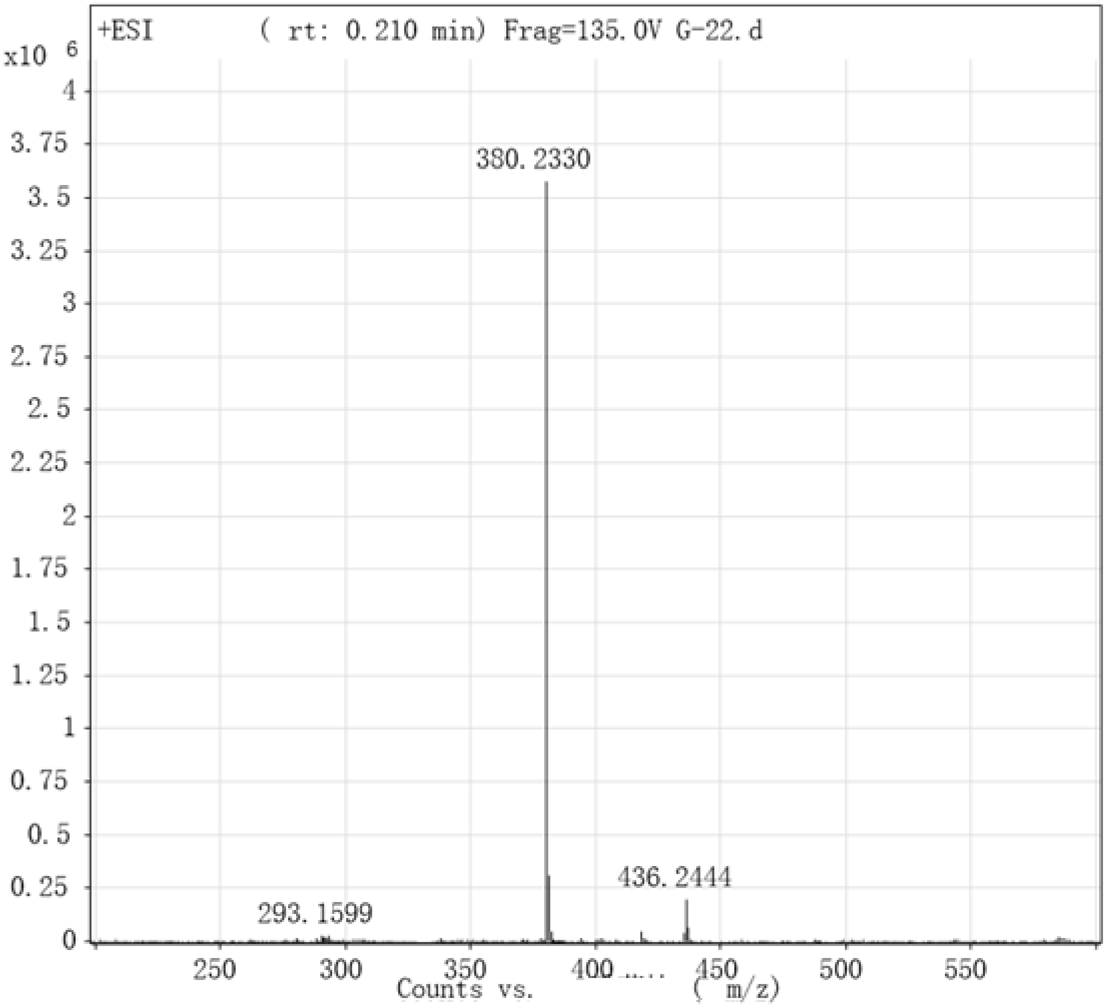

**Figure 19.** $^{1}$H NMR spectrum of **7**

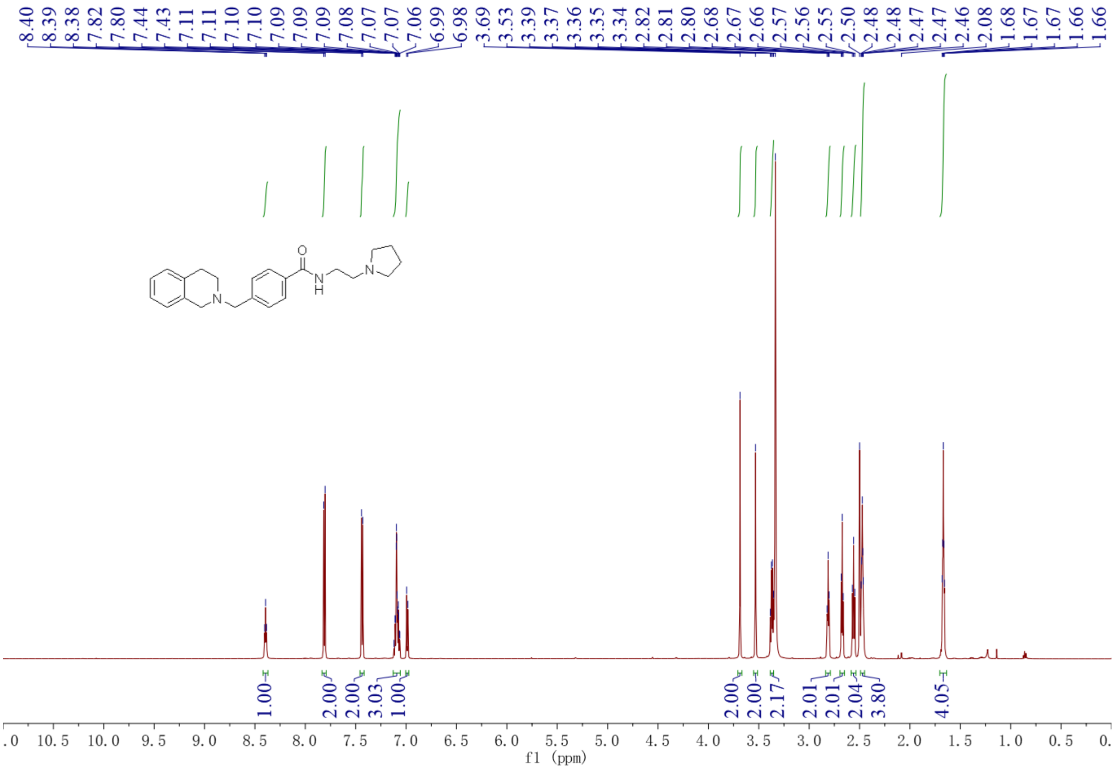

**Figure 20.** $^{13}$C NMR spectrum of **7**

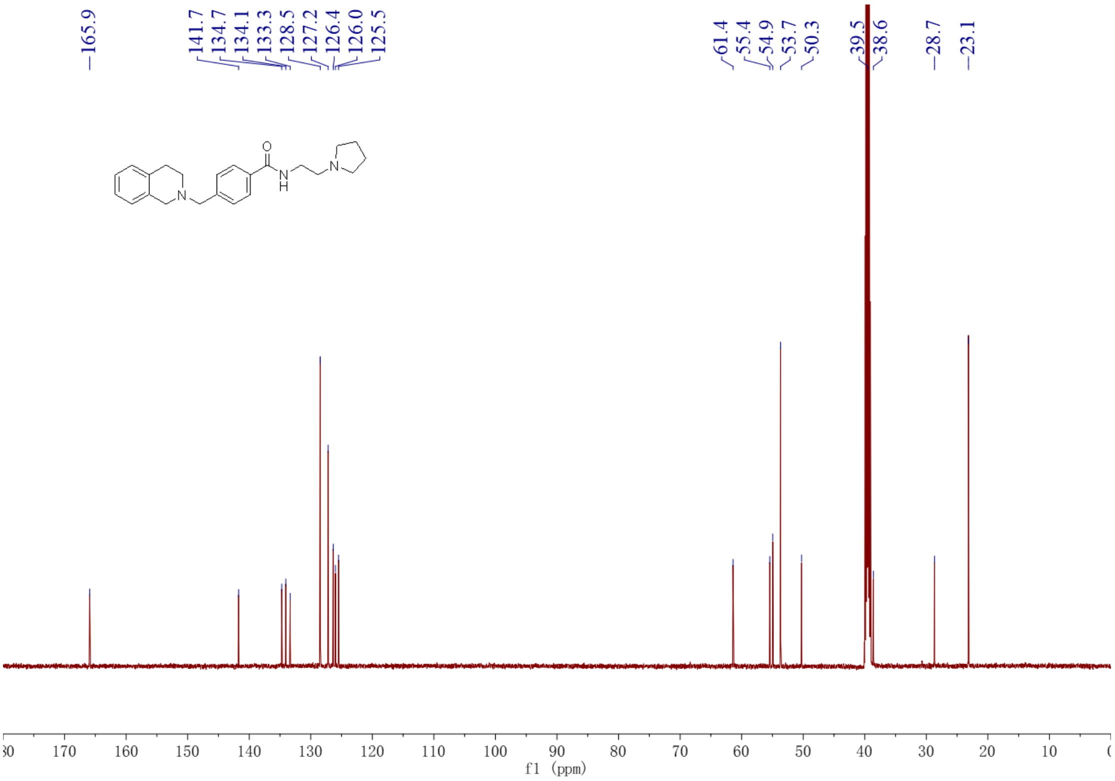

**Figure 21.** HRMS spectrum of **7**

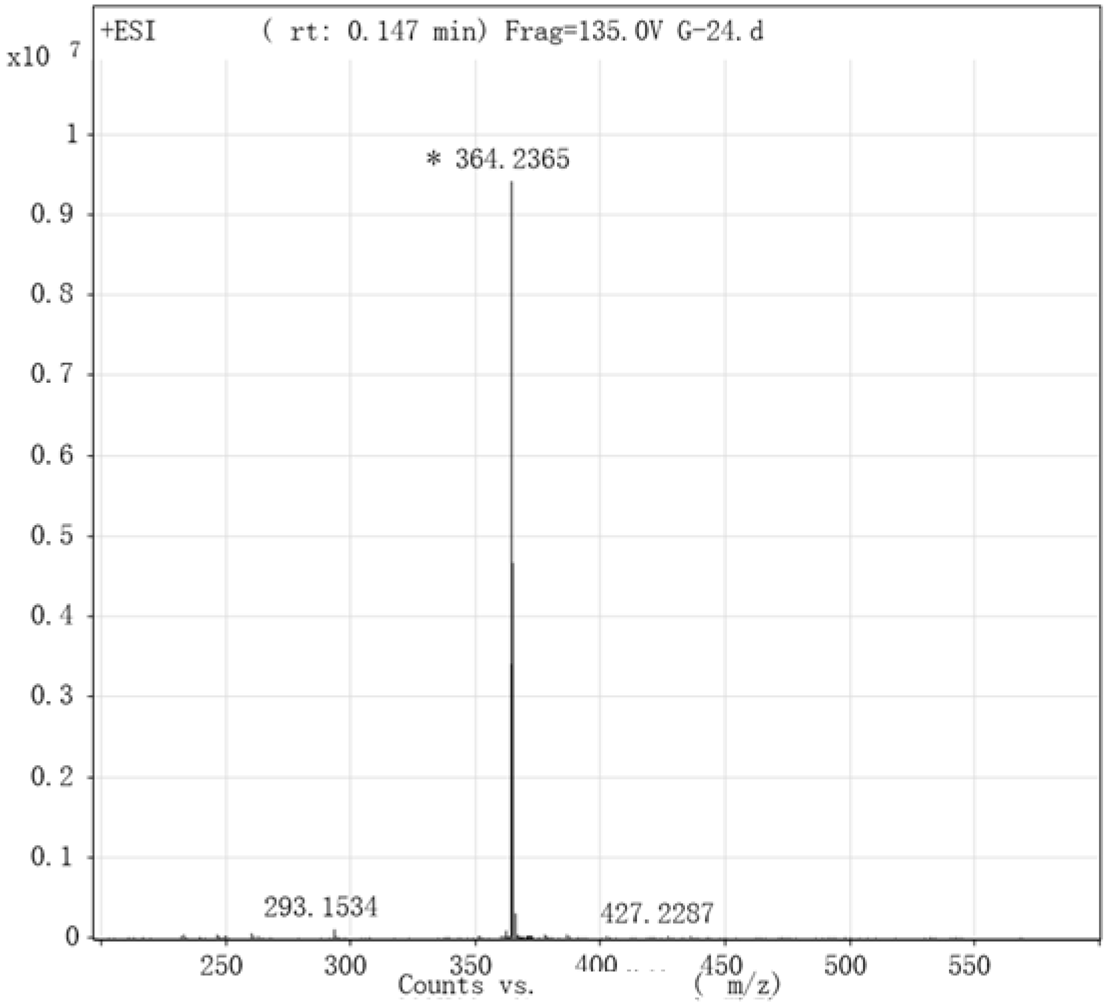

**Figure 22.** $^{1}$H NMR spectrum of **8**

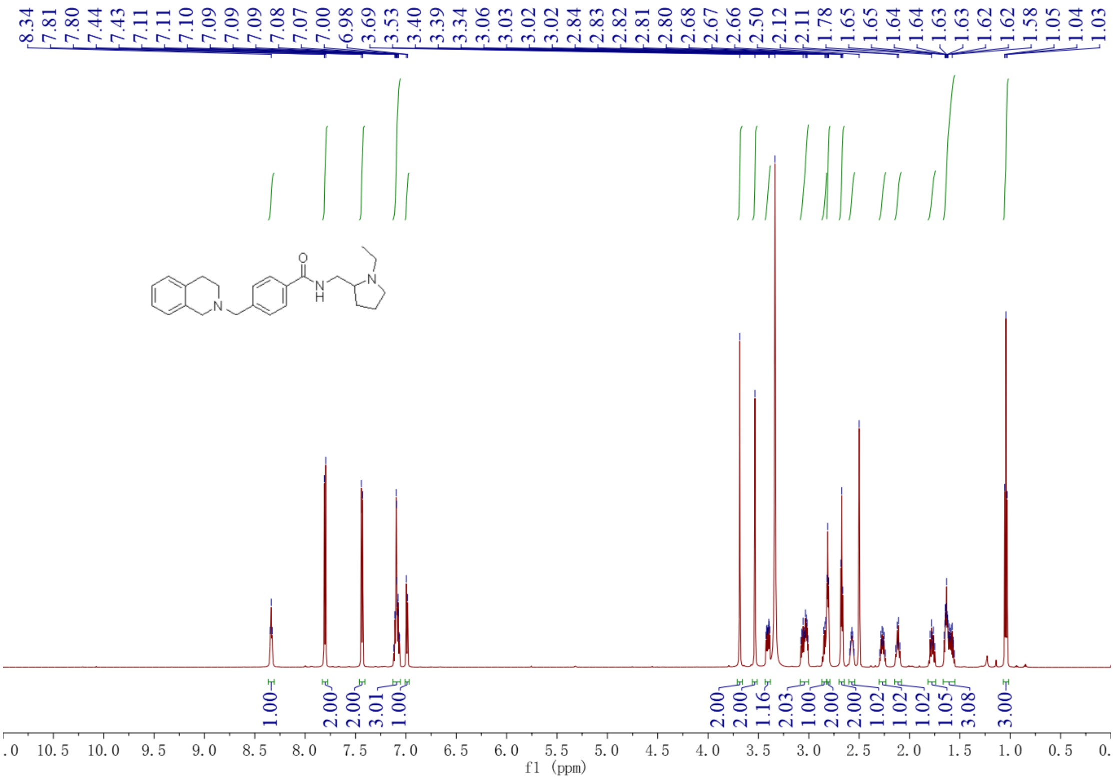

**Figure 23.** $^{13}$C NMR spectrum of **8**

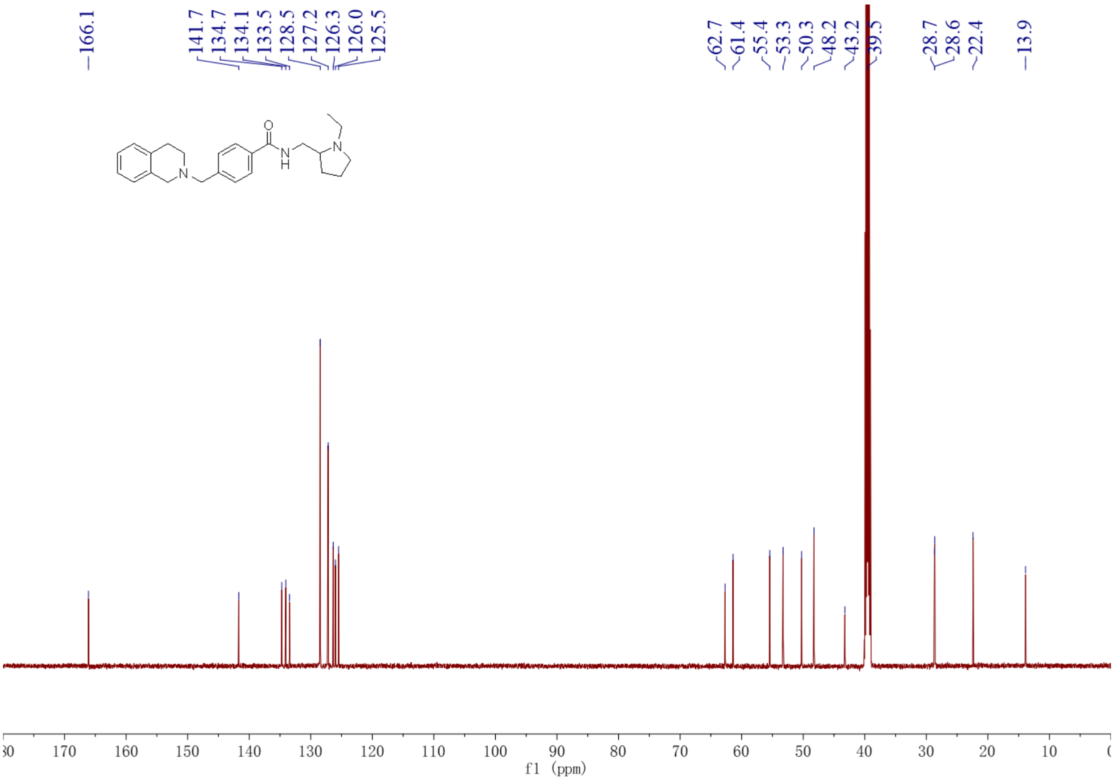

**Figure 24.** HRMS spectrum of **8**

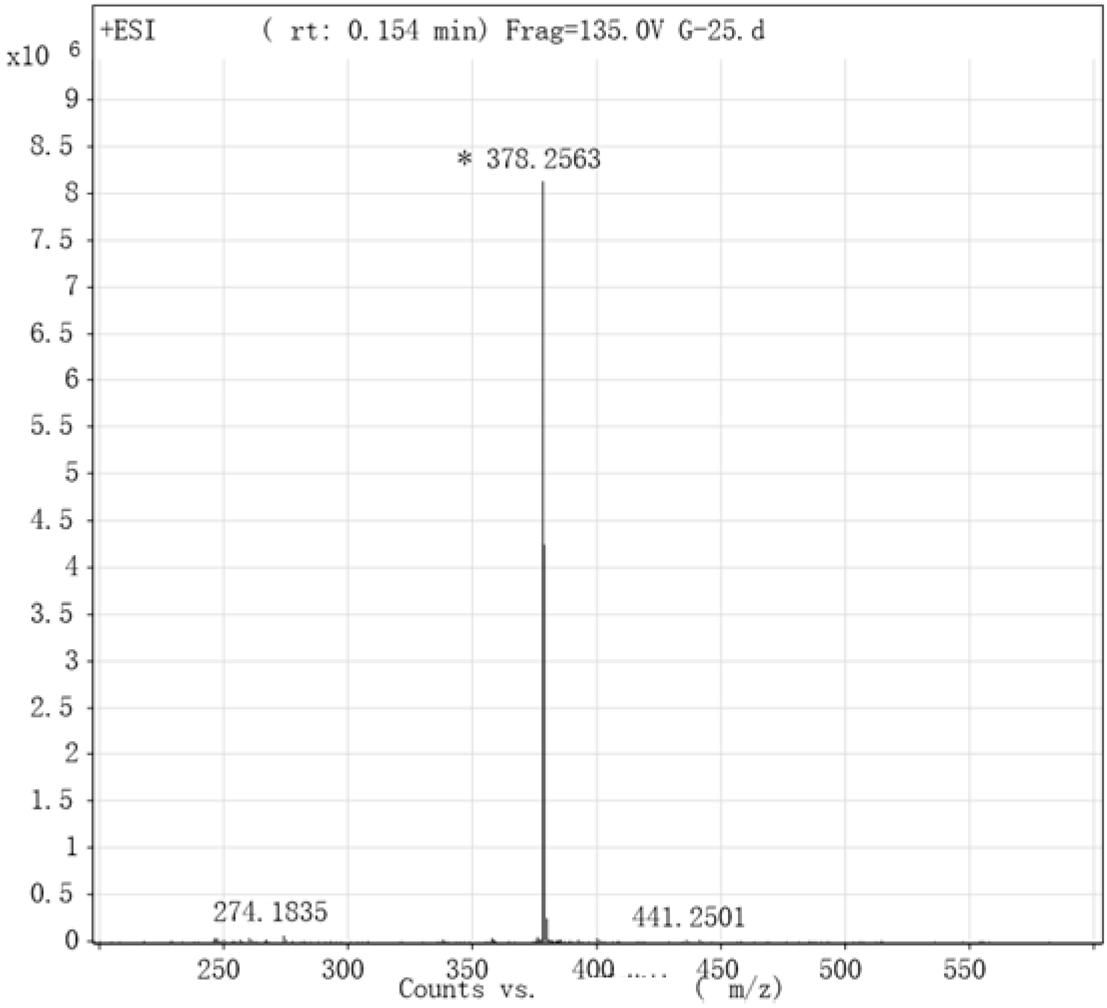

**Figure 25.** $^{1}$H NMR spectrum of **9**

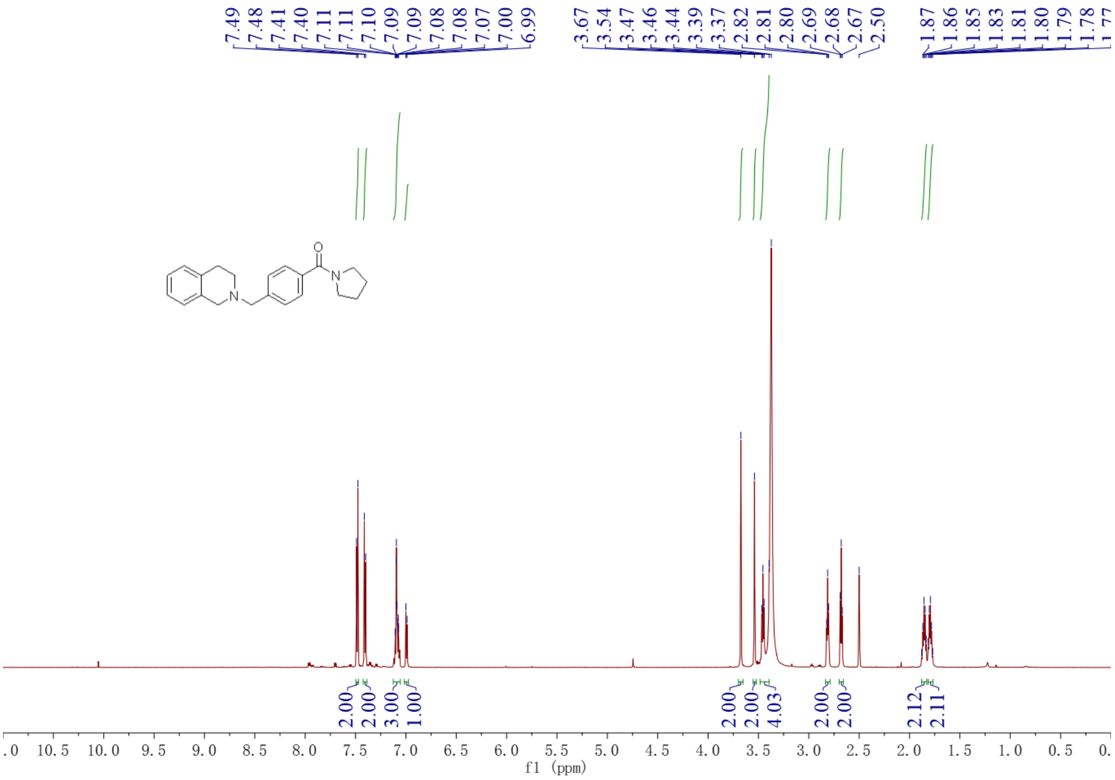

**Figure 26.** $^{13}$C NMR spectrum of **9**

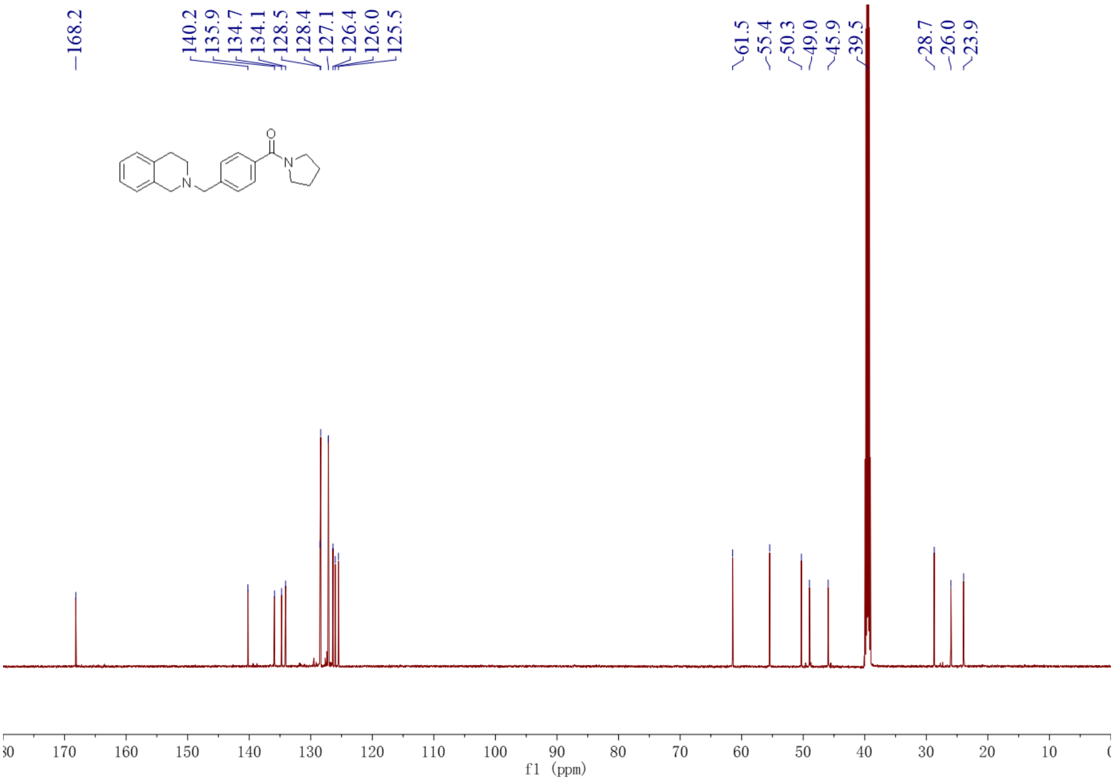

**Figure 27.** HRMS spectrum of **9**

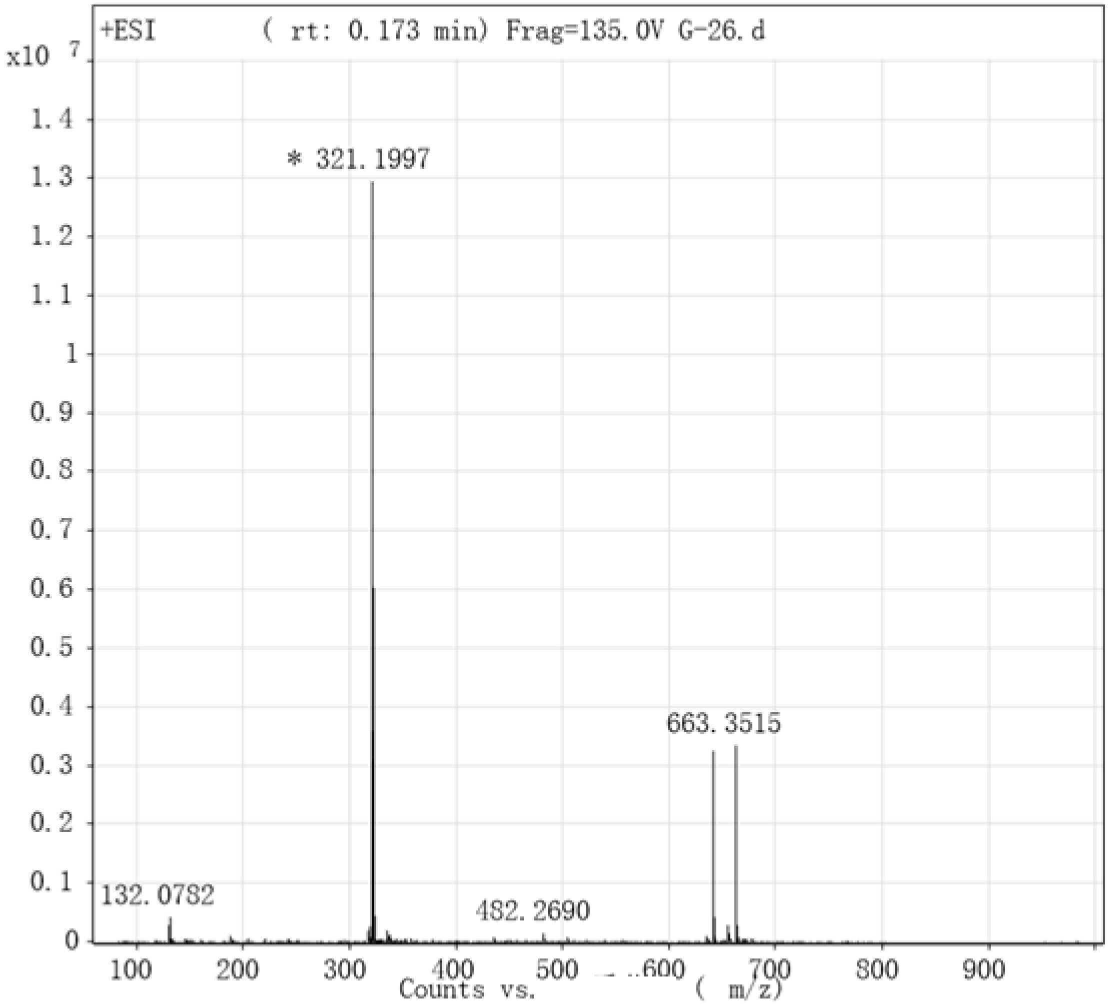

**Figure 28.** $^{1}$H NMR spectrum of **10**

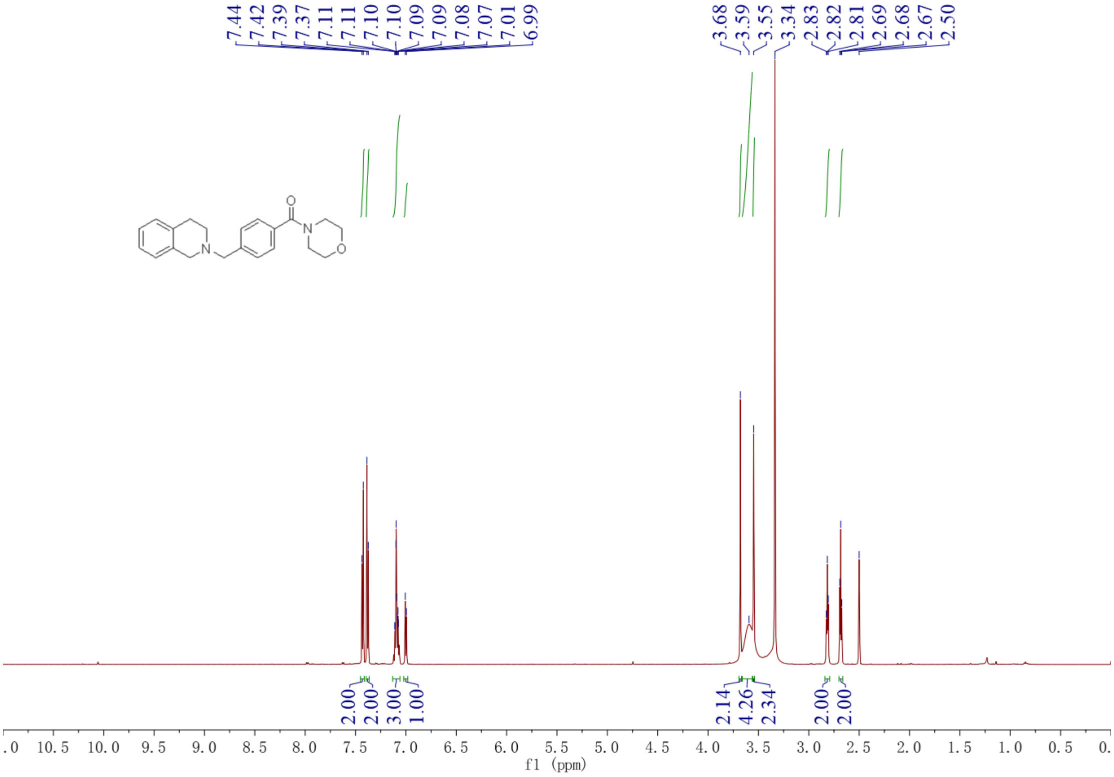


**Figure 29.** $^{13}$C NMR spectrum of **10**

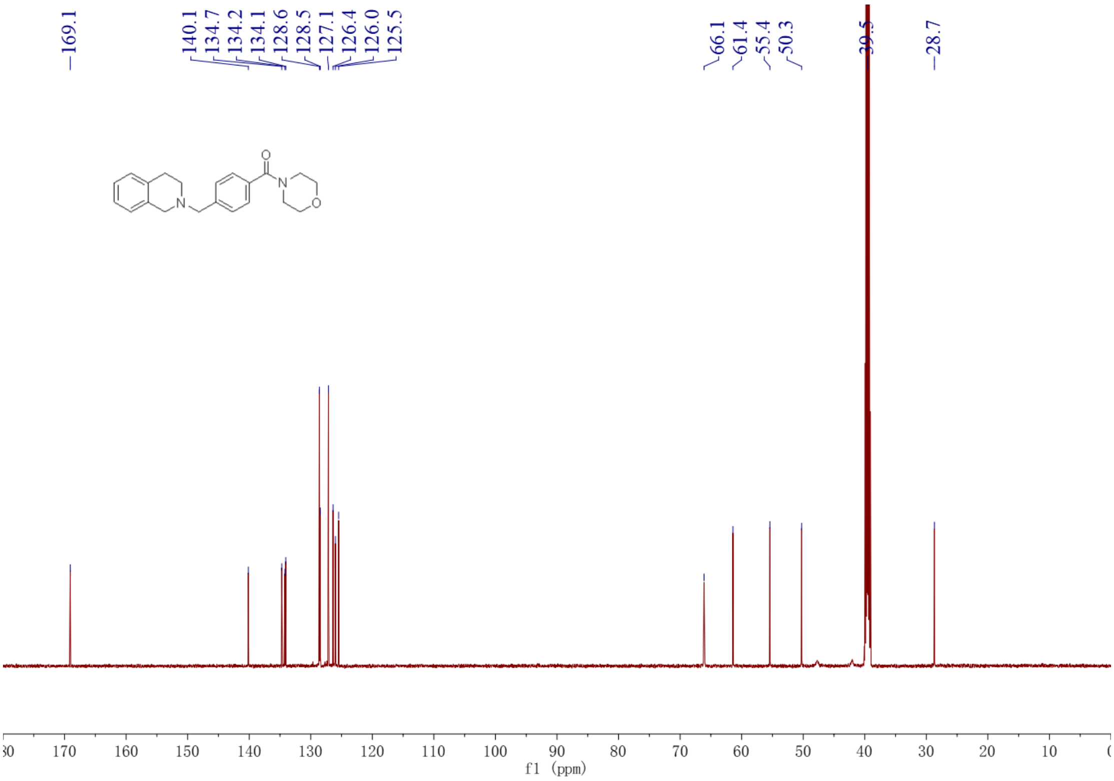

**Figure 30.** HRMS spectrum of **10**

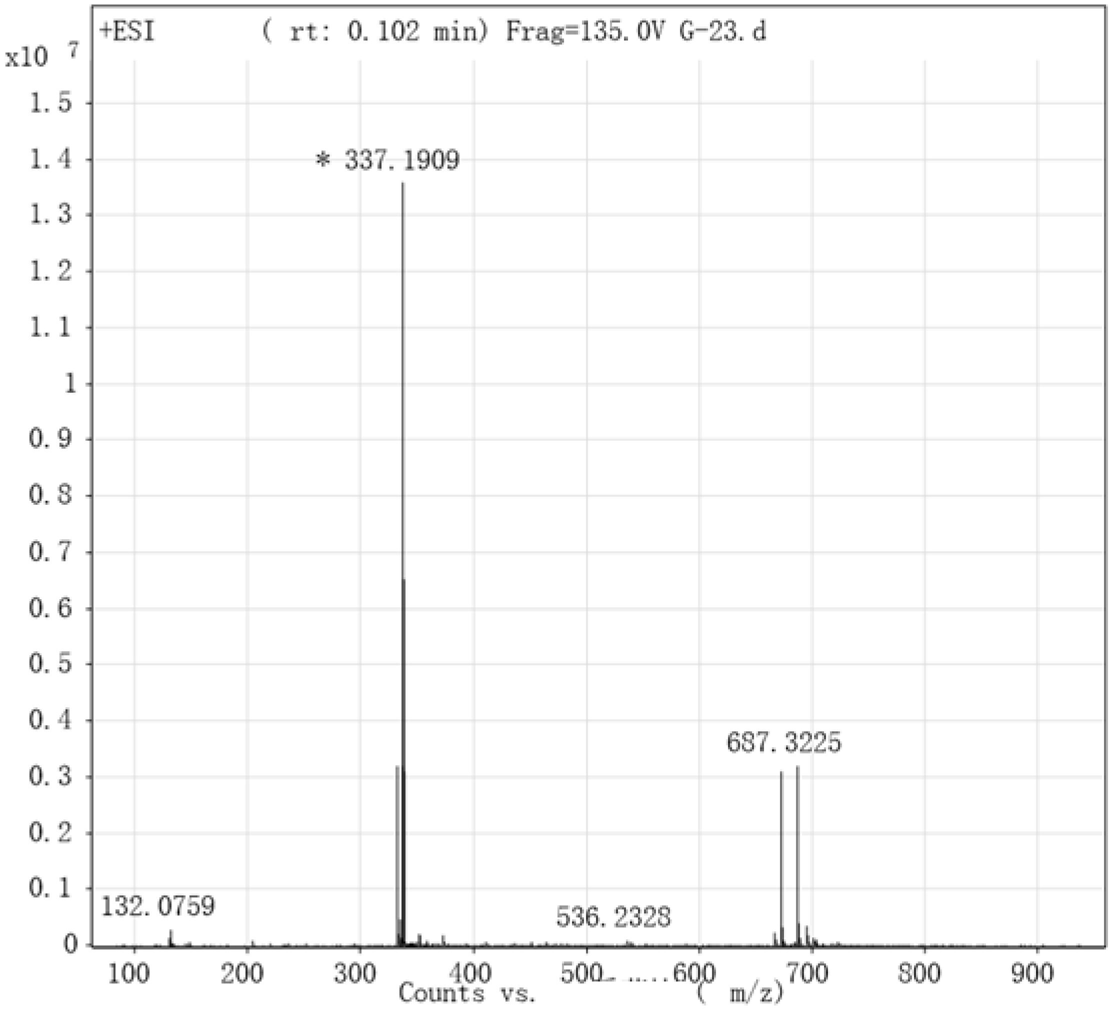

**Figure 31.** $^{1}$H NMR spectrum of **11**

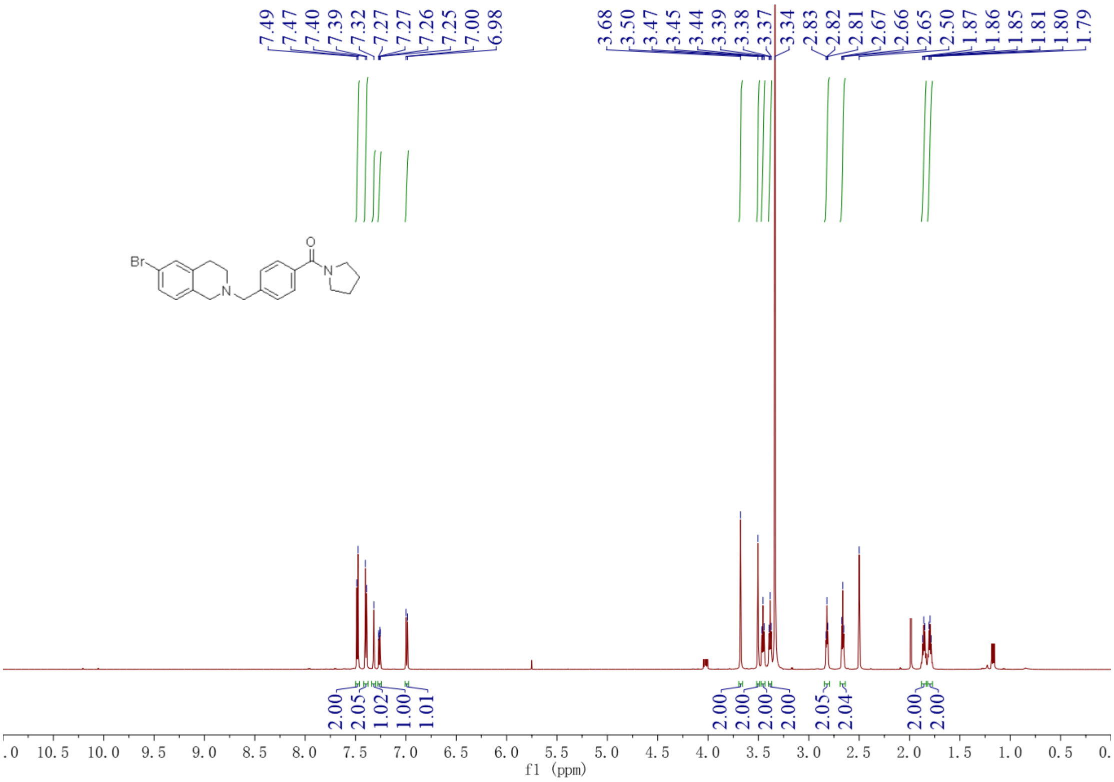


**Figure 32.** $^{13}$C NMR spectrum of **11**

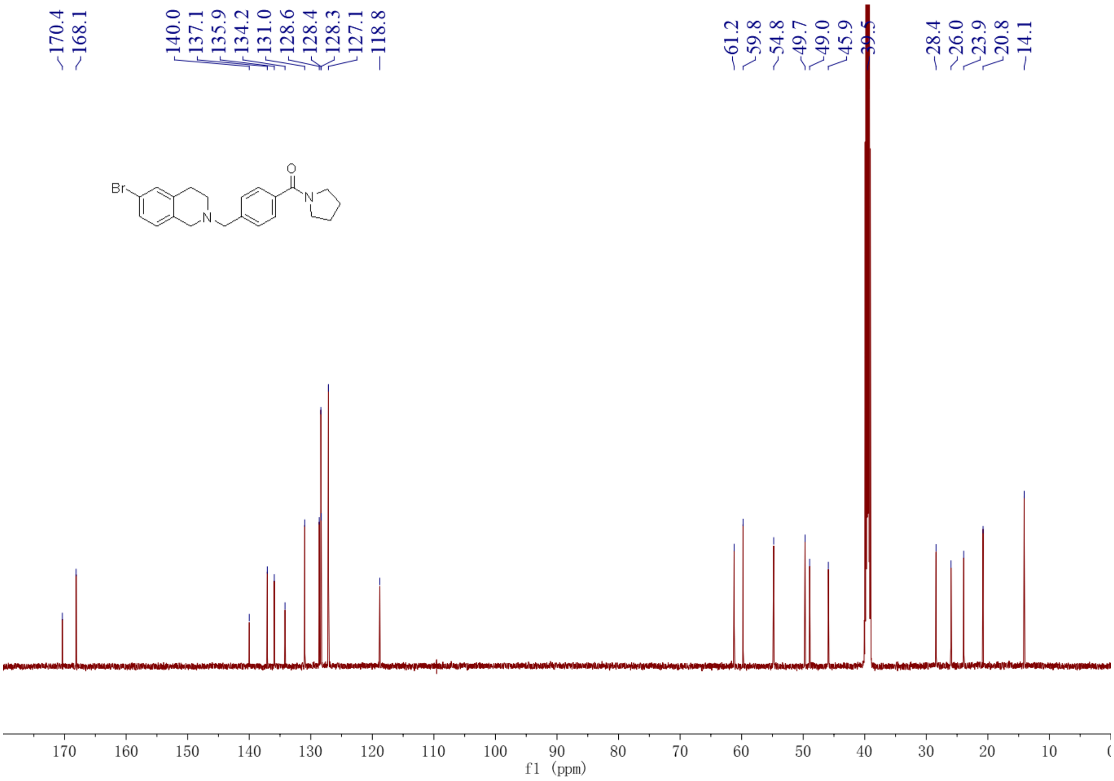

**Figure 33.** HRMS spectrum of **11**

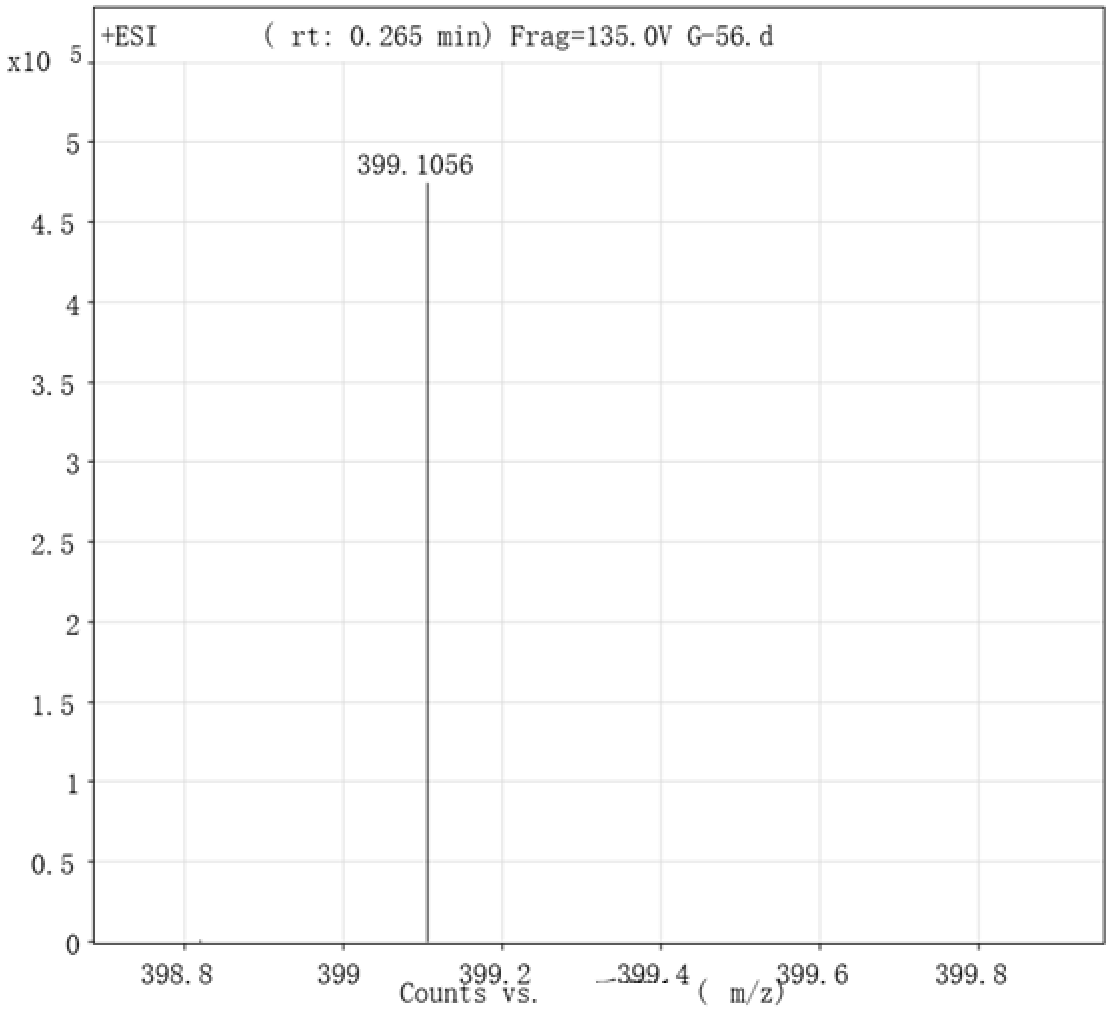

**Figure 34.** $^{1}$H NMR spectrum of **12**

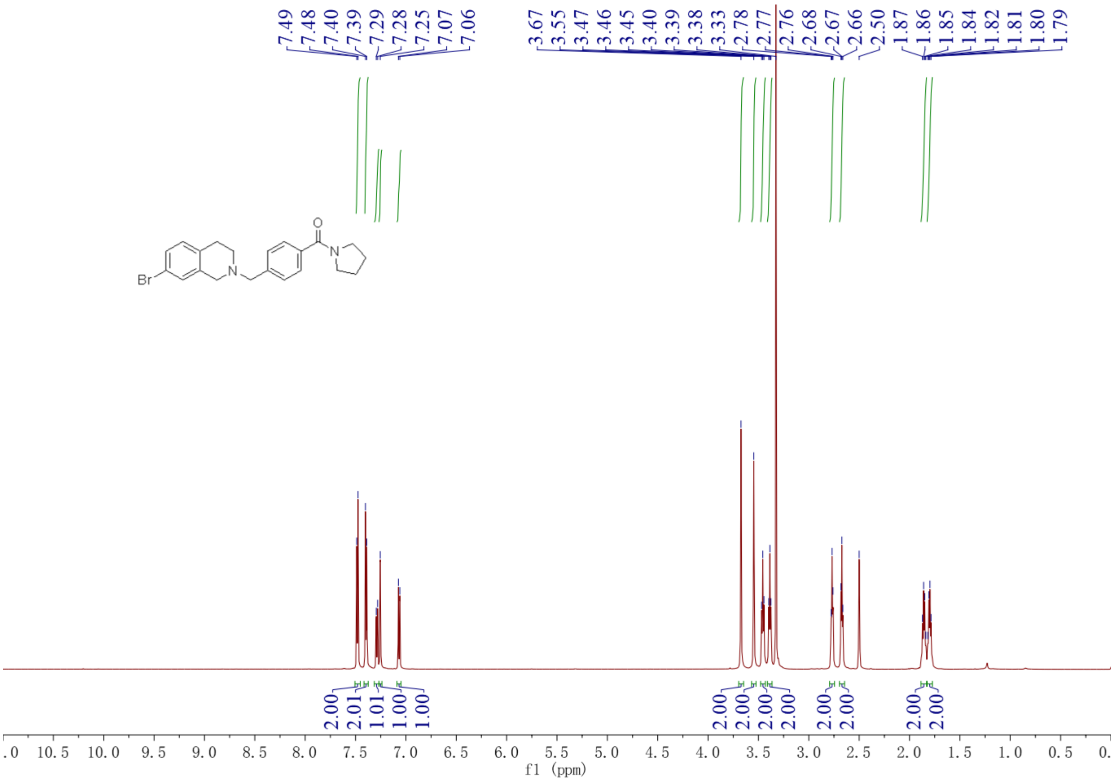


**Figure 36.** $^{13}$C NMR spectrum of **12**

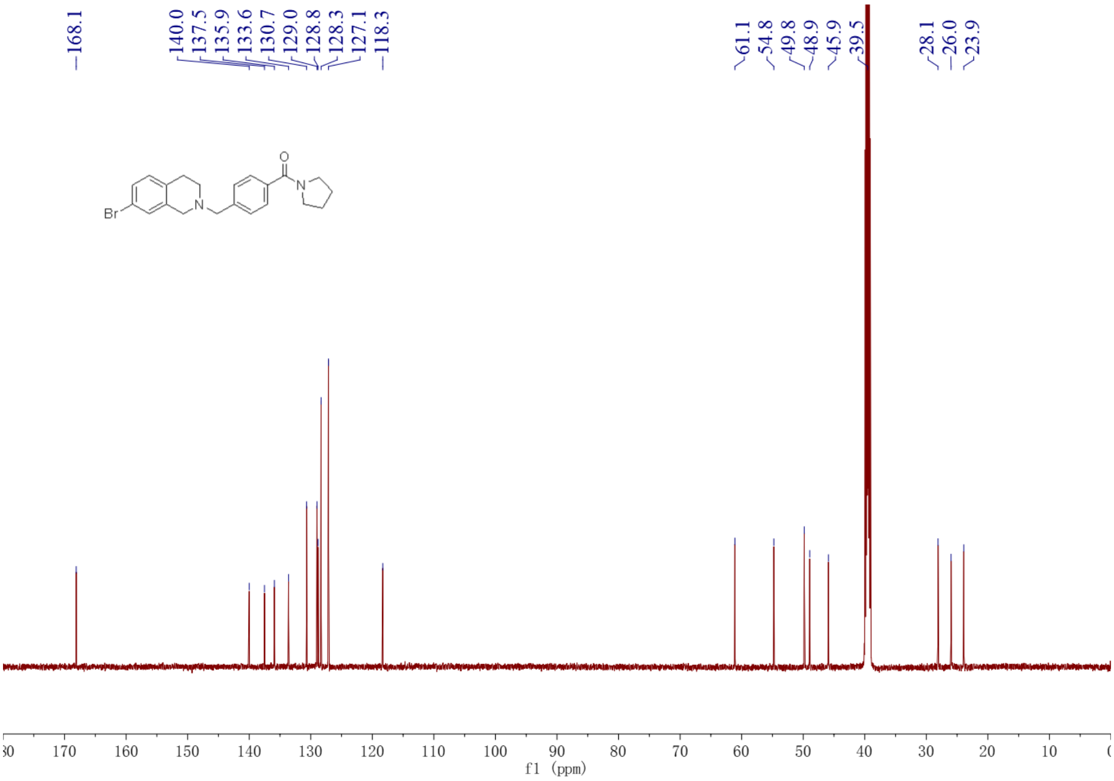

**Figure 36.** HRMS spectrum of **12**

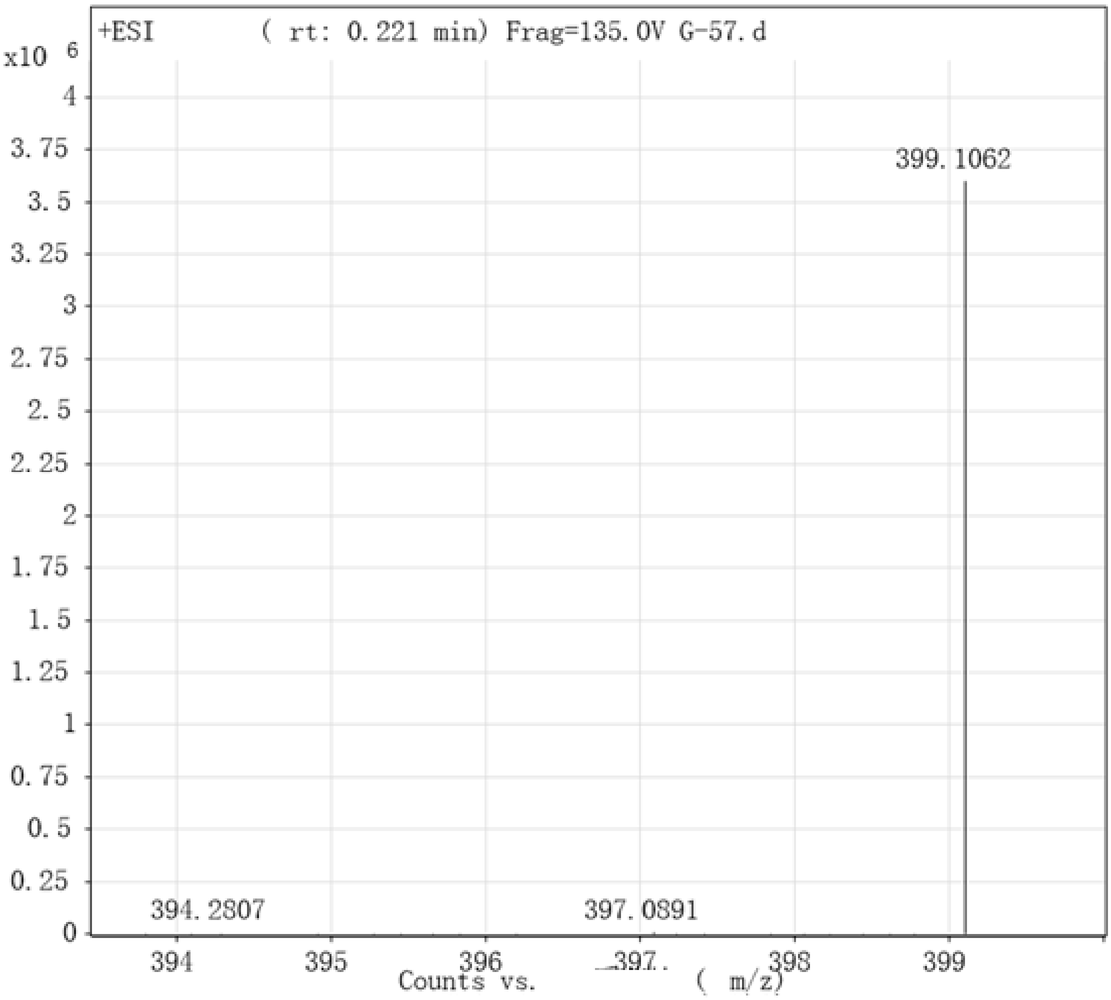

**Figure 37.** $^{1}$H NMR spectrum of **13**

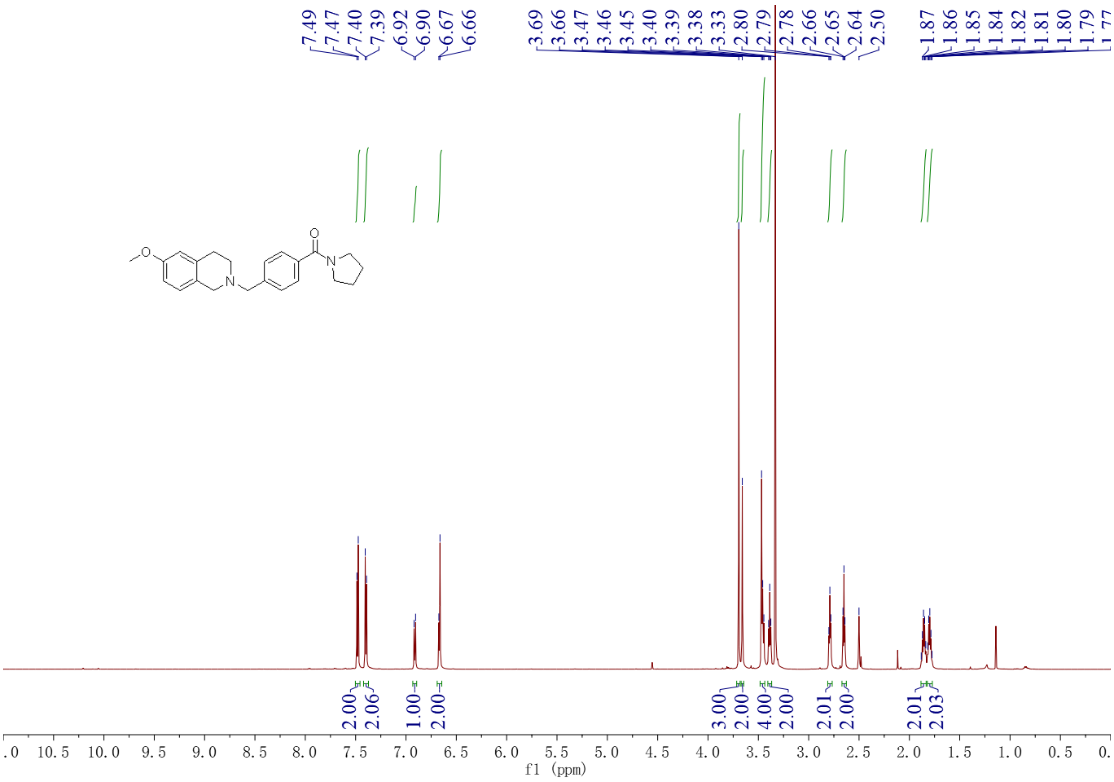

**Figure 38.** $^{13}$C NMR spectrum of **13**

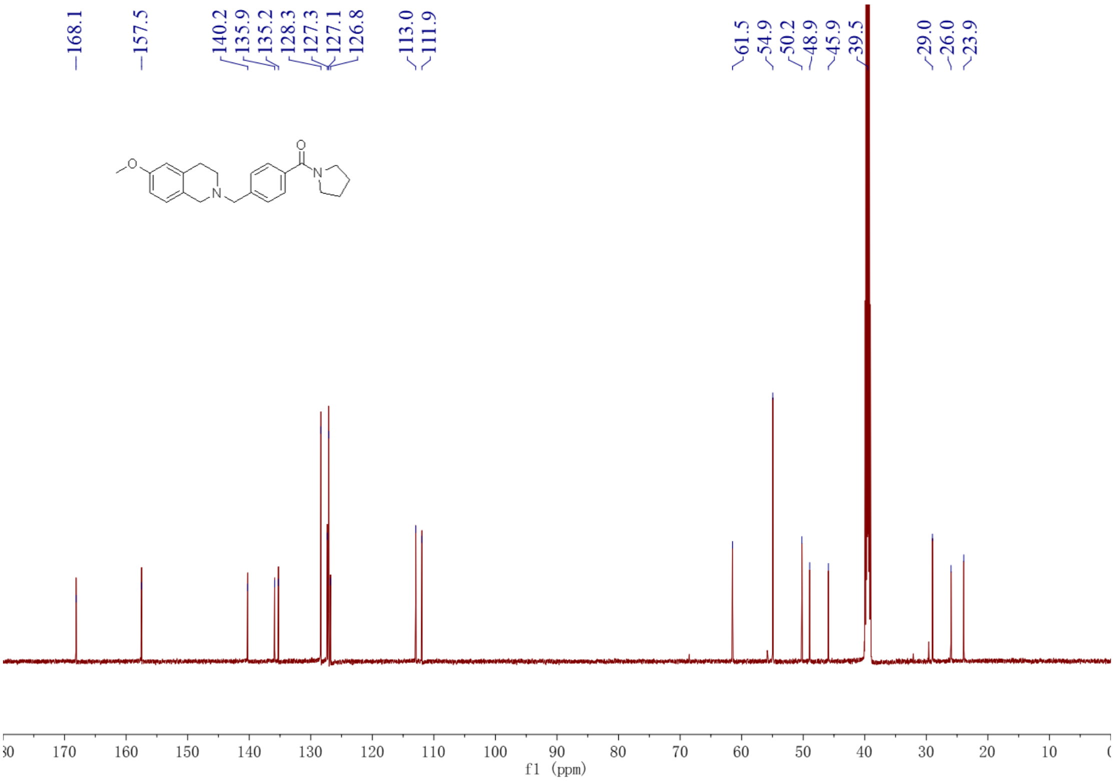

**Figure 39.** HRMS spectrum of **13**

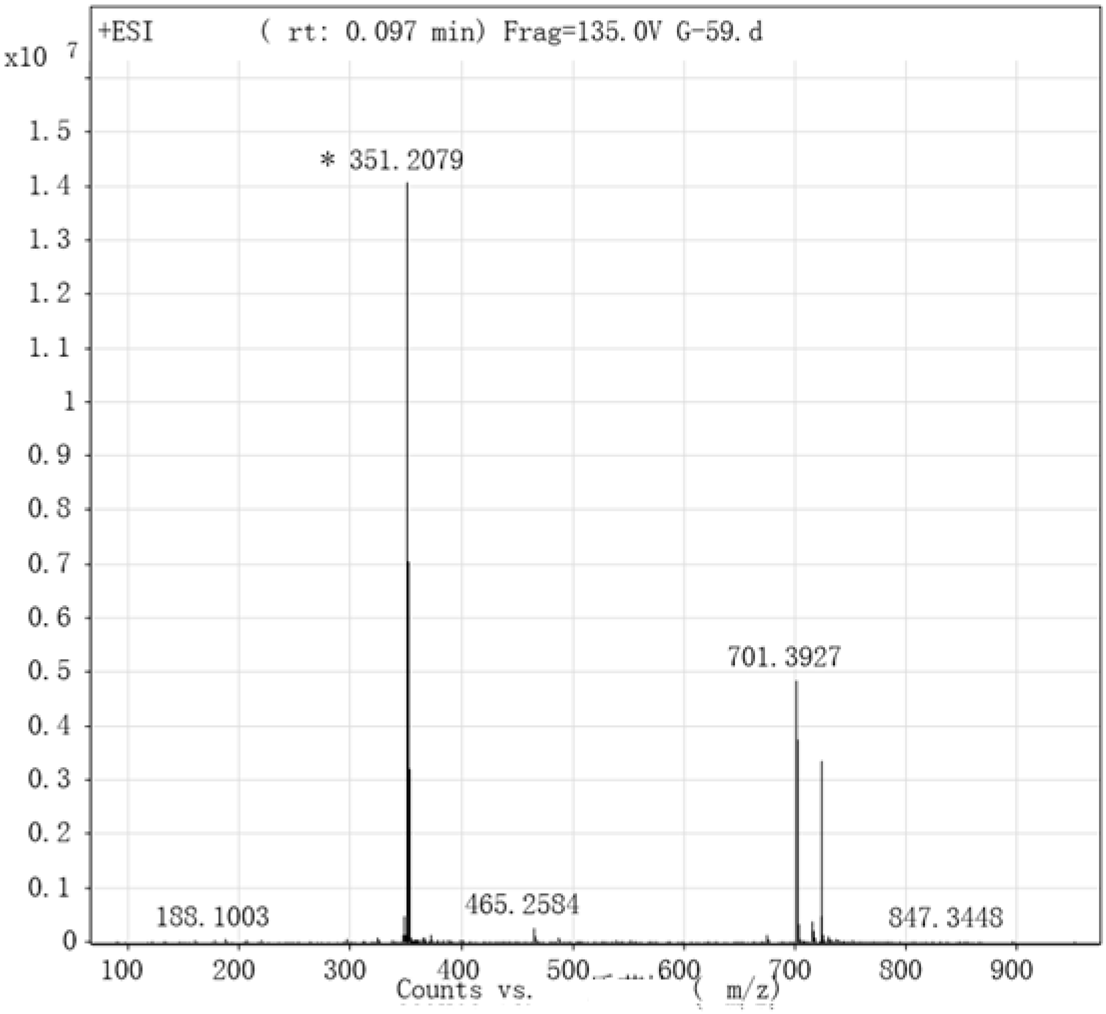

**Figure 40.** $^{1}$H NMR spectrum of **14**

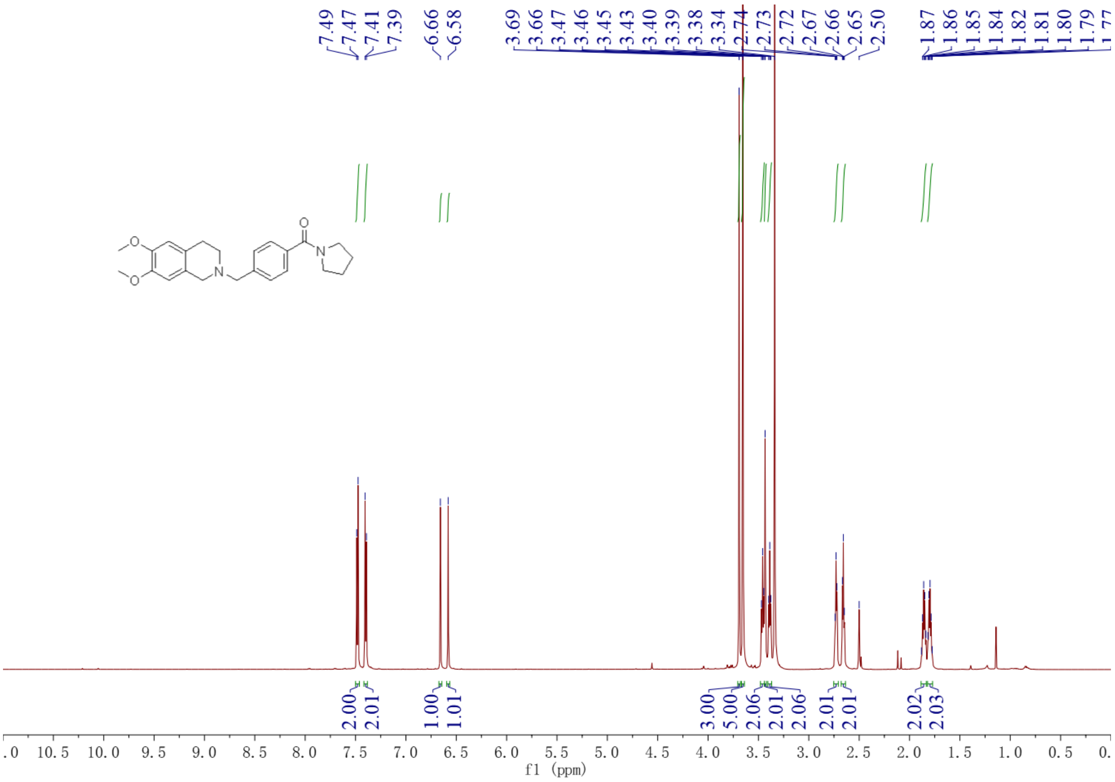

**Figure 41.** $^{13}$C NMR spectrum of **14**

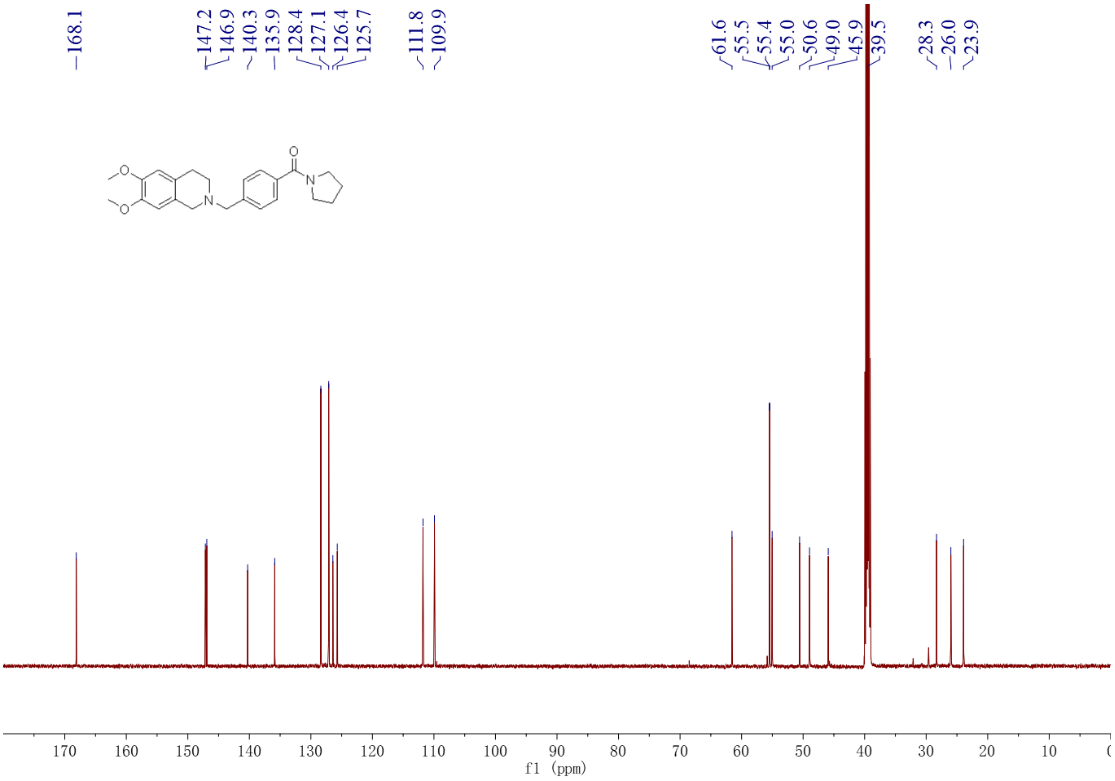

**Figure 42.** HRMS spectrum of **14**

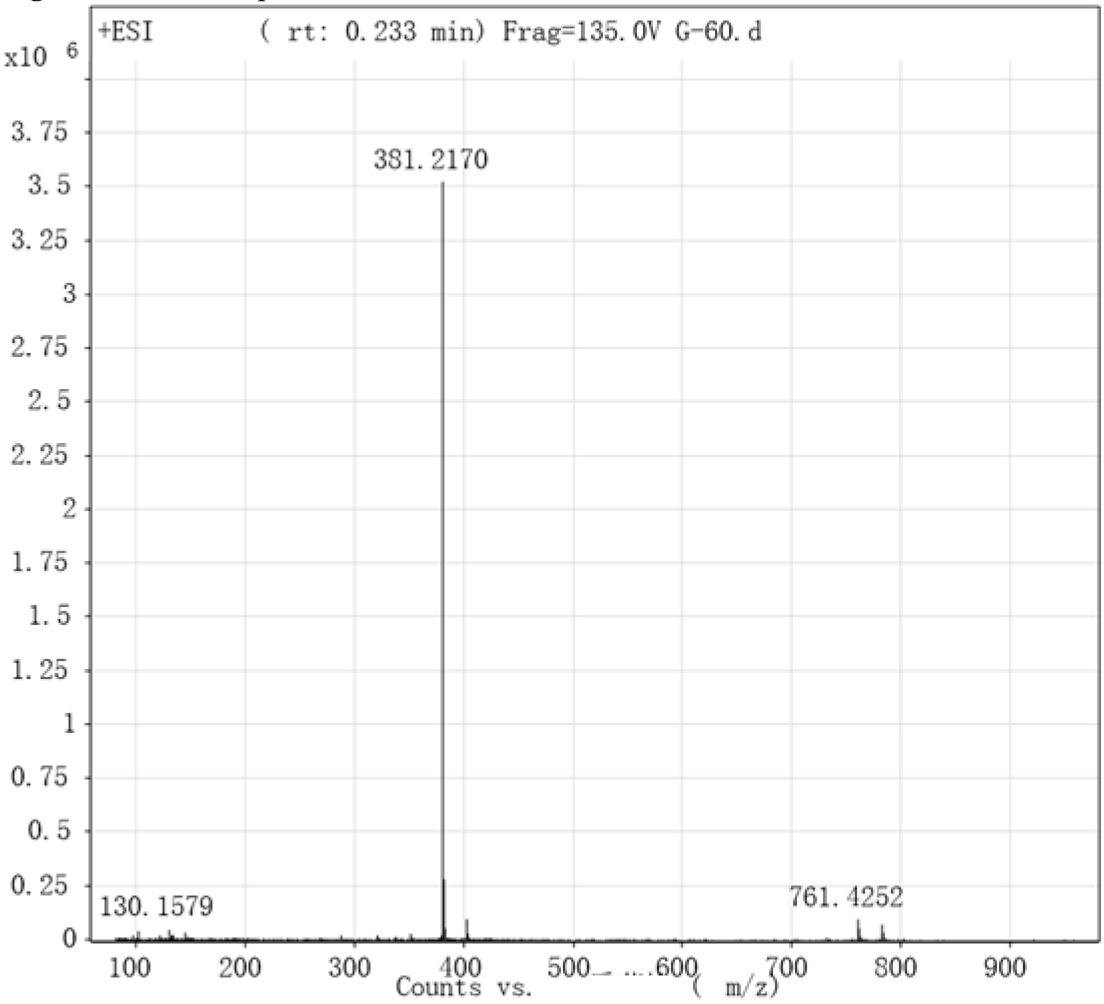

**Figure 43.** $^{1}$H NMR spectrum of **15**

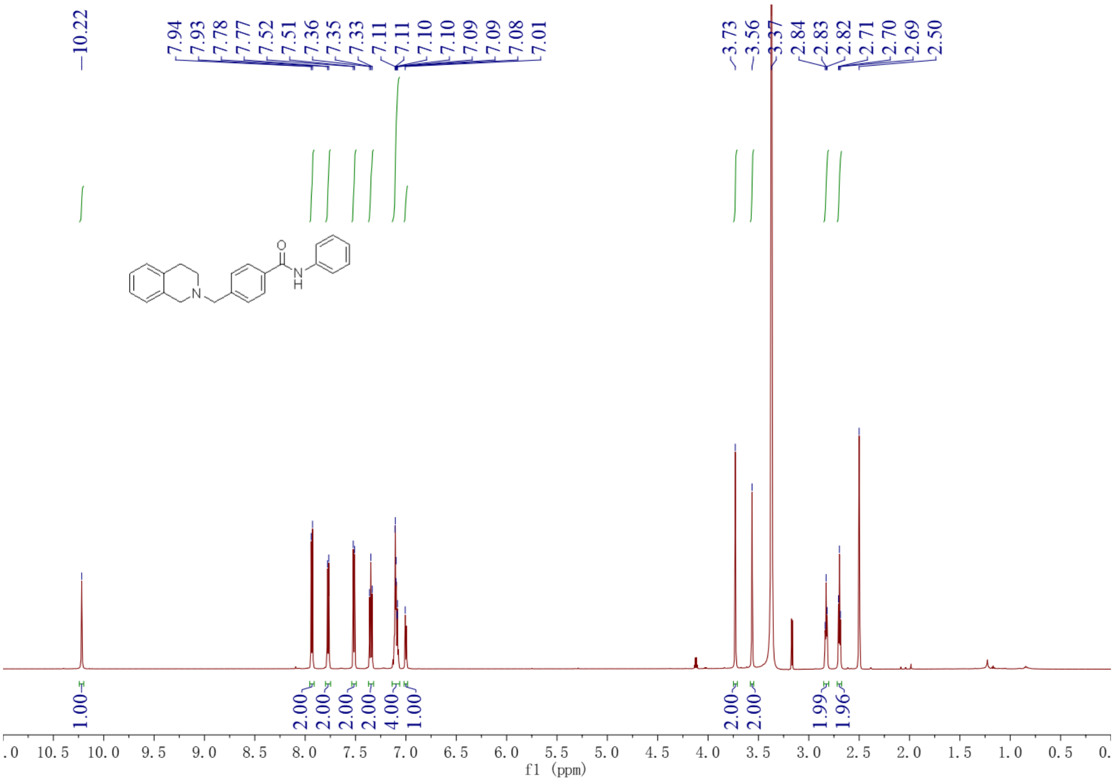

**Figure 44.** $^{13}$C NMR spectrum of **15**

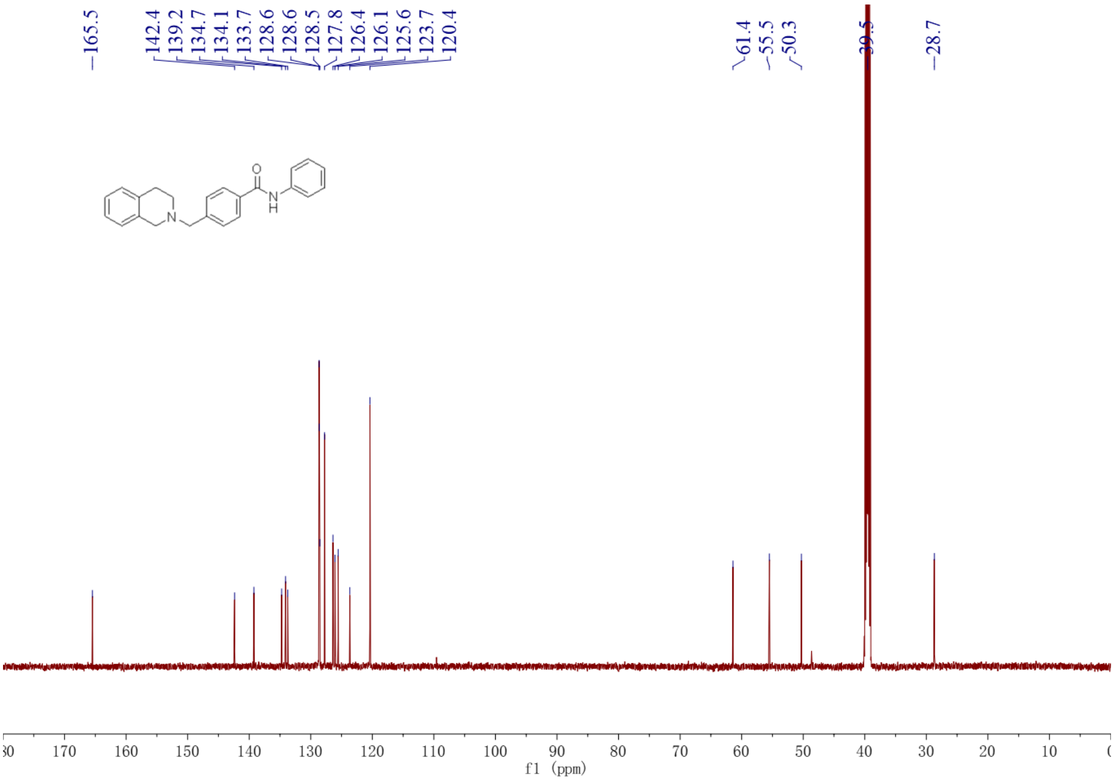

**Figure 45.** HRMS spectrum of **15**

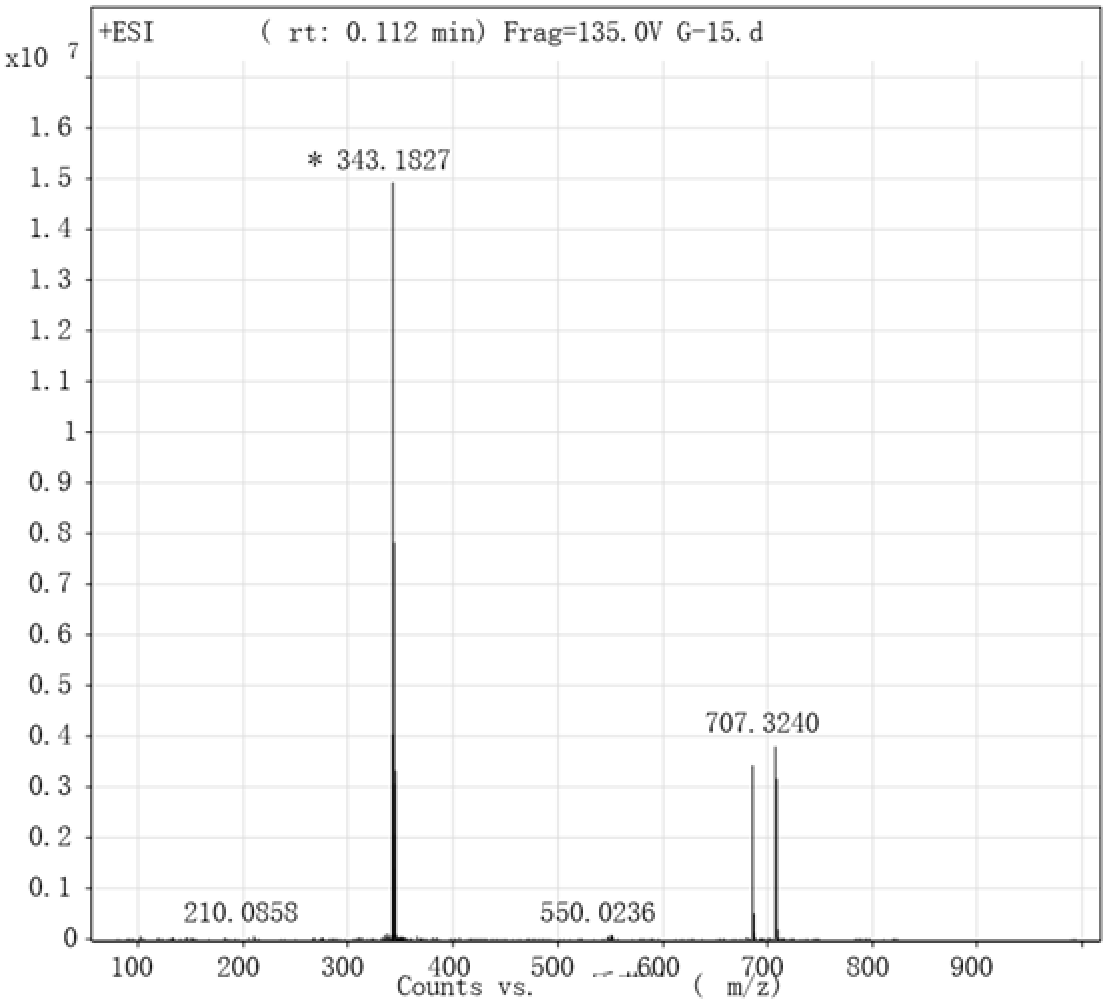

**Figure 46.** $^{1}$H NMR spectrum of **16**

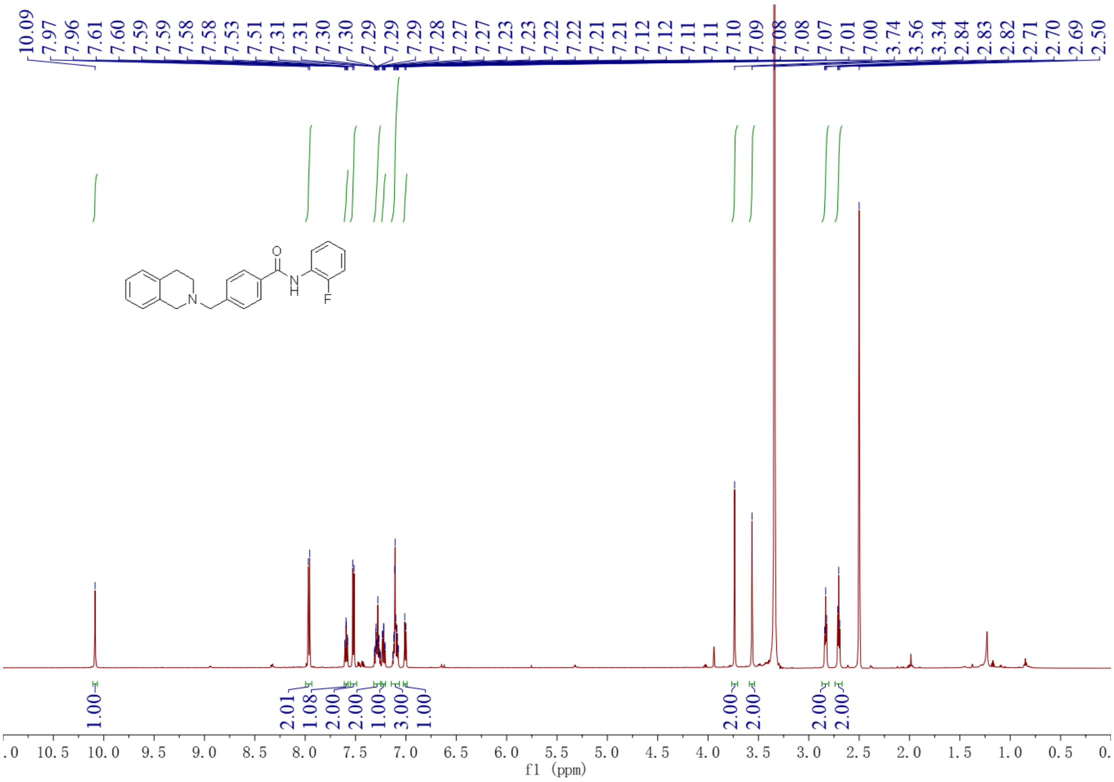


**Figure 47.** $^{13}$C NMR spectrum of **16**

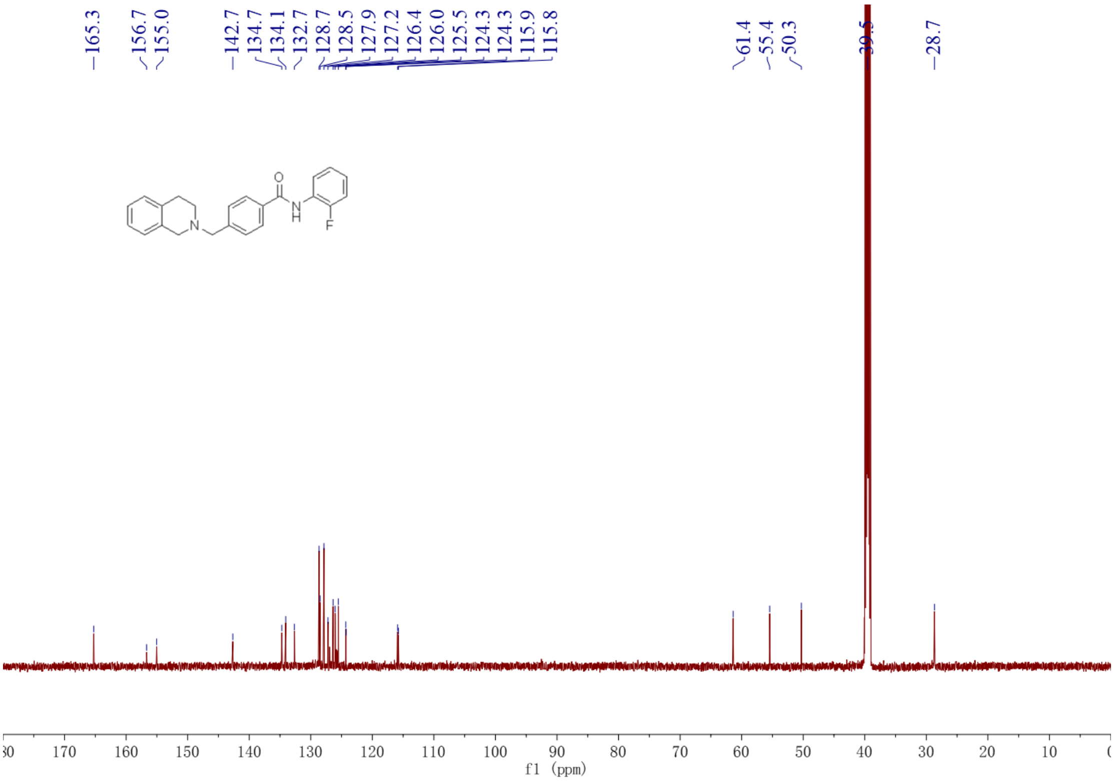

**Figure 48.** HRMS spectrum of **16**

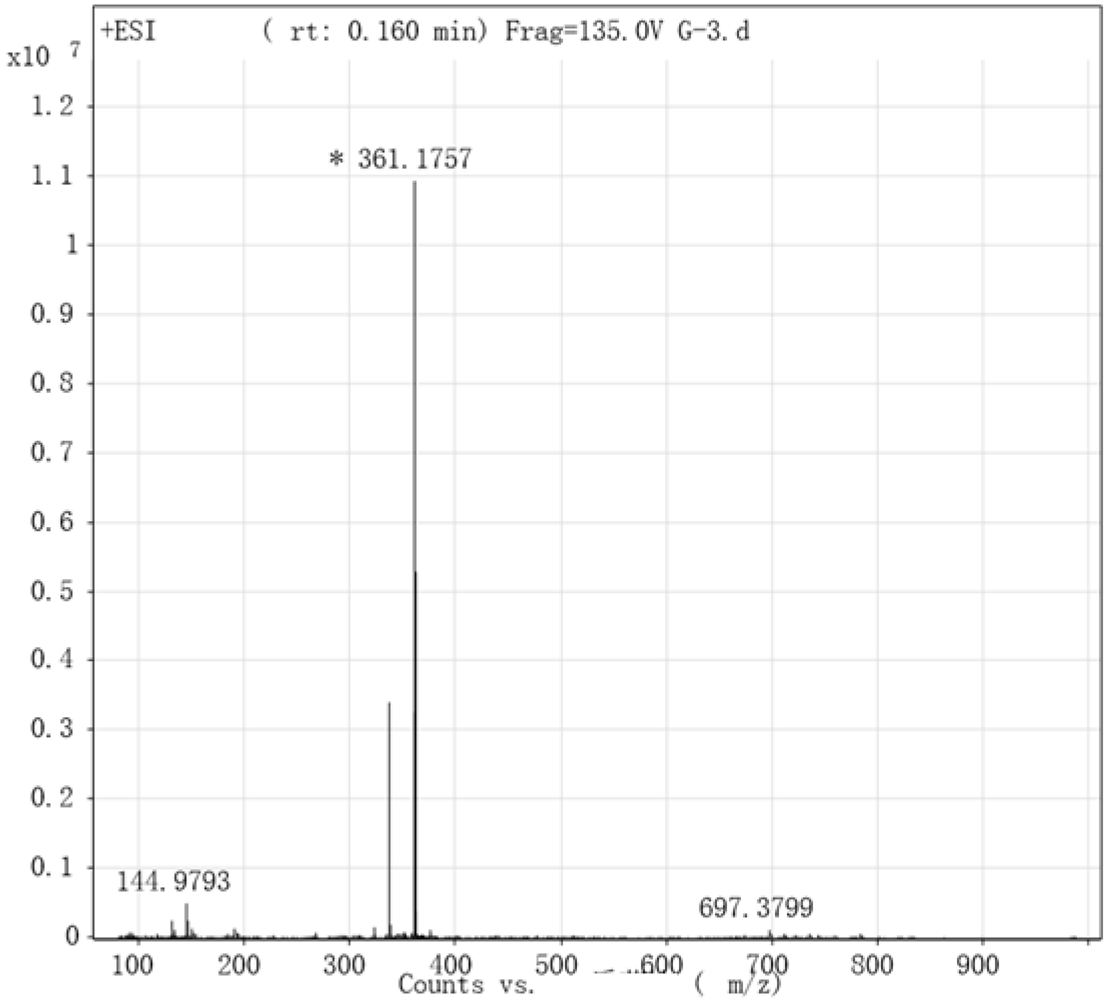

**Figure 49.** $^{1}$H NMR spectrum of **17**

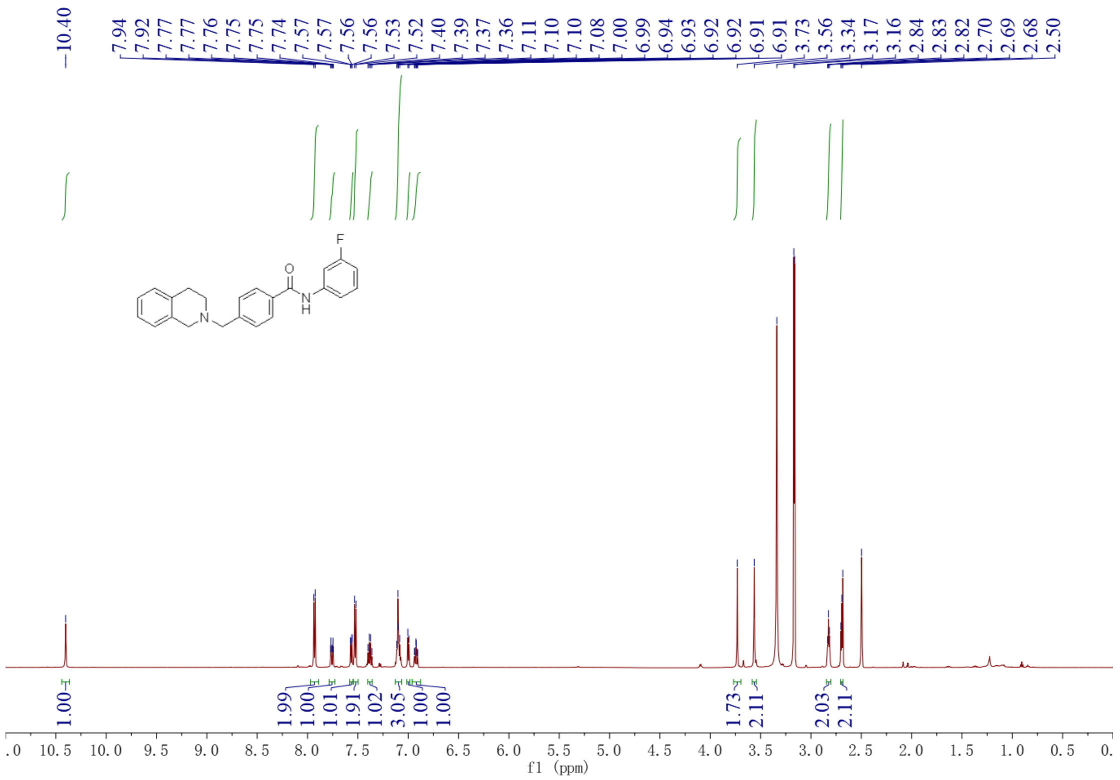


**Figure 50.** $^{13}$C NMR spectrum of **17**

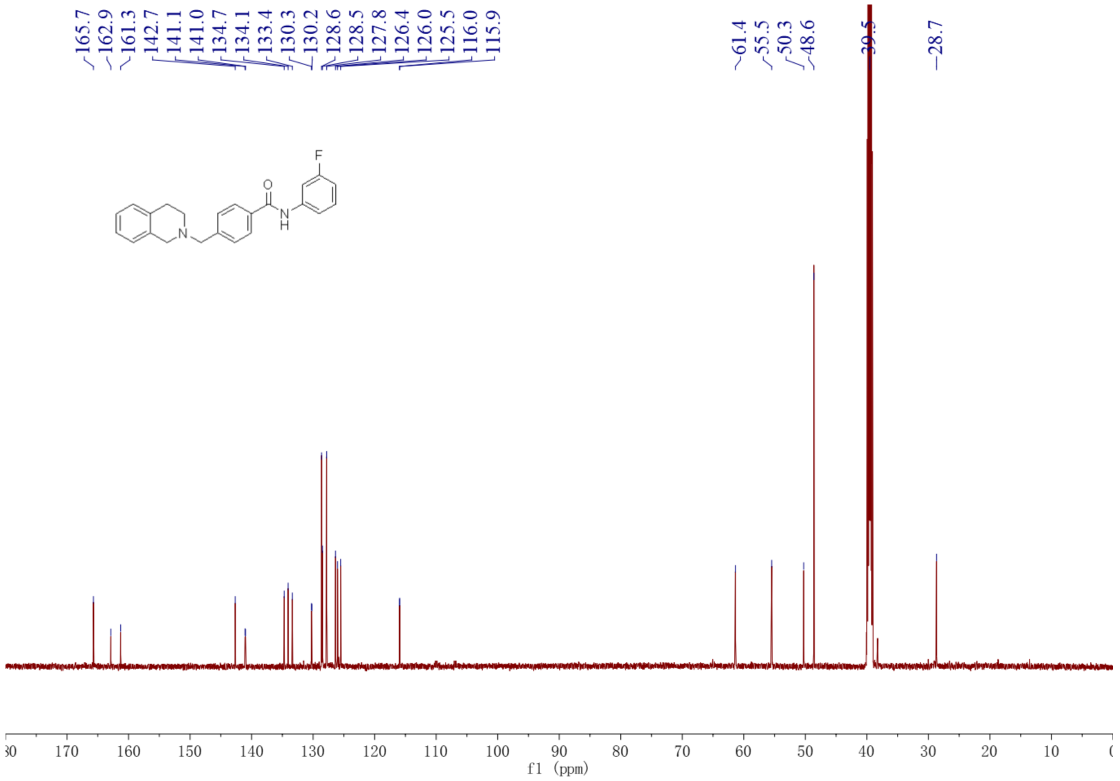

**Figure 51.** HRMS spectrum of **17**

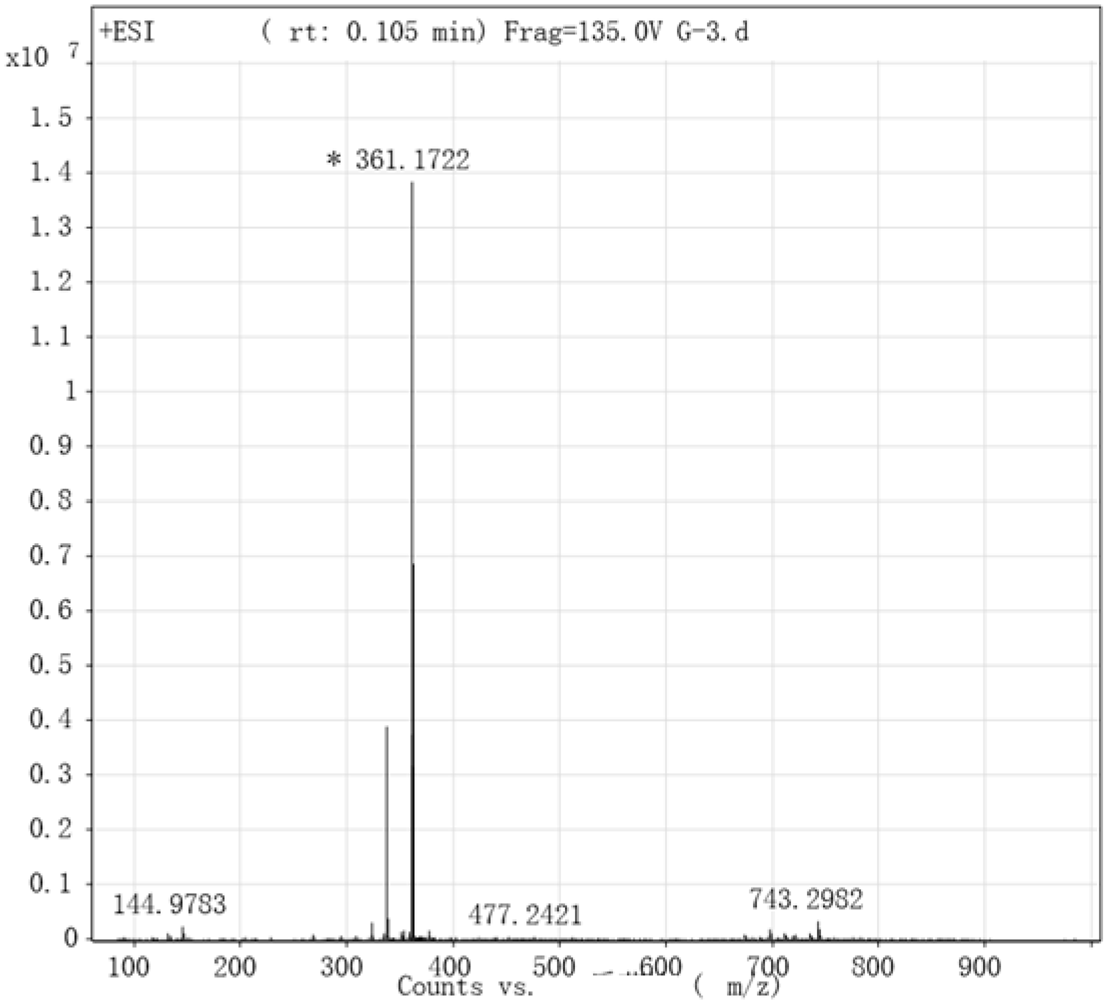

**Figure 52.** $^{1}$H NMR spectrum of **18**

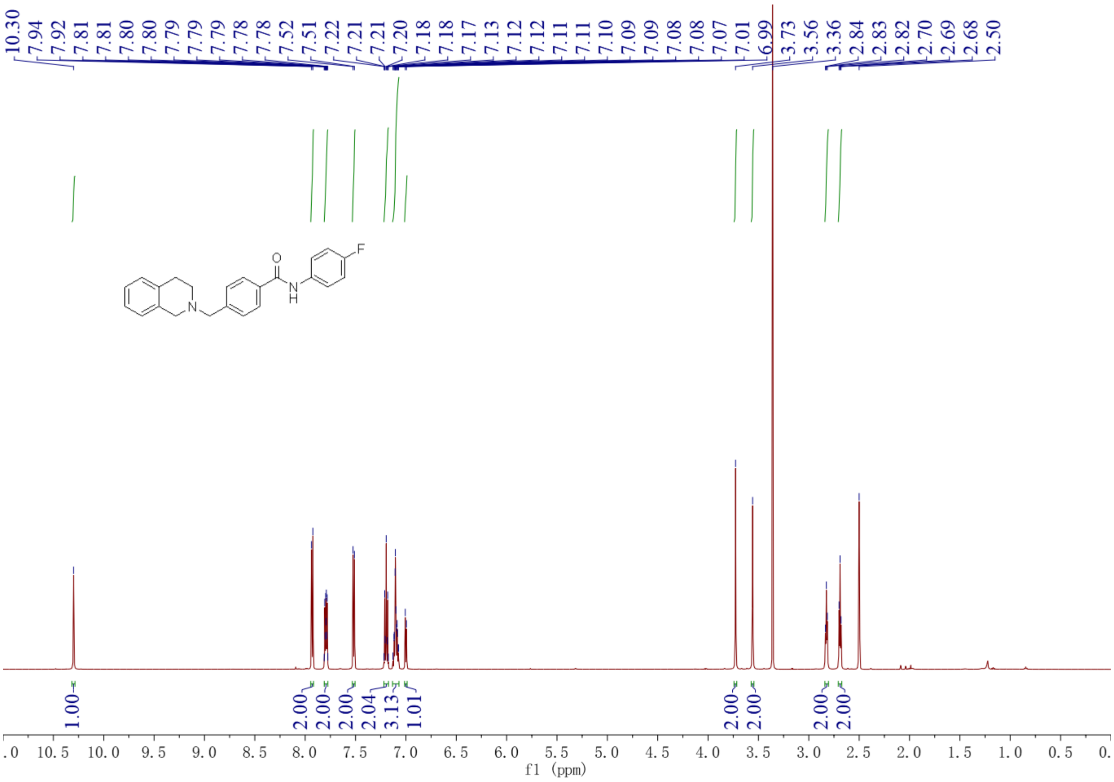

**Figure 53.** $^{13}$C NMR spectrum of **18**

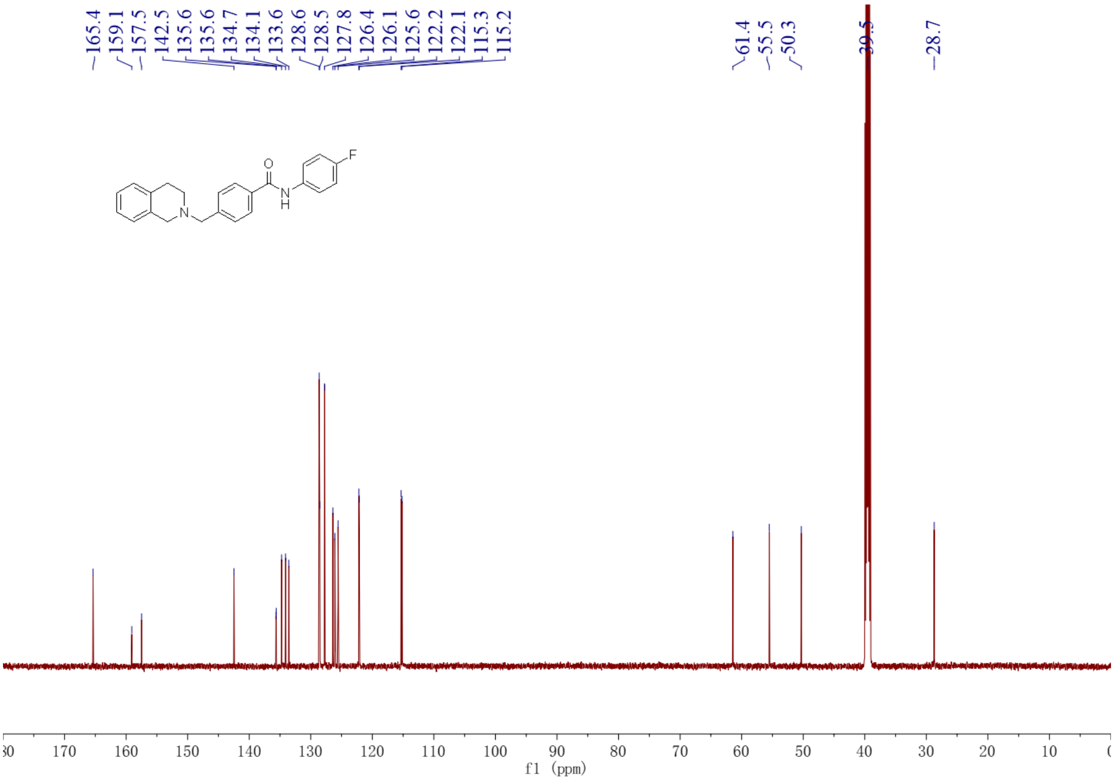

**Figure 54.** HRMS spectrum of **18**

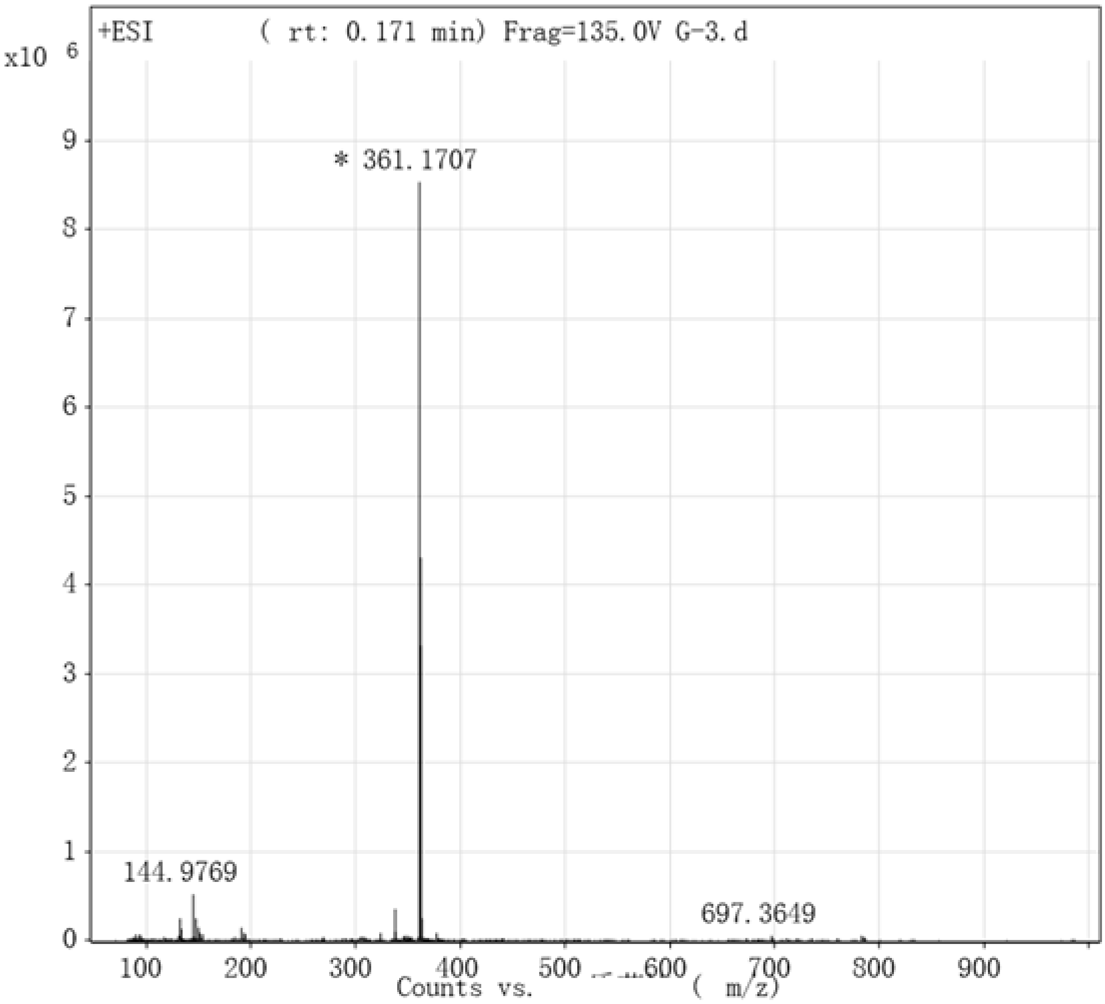

**Figure 55.** $^{1}$H NMR spectrum of **19**

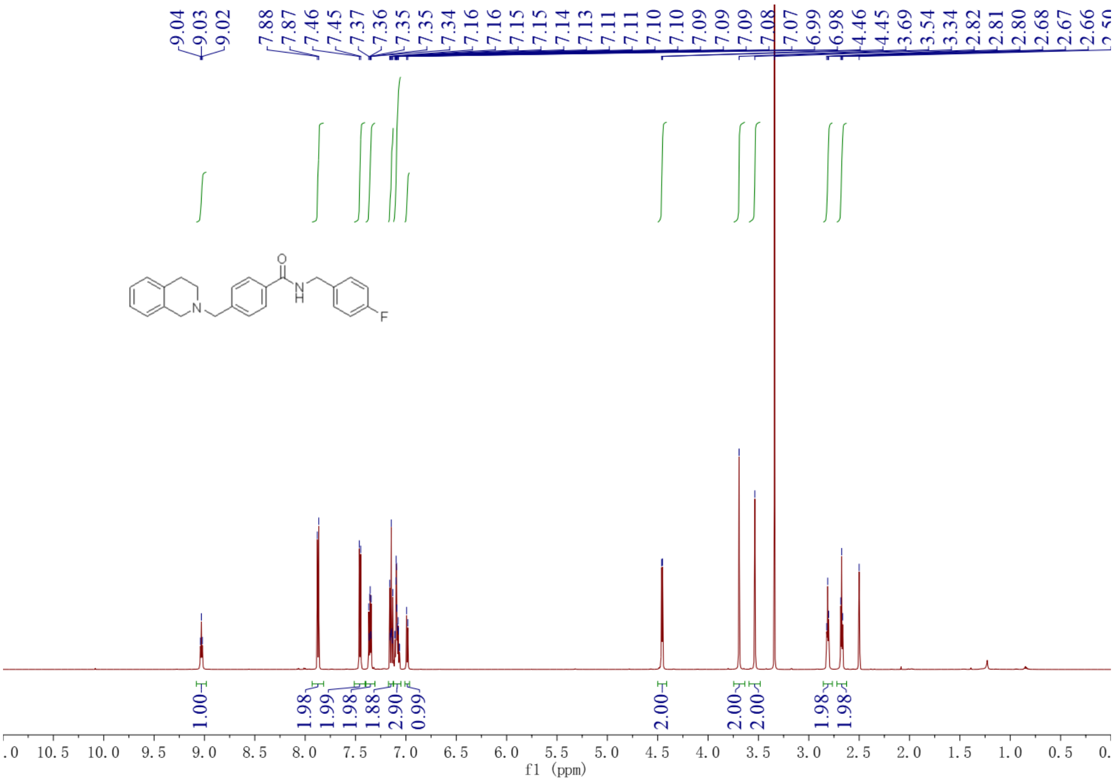


**Figure 56.** $^{13}$C NMR spectrum of **19**

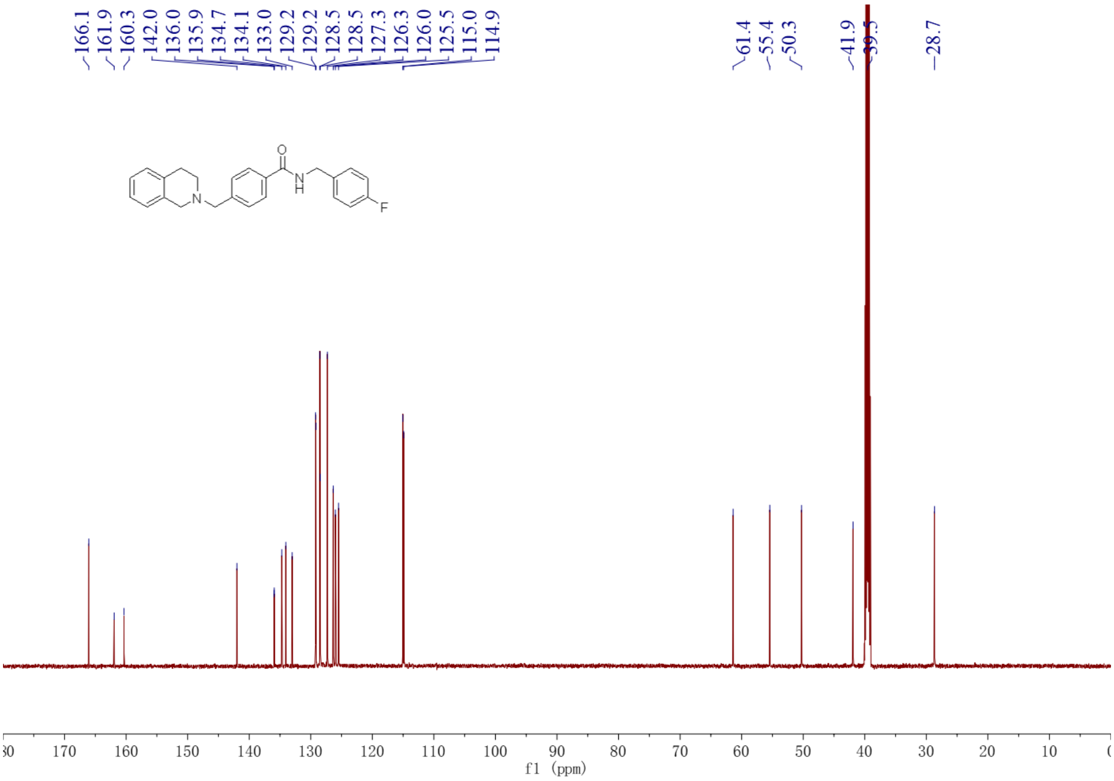

**Figure 57.** HRMS spectrum of **19**

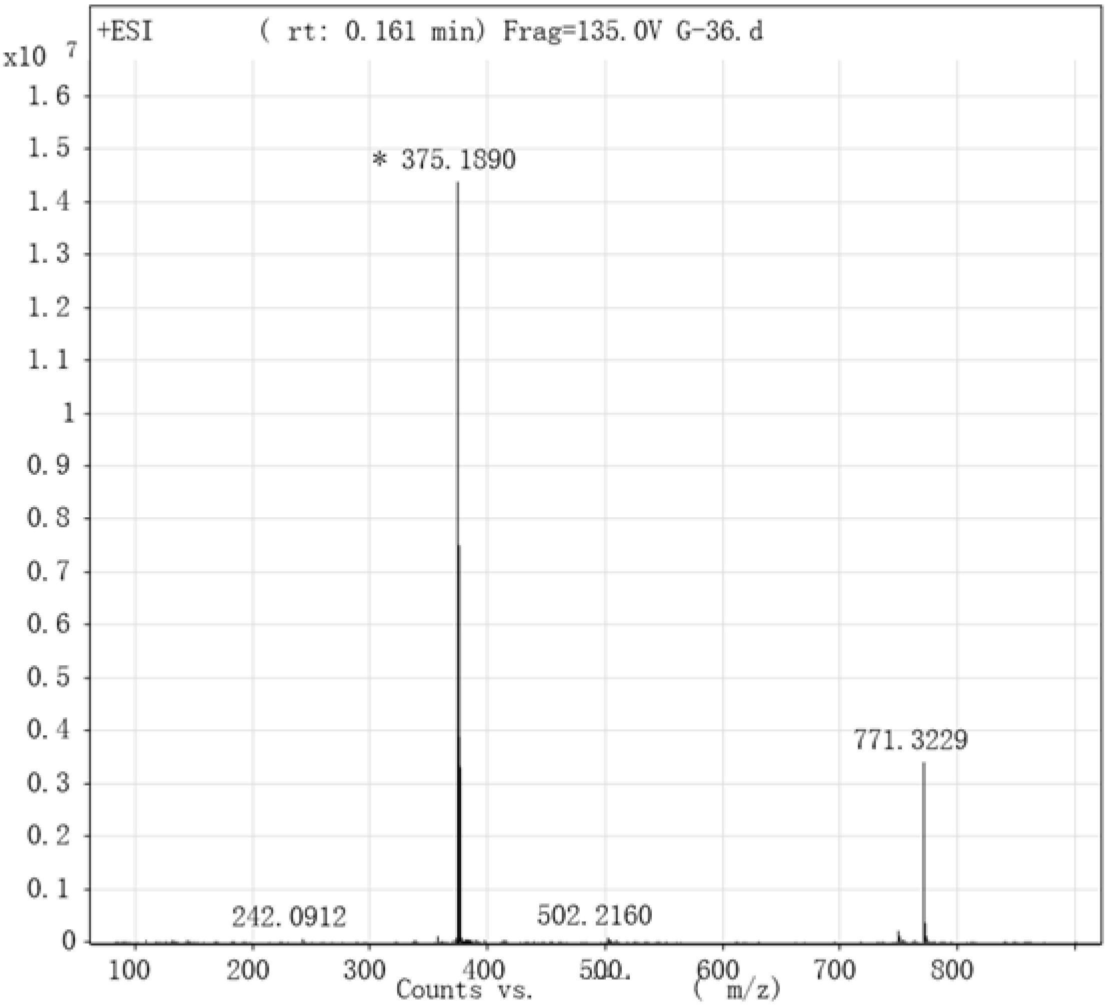

**Figure 58.** $^{1}$H NMR spectrum of **20**

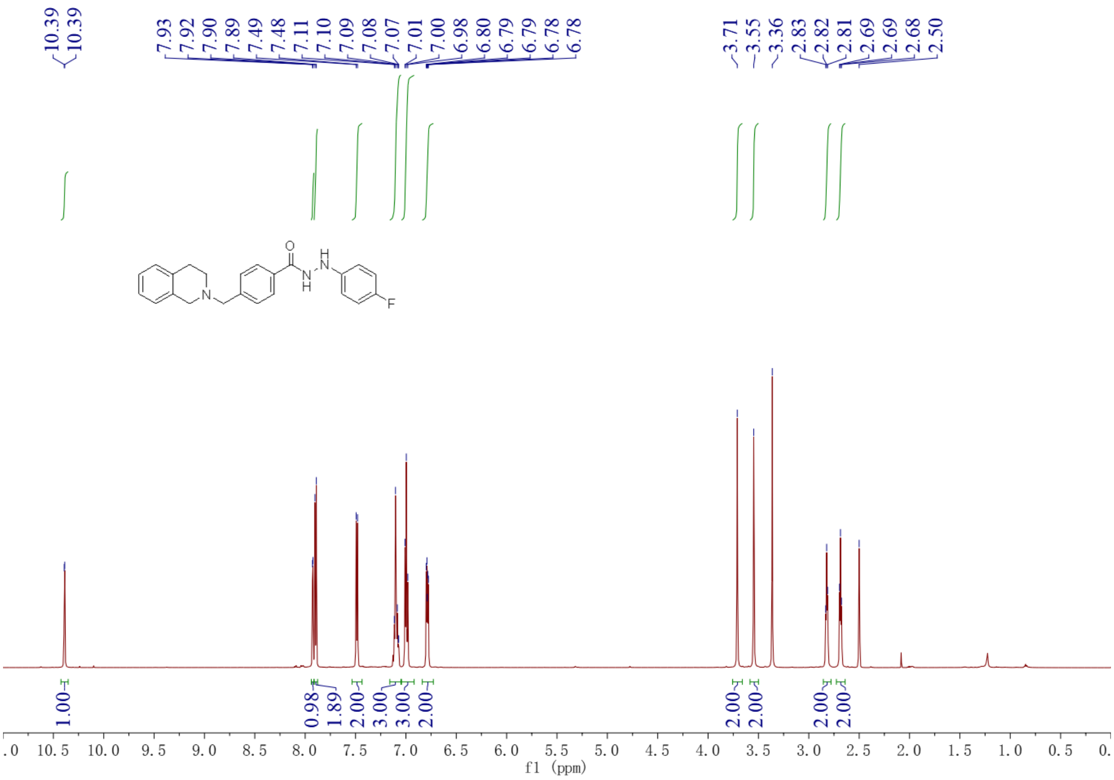

**Figure 59.** $^{13}$C NMR spectrum of **20**

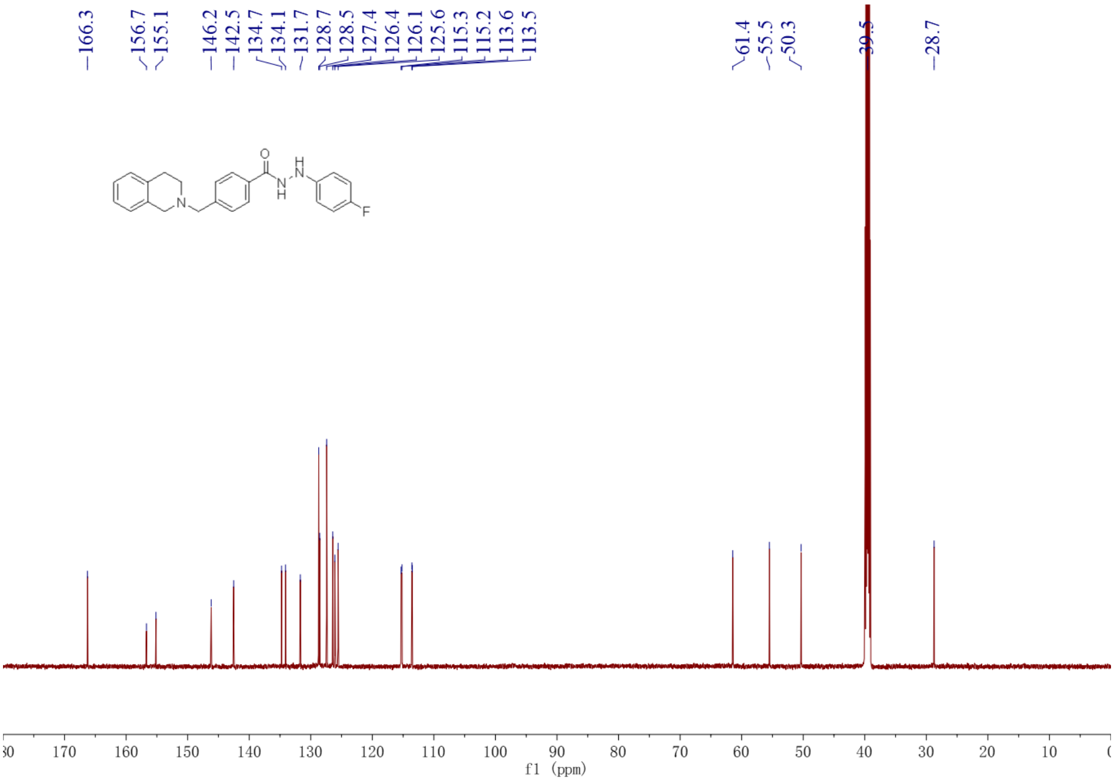

**Figure 60.** HRMS spectrum of **20**

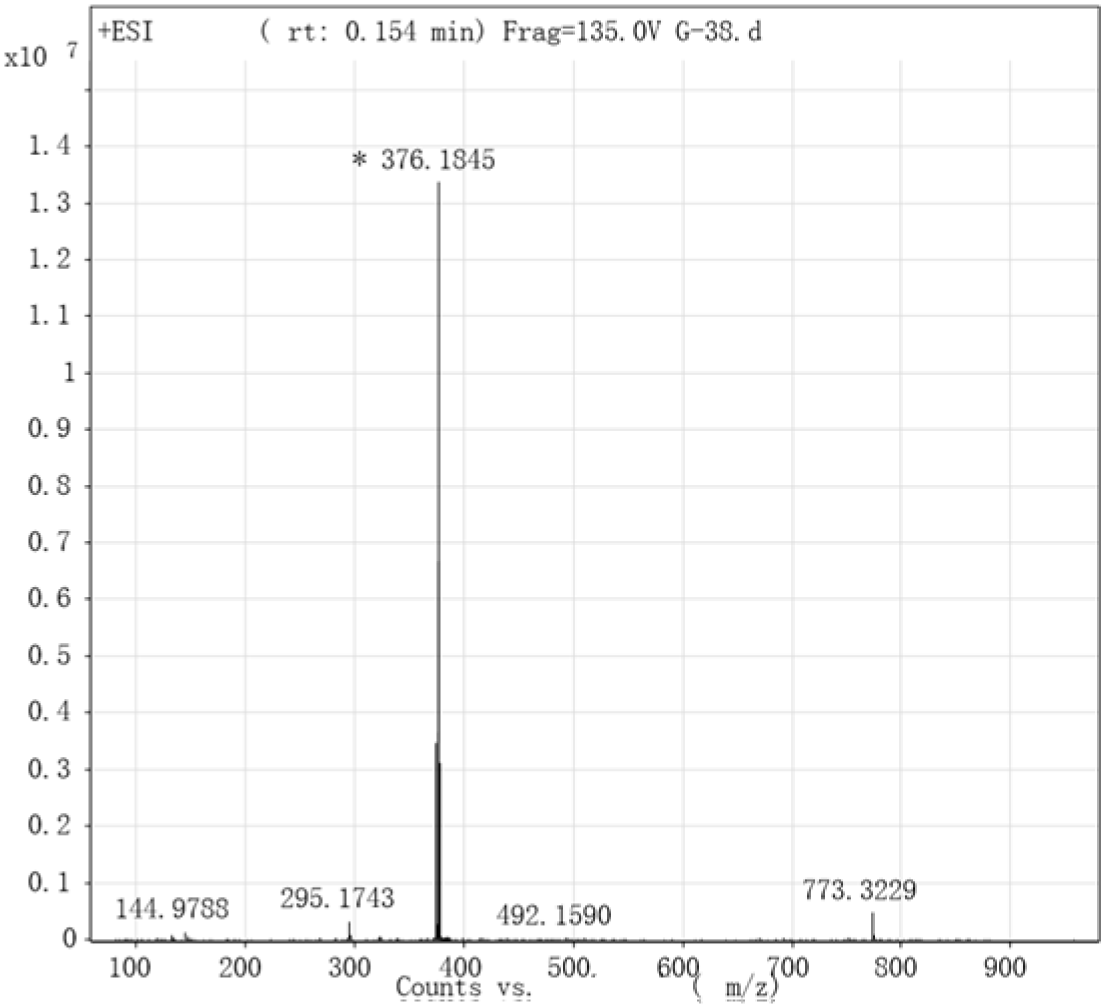

**Figure 61.** $^{1}$H NMR spectrum of **21**

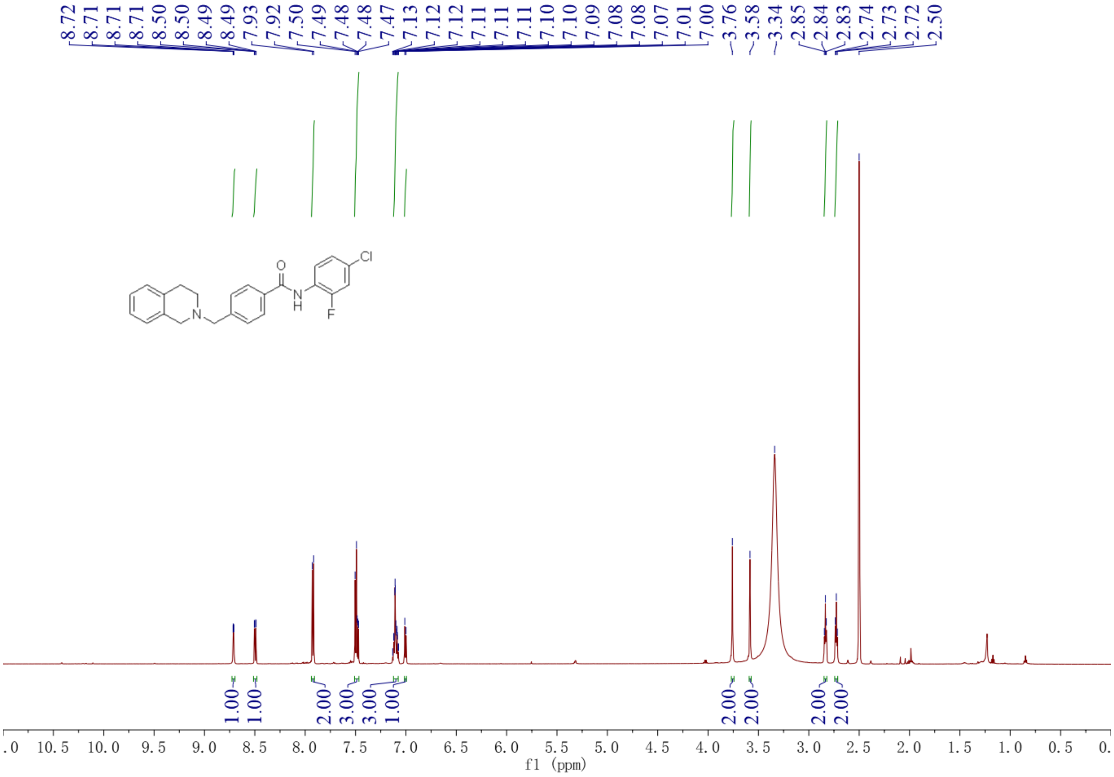


**Figure 62.** $^{13}$C NMR spectrum of **21**

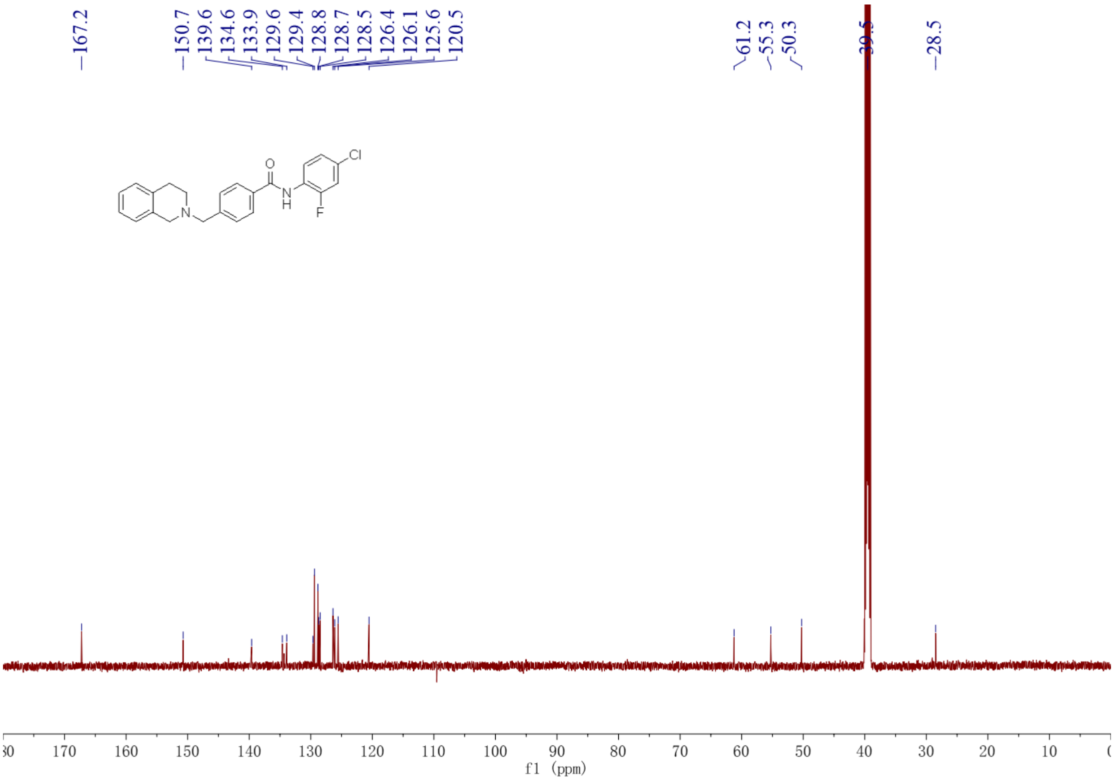

**Figure 63.** HRMS spectrum of **21**

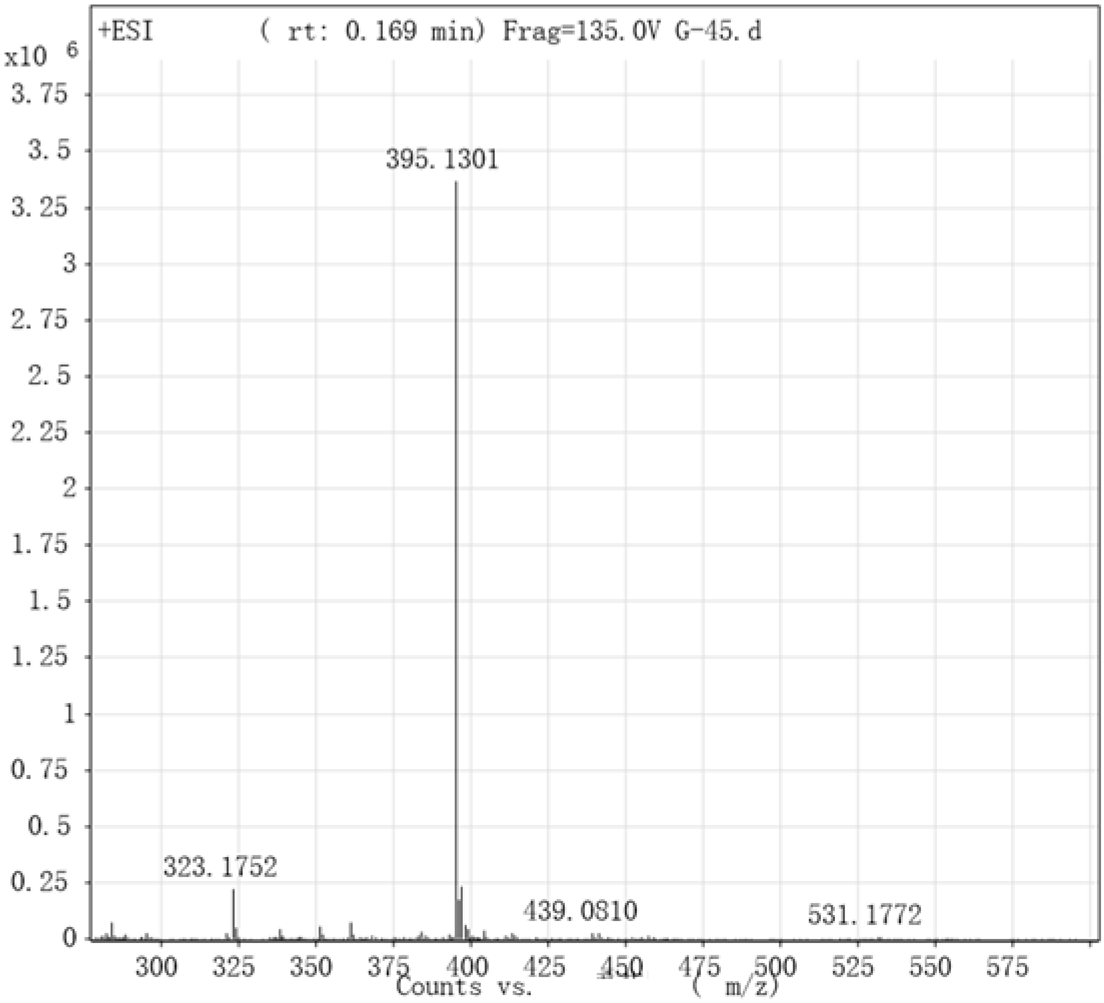

**Figure 64.** $^{1}$H NMR spectrum of **22**

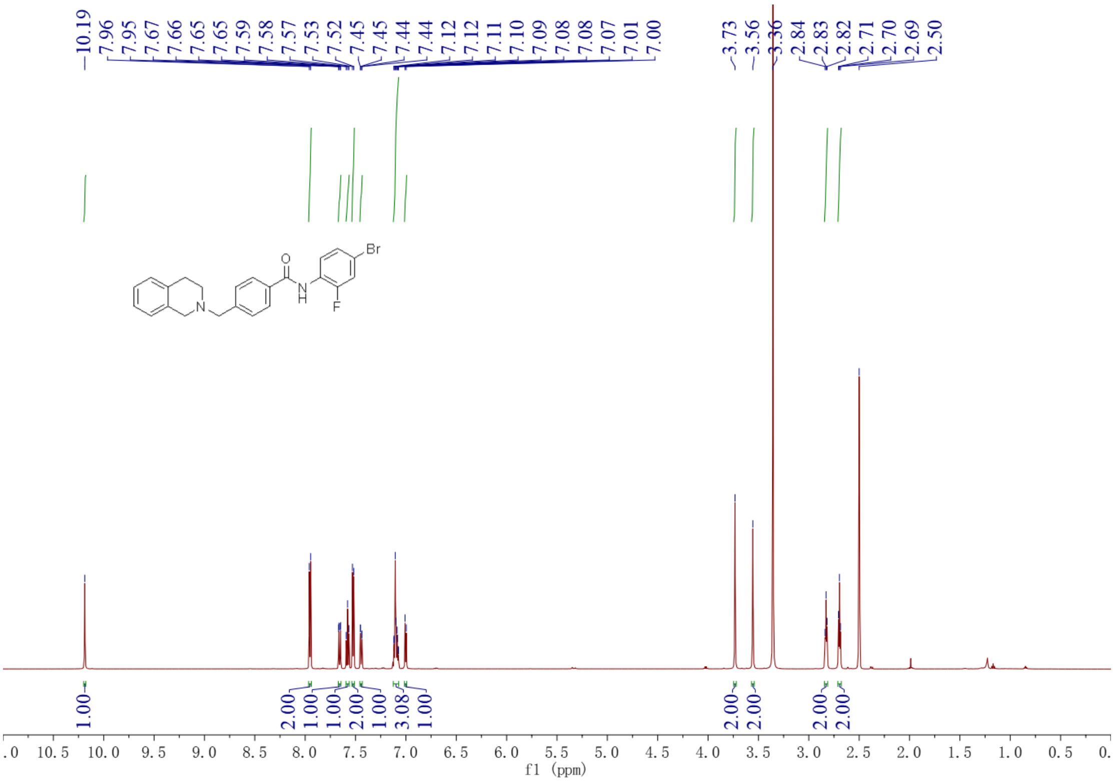


**Figure 65.** $^{13}$C NMR spectrum of **22**

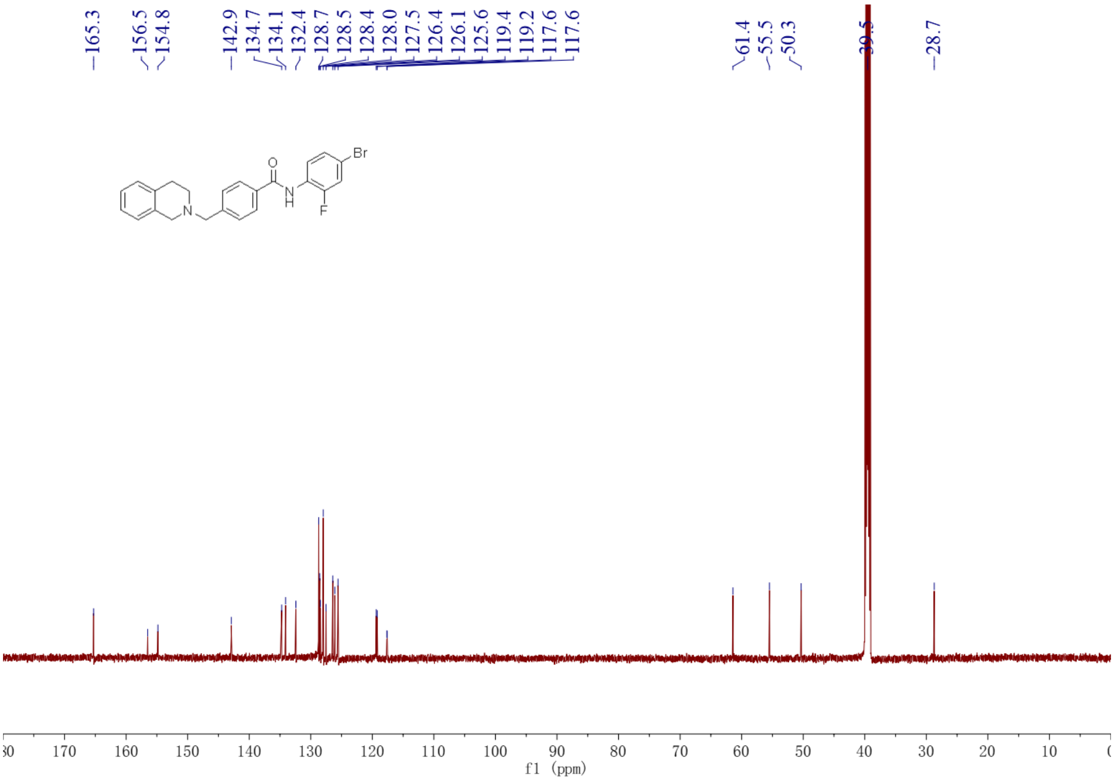

**Figure 66.** HRMS spectrum of **22**

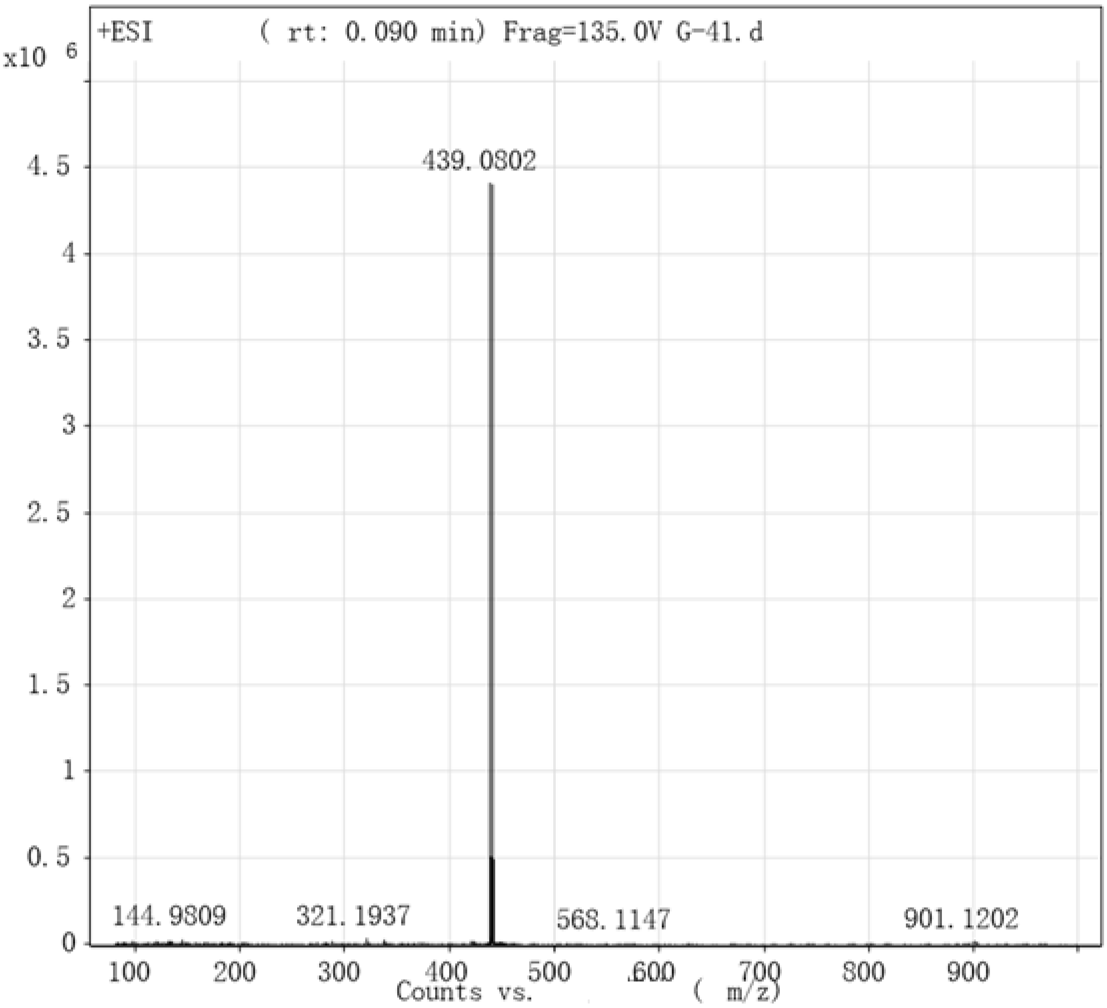

**Figure 67.** $^{1}$H NMR spectrum of **23**

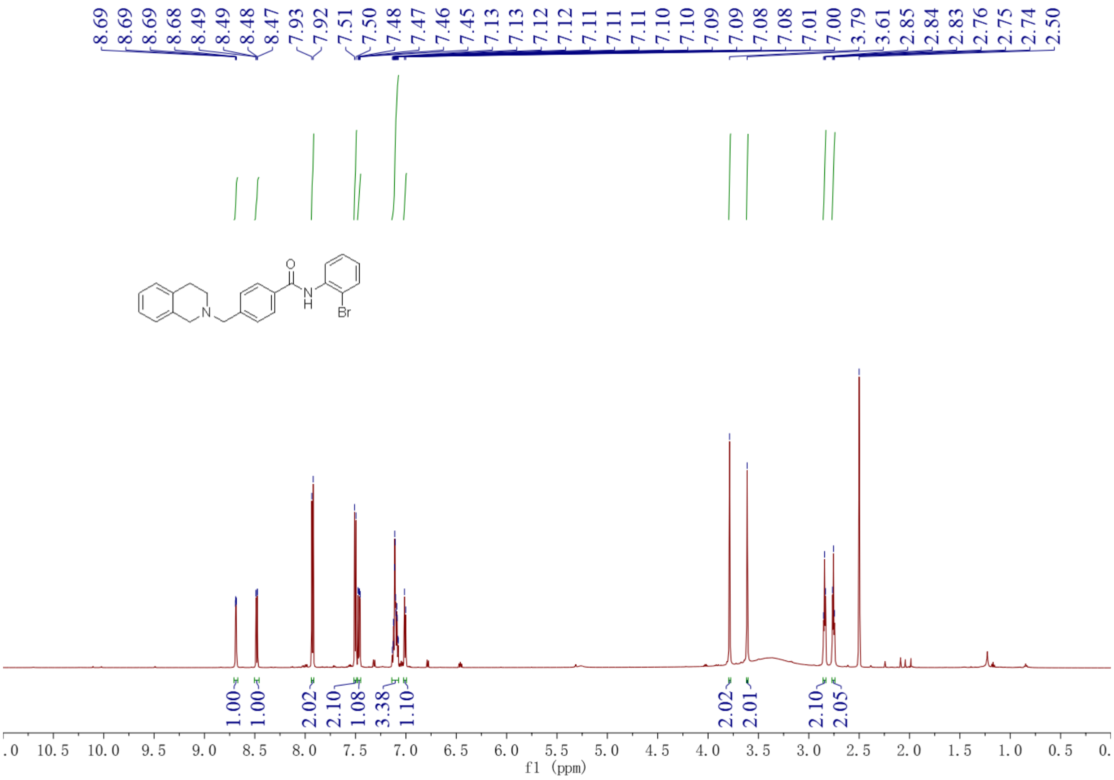

**Figure 68.** $^{13}$C NMR spectrum of **23**

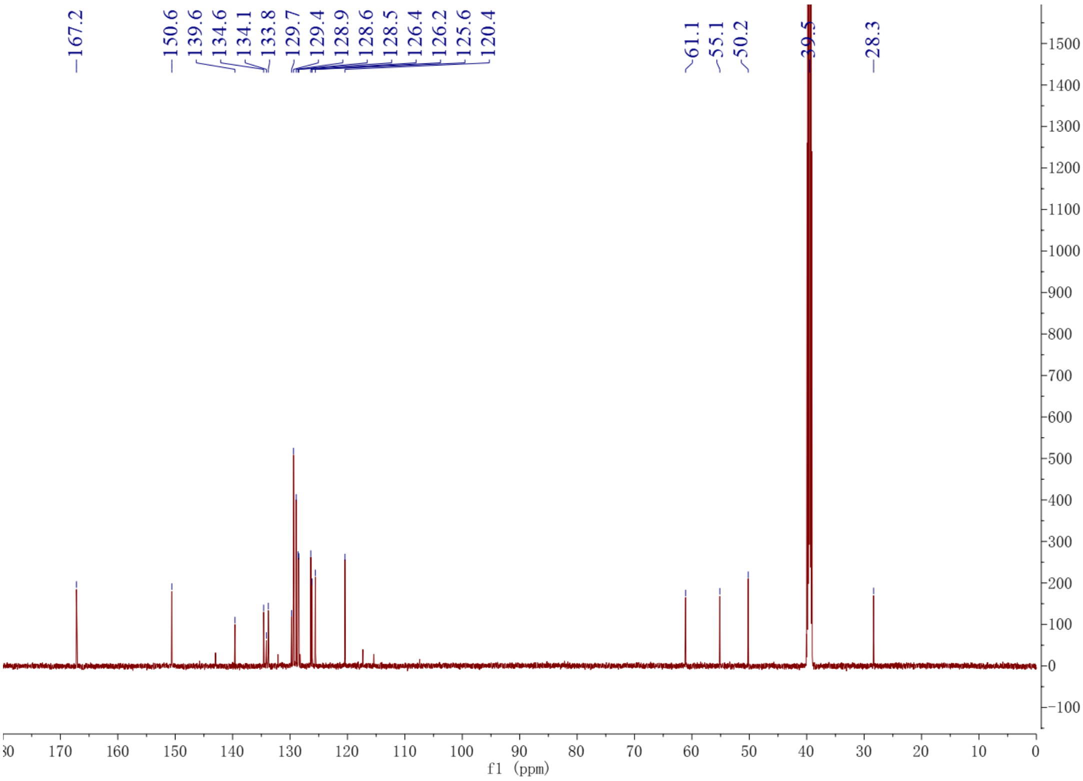

**Figure 69.** HRMS spectrum of **23**

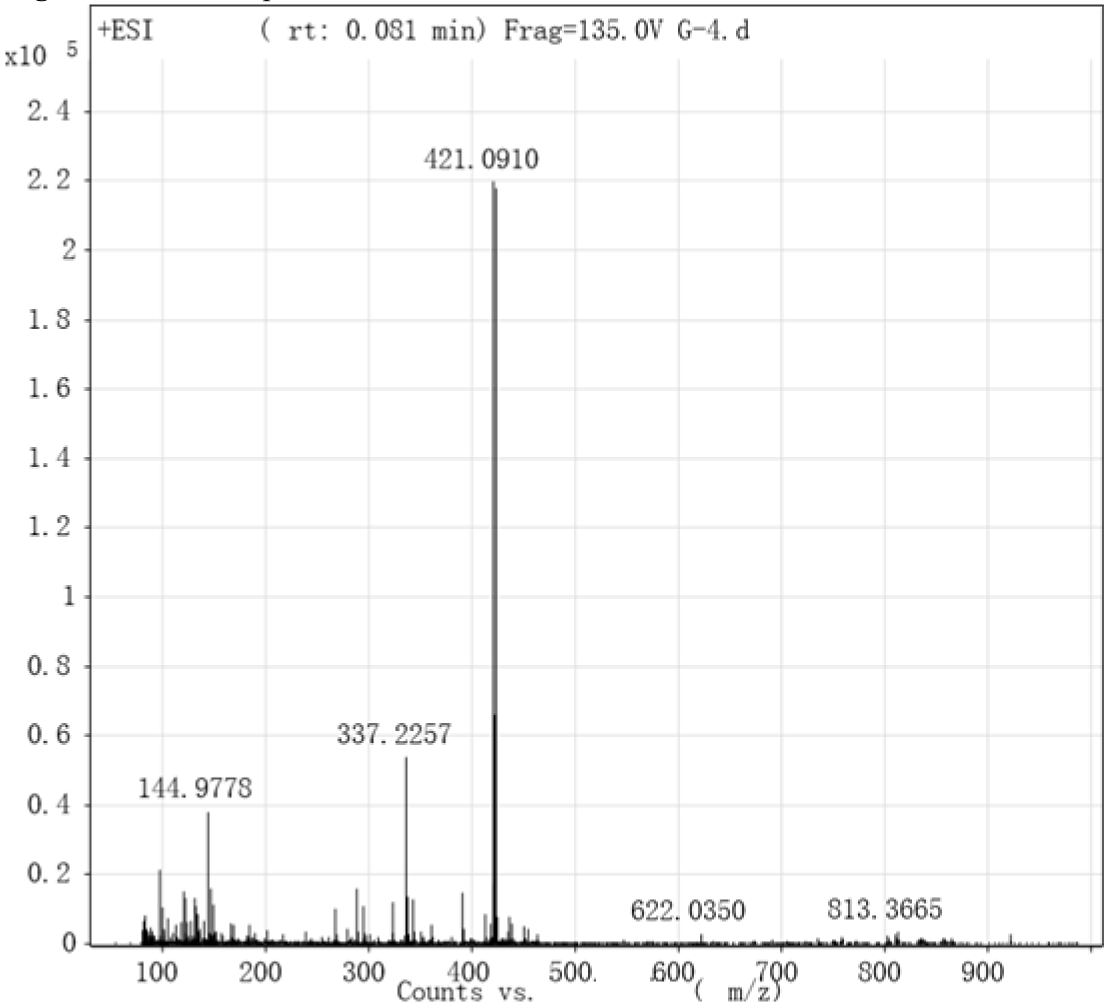

**Figure 70.** $^{1}$H NMR spectrum of **24**

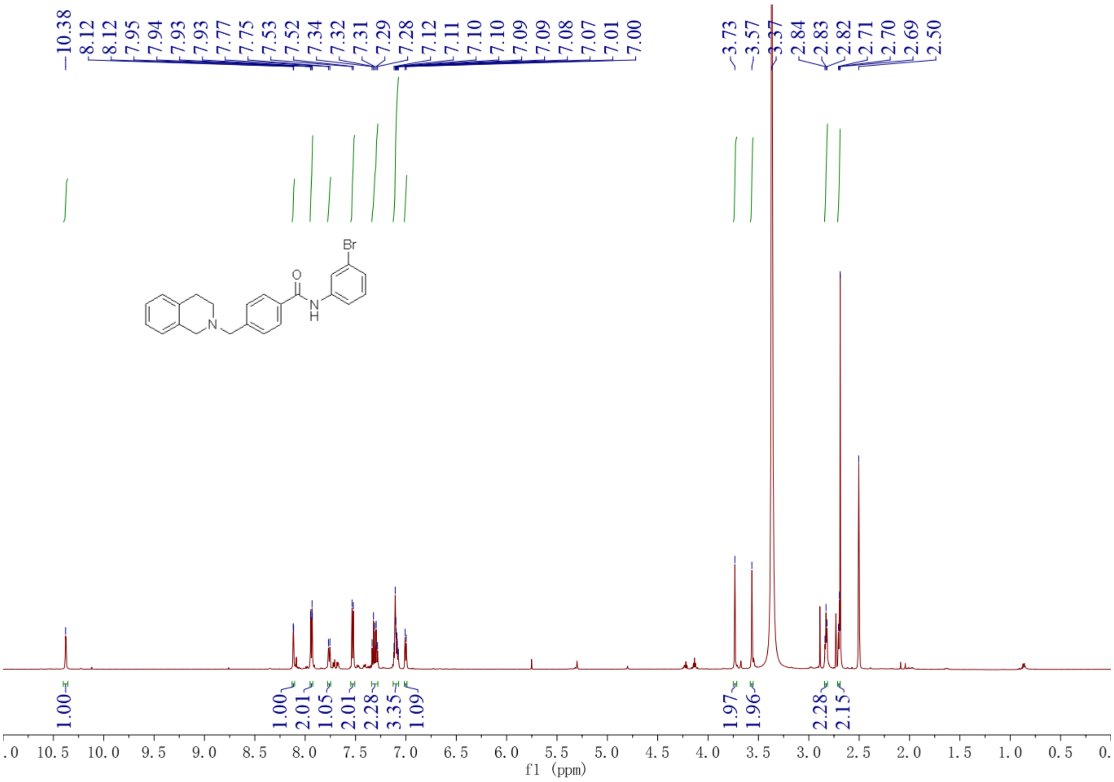

**Figure 71.** $^{13}$C NMR spectrum of **24**

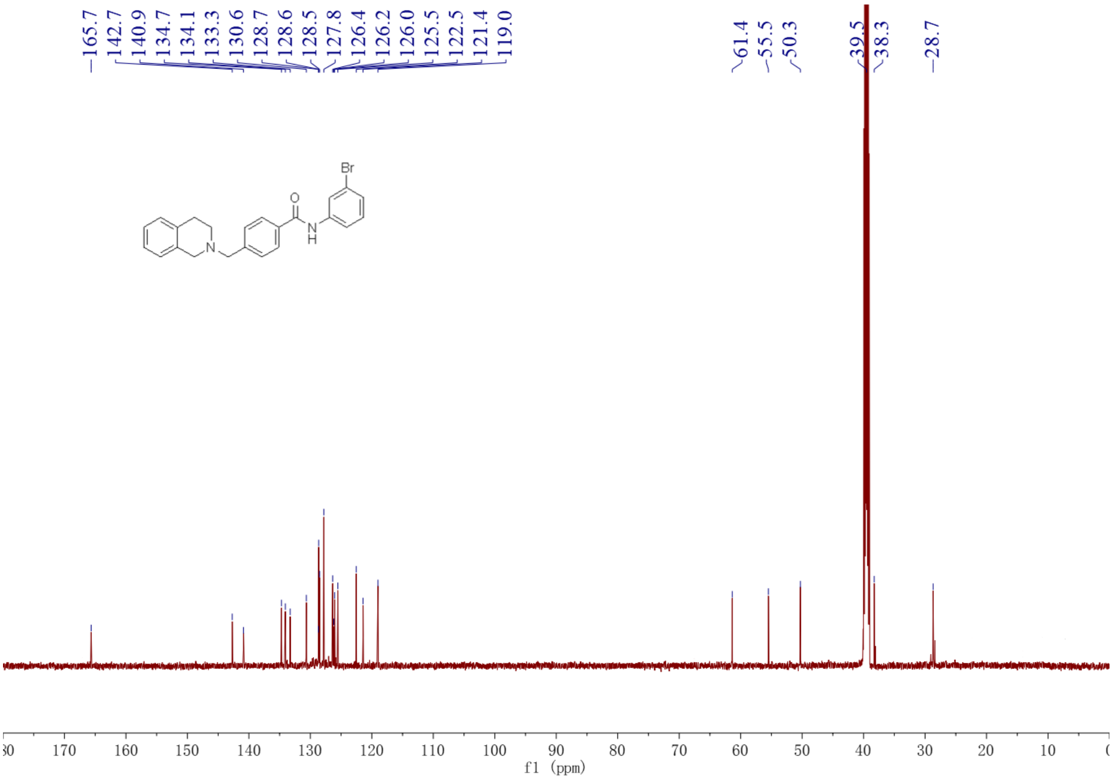

**Figure 72.** HRMS spectrum of **24**

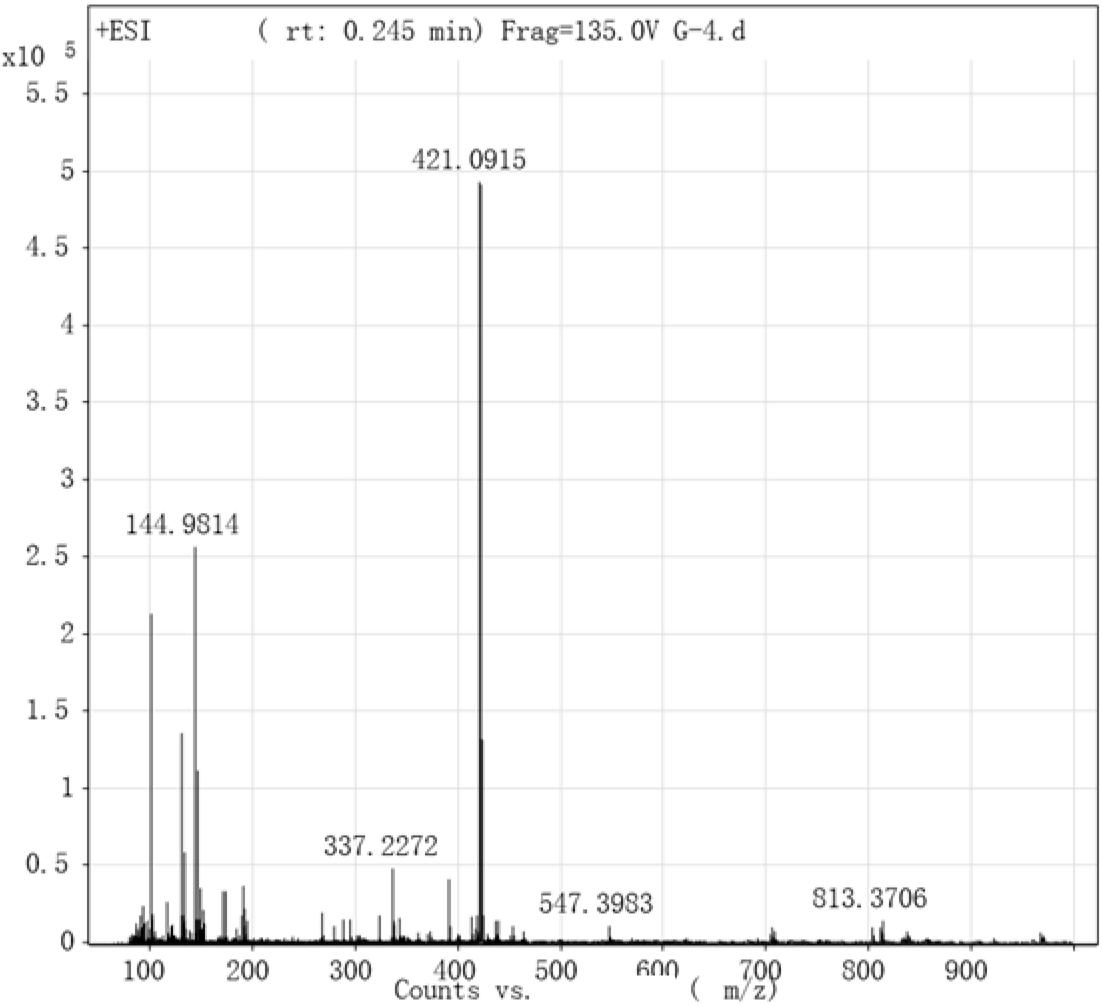

**Figure 73.** $^{1}$H NMR spectrum of **25**

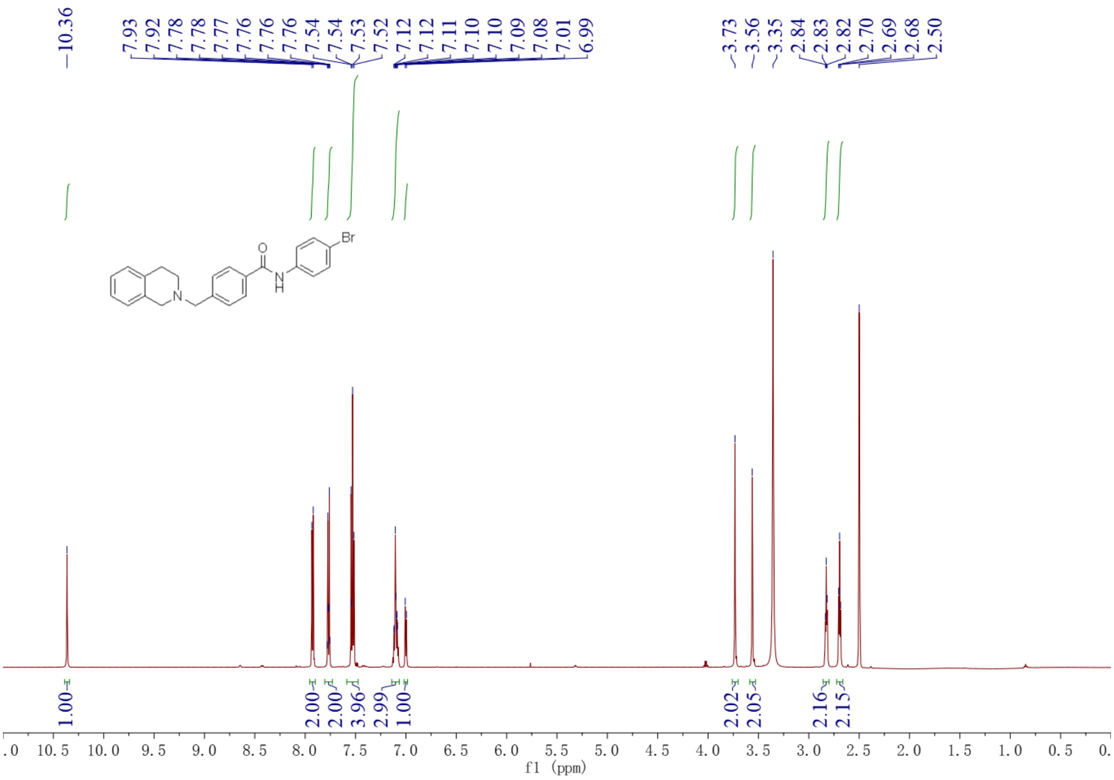


**Figure 74.** $^{13}$C NMR spectrum of **25**

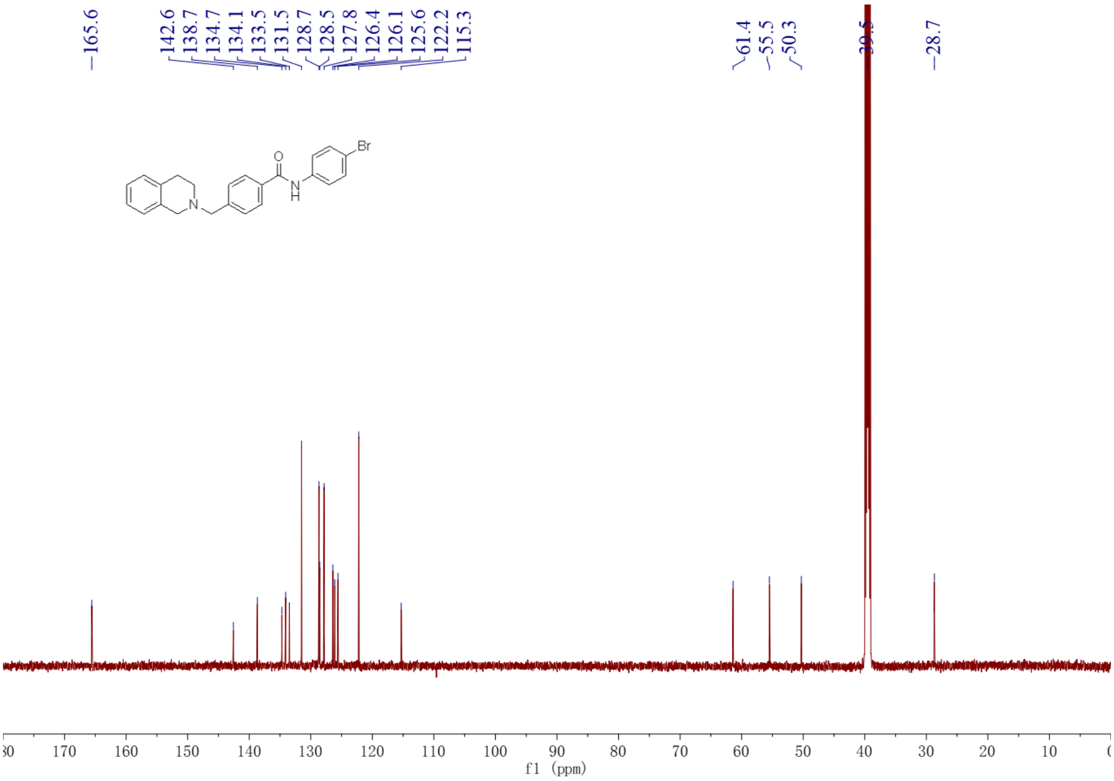

**Figure 75.** HRMS spectrum of **25**

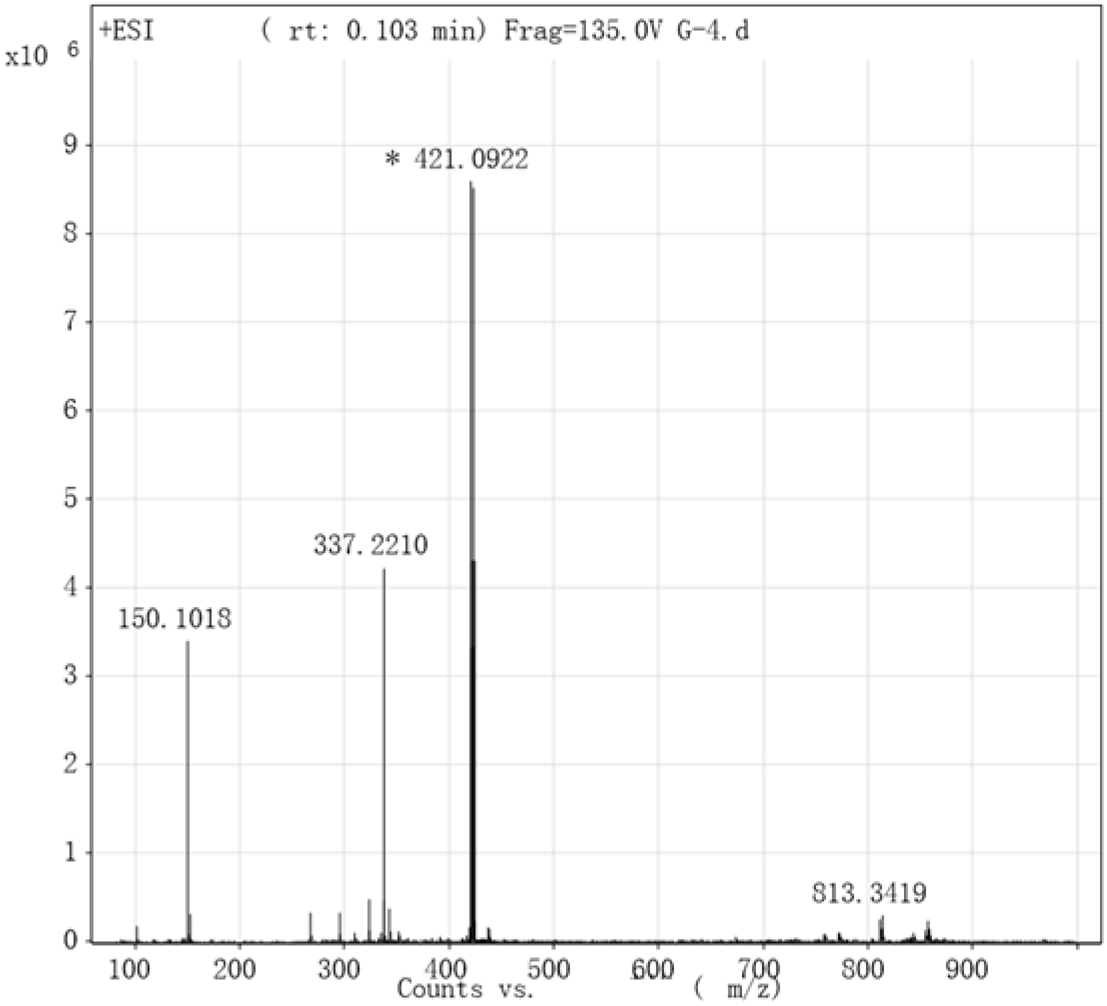

**Figure 76.** $^{1}$H NMR spectrum of **26**

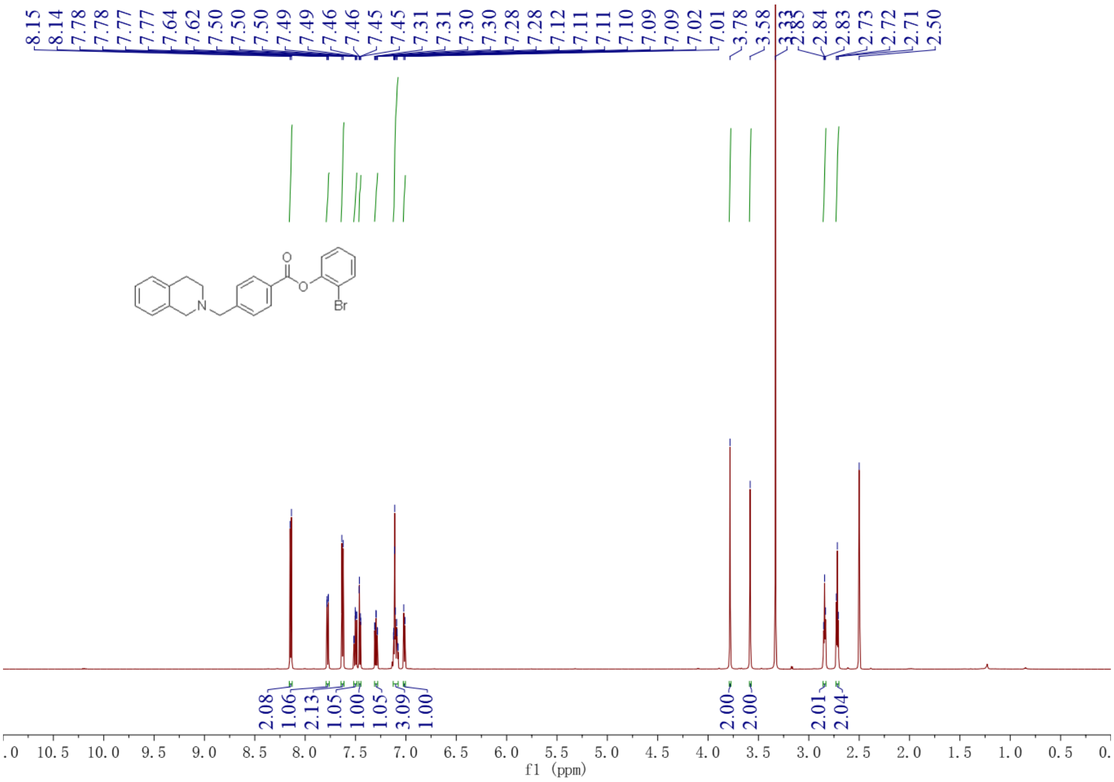

**Figure 77.** $^{13}$C NMR spectrum of **26**

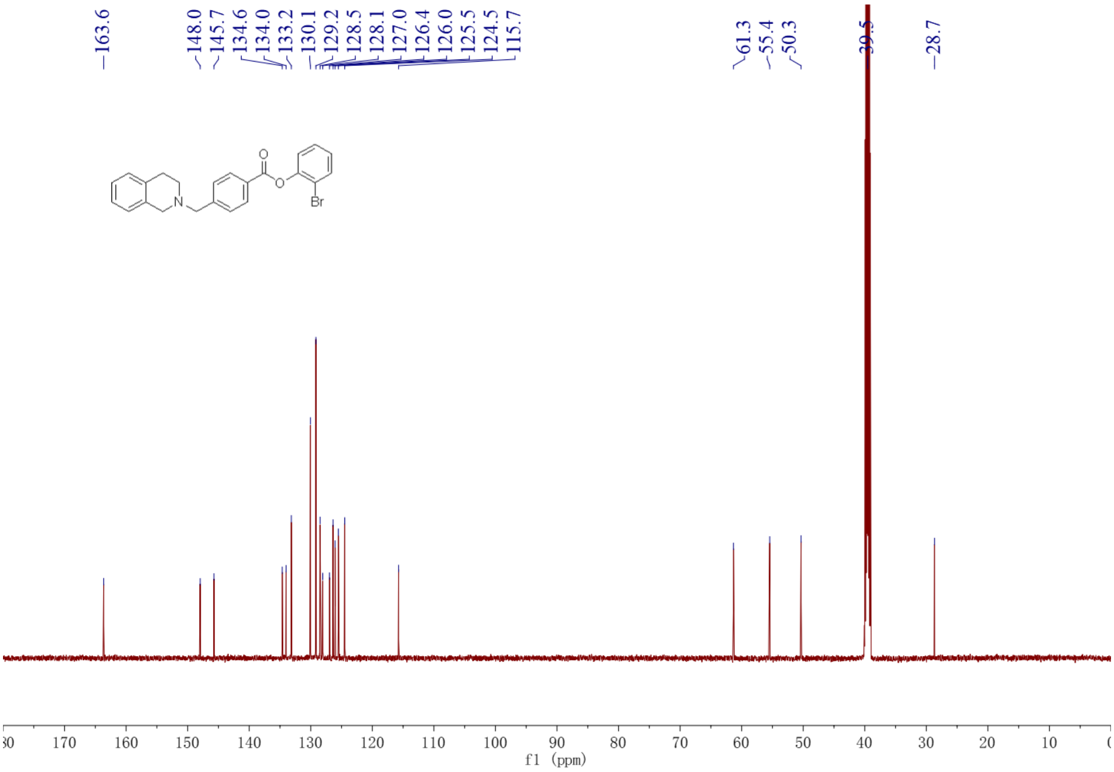

**Figure 78.** HRMS spectrum of **26**

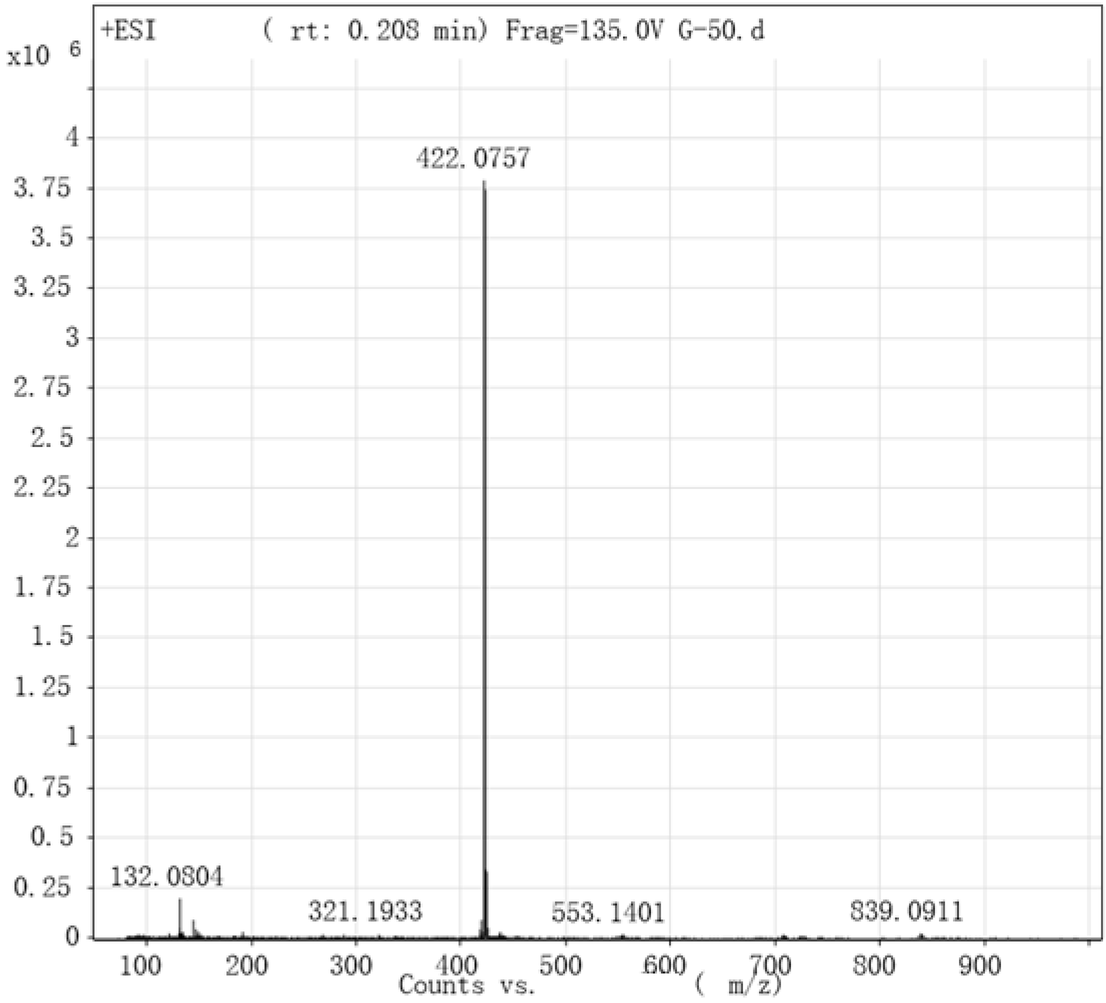

**Figure 79.** $^{1}$H NMR spectrum of **27**

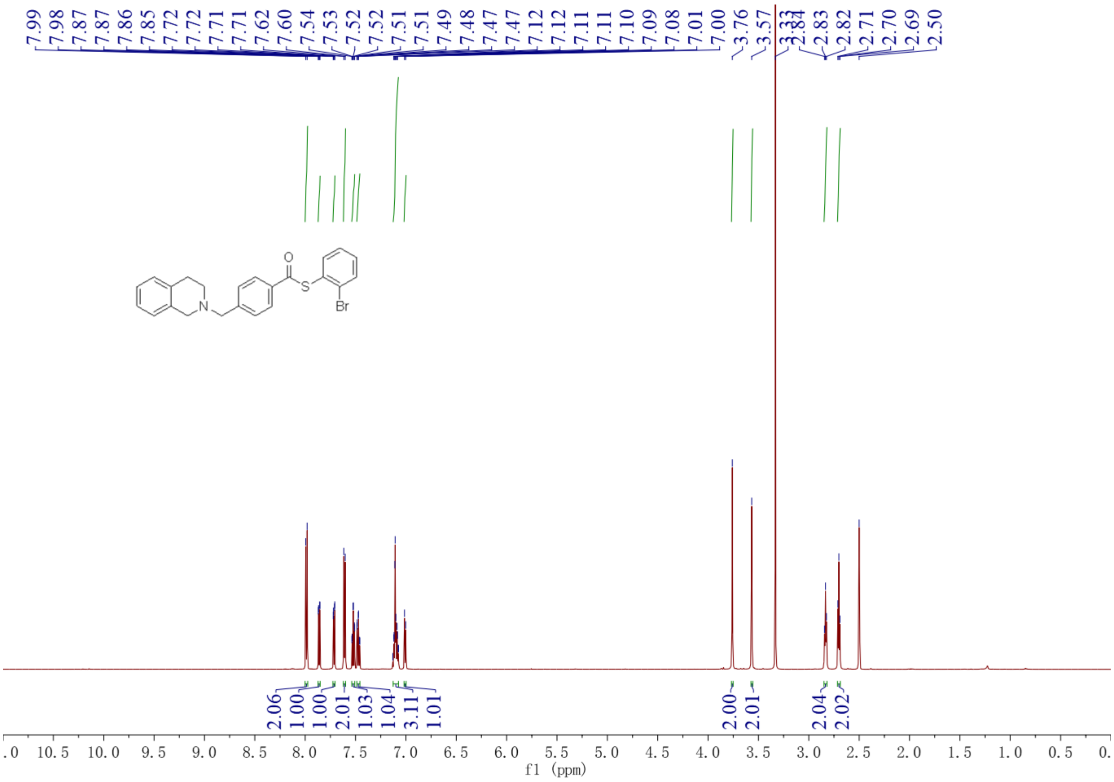


**Figure 80.** $^{13}$C NMR spectrum of **27**

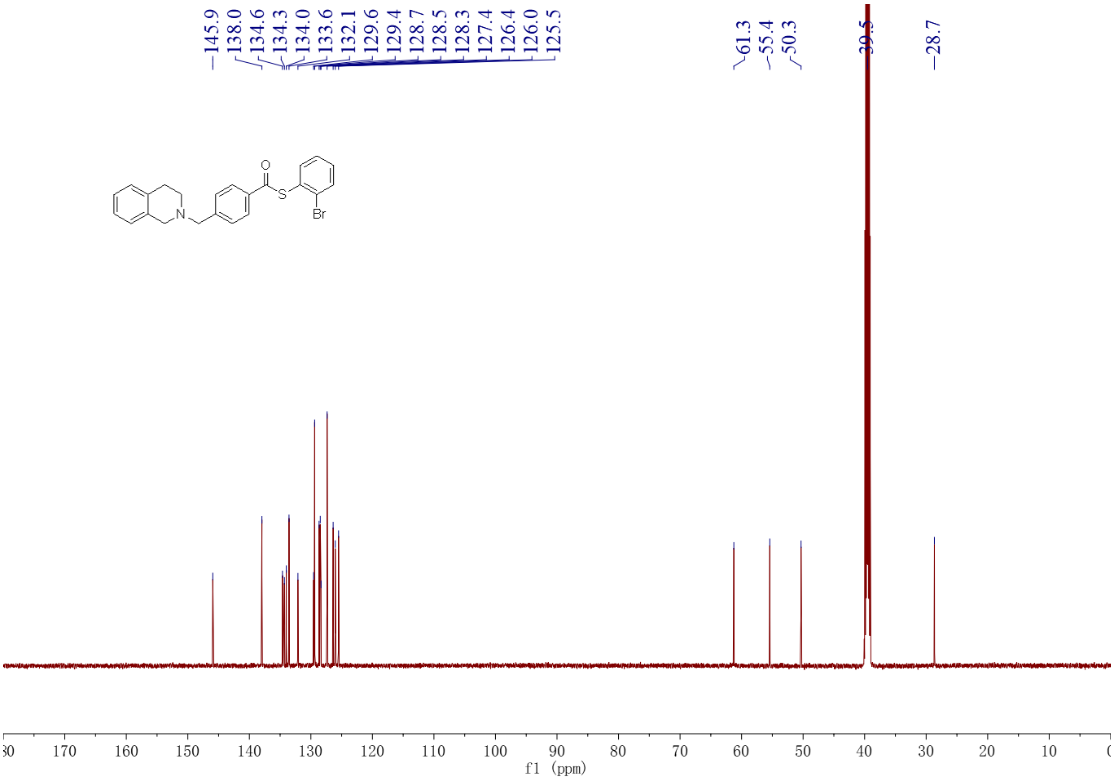

**Figure 81.** HRMS spectrum of **27**

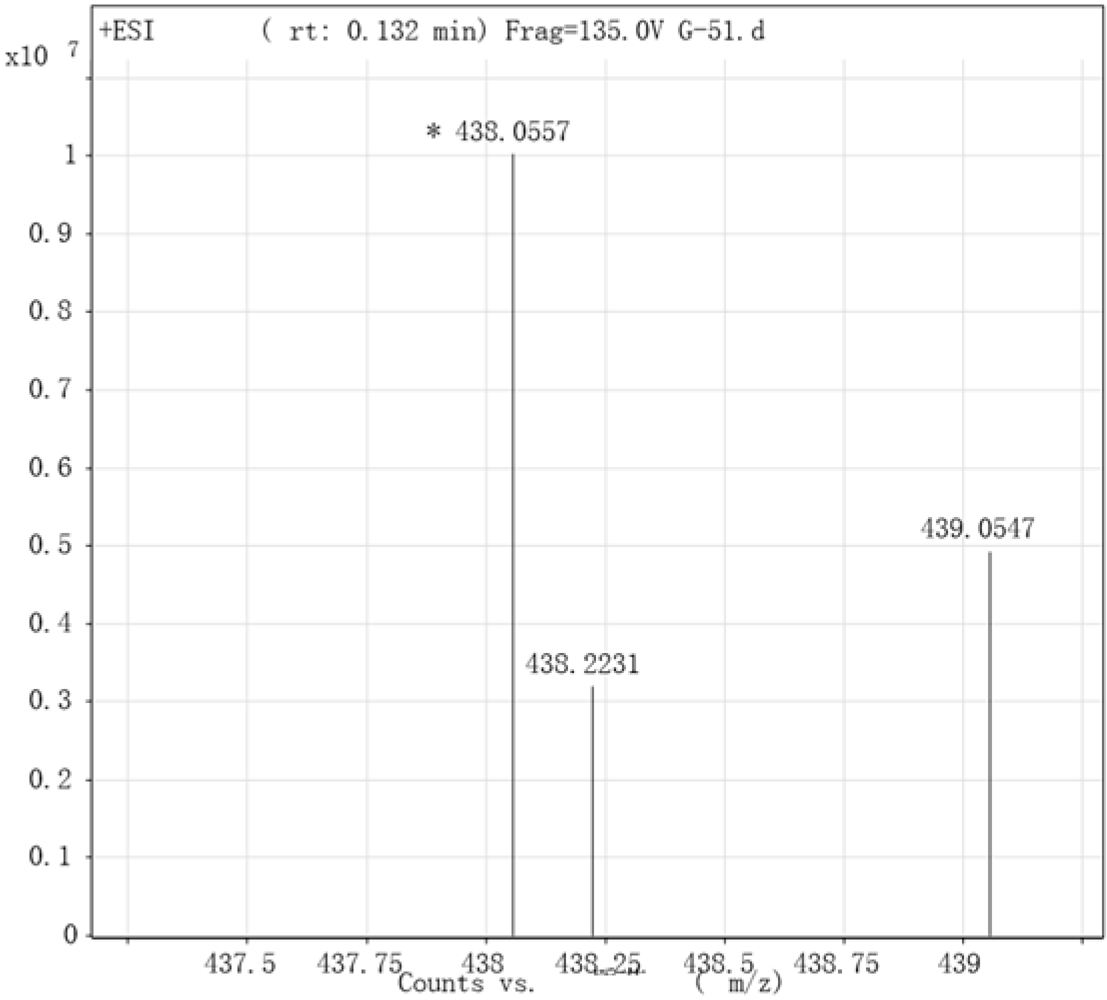

**Figure 82.** $^{1}$H NMR spectrum of **28**

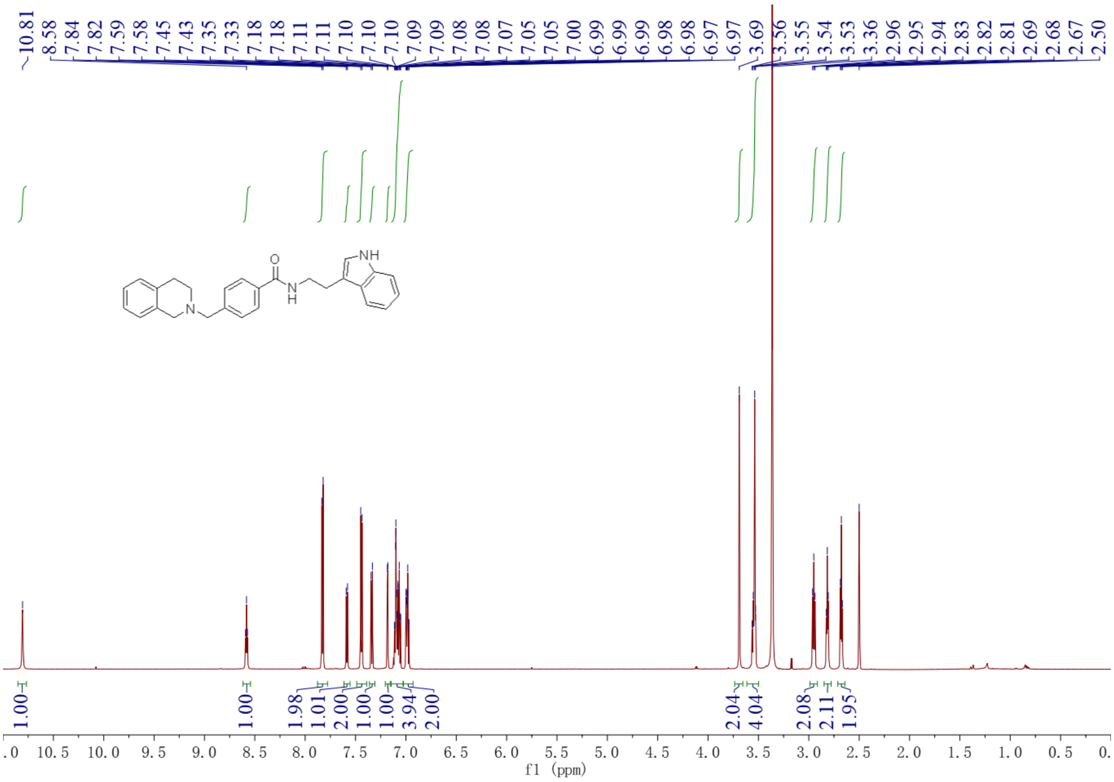

**Figure 83.** $^{13}$C NMR spectrum of **28**

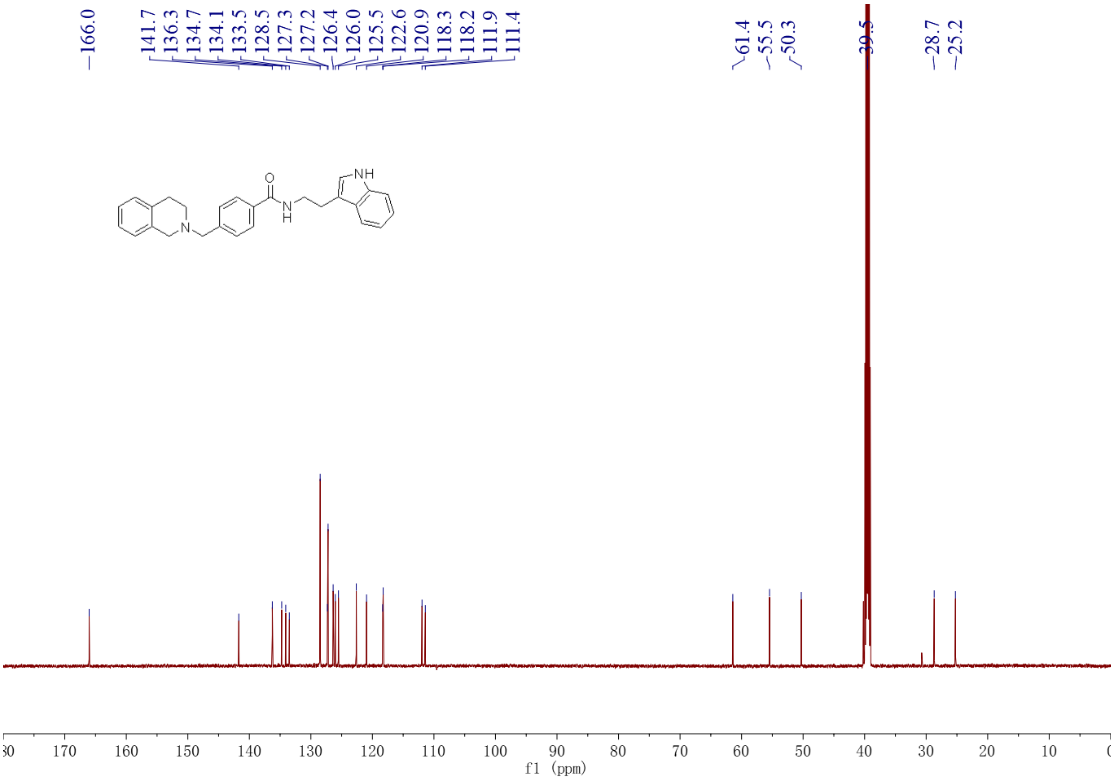

**Figure 84.** HRMS spectrum of **28**

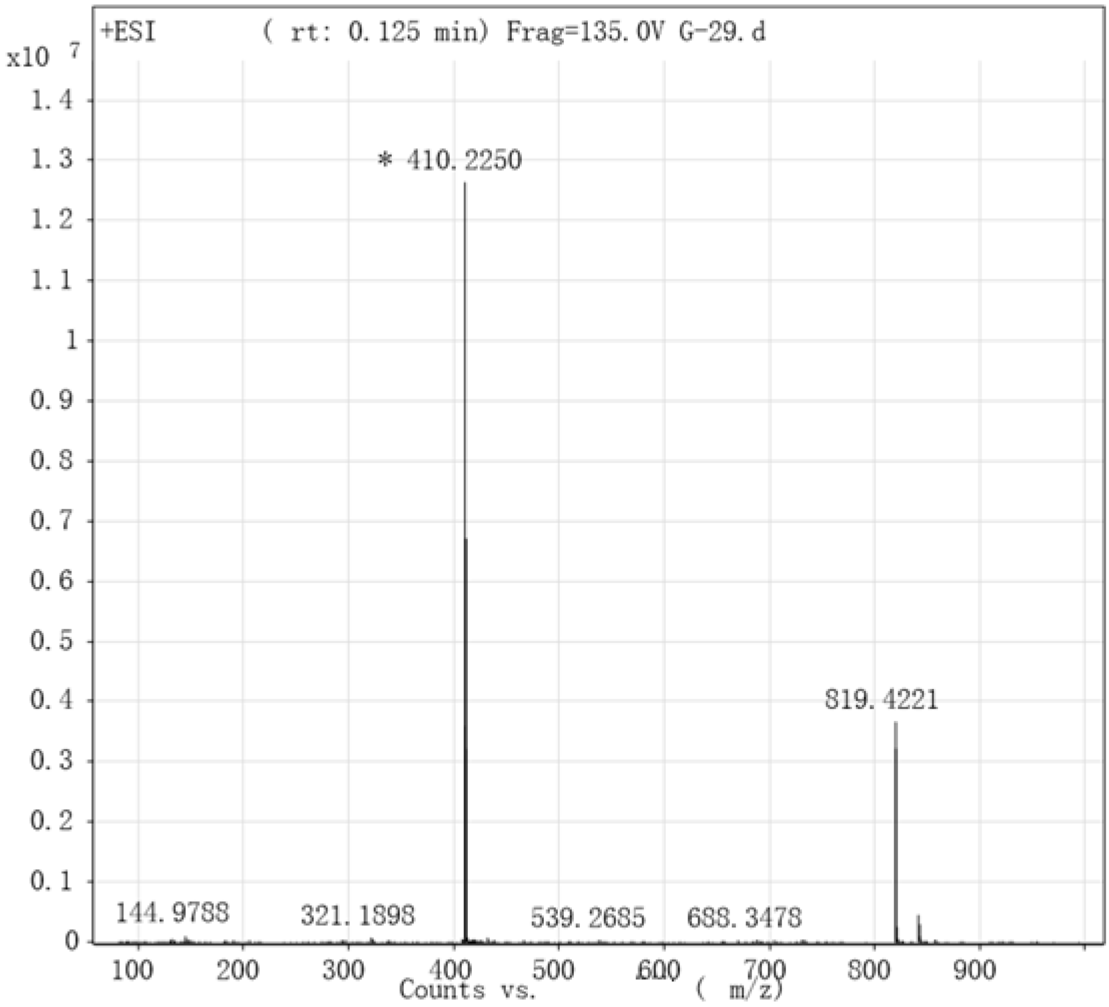

**Figure 85.** $^{1}$H NMR spectrum of **29**

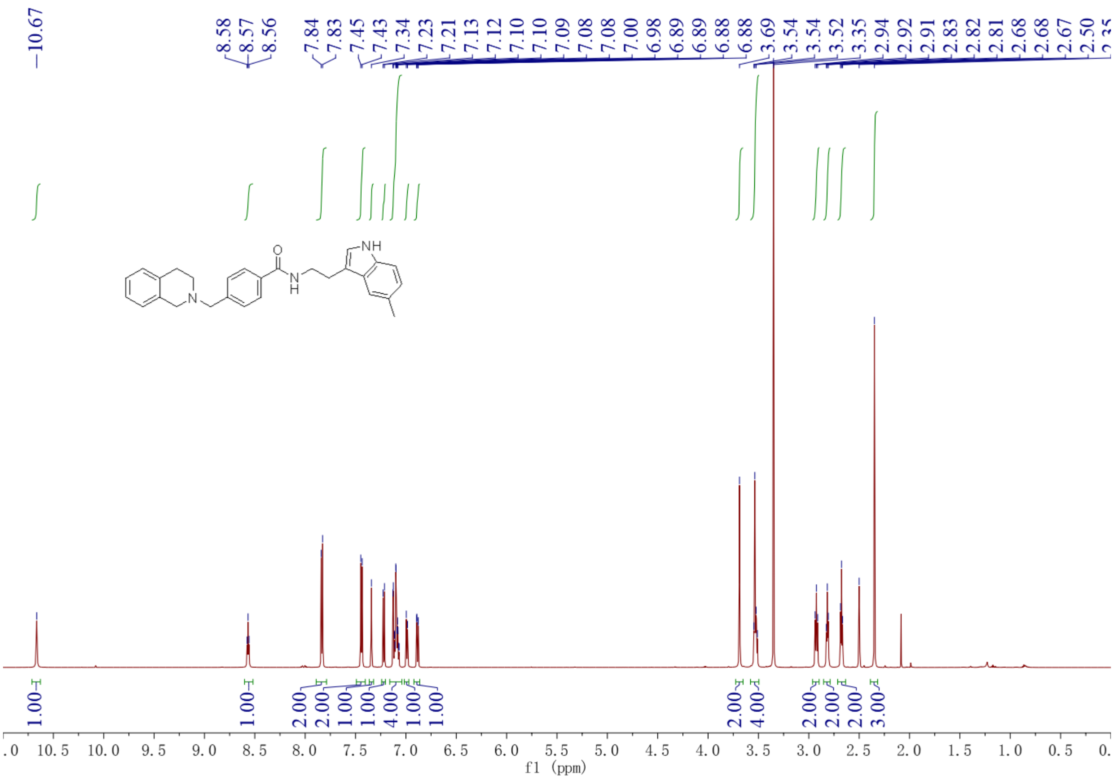

**Figure 86.** $^{13}$C NMR spectrum of **29**

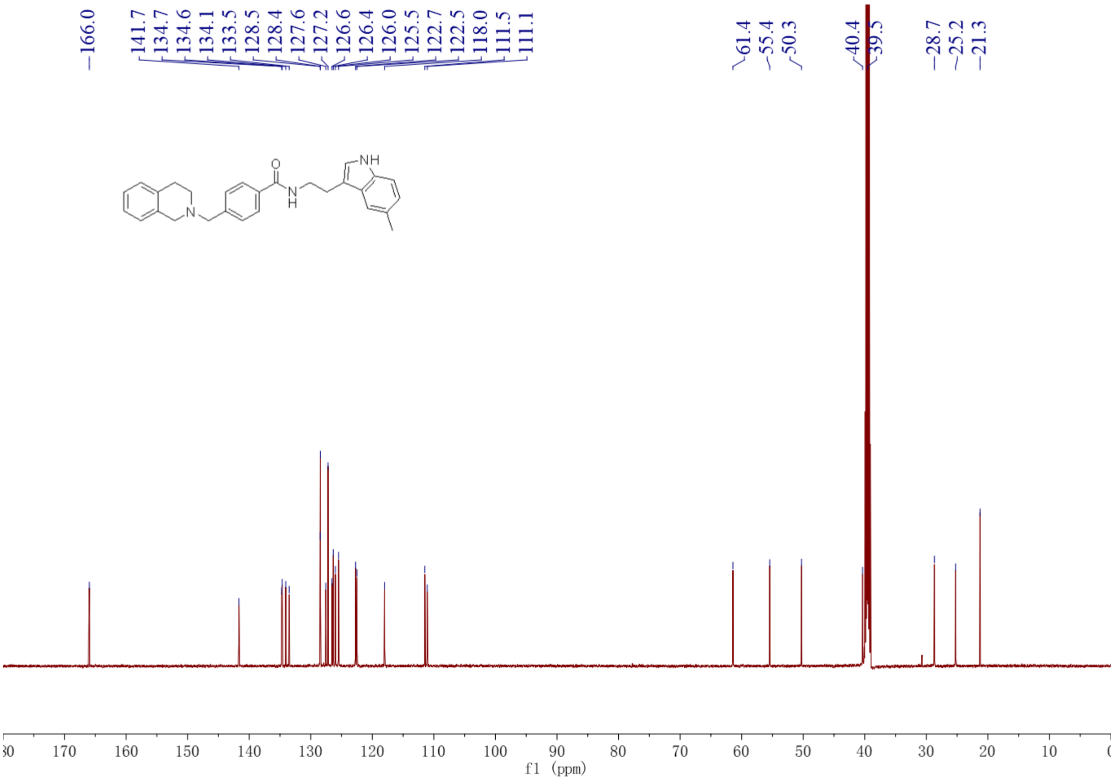

**Figure 87.** HRMS spectrum of **29**

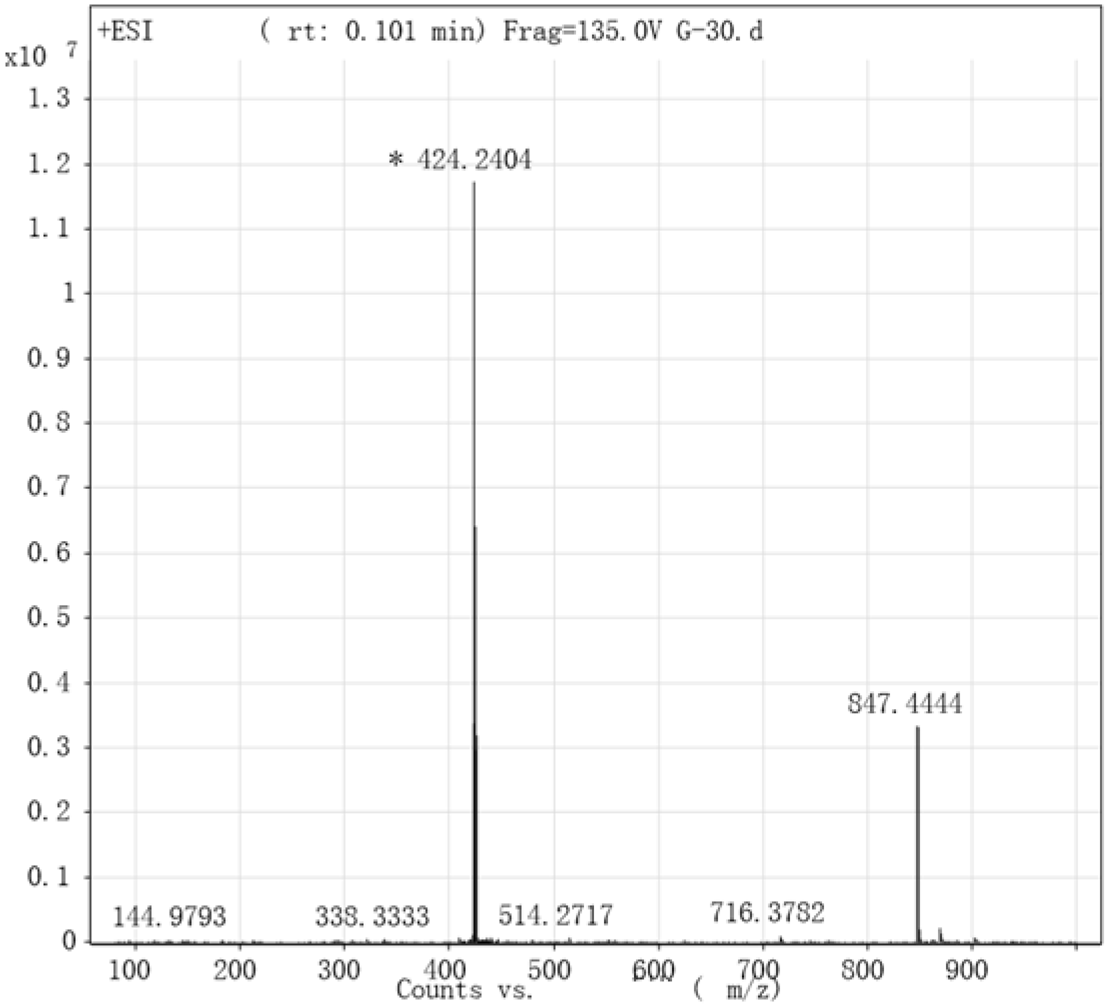

**Figure 88.** $^{1}$H NMR spectrum of **30**

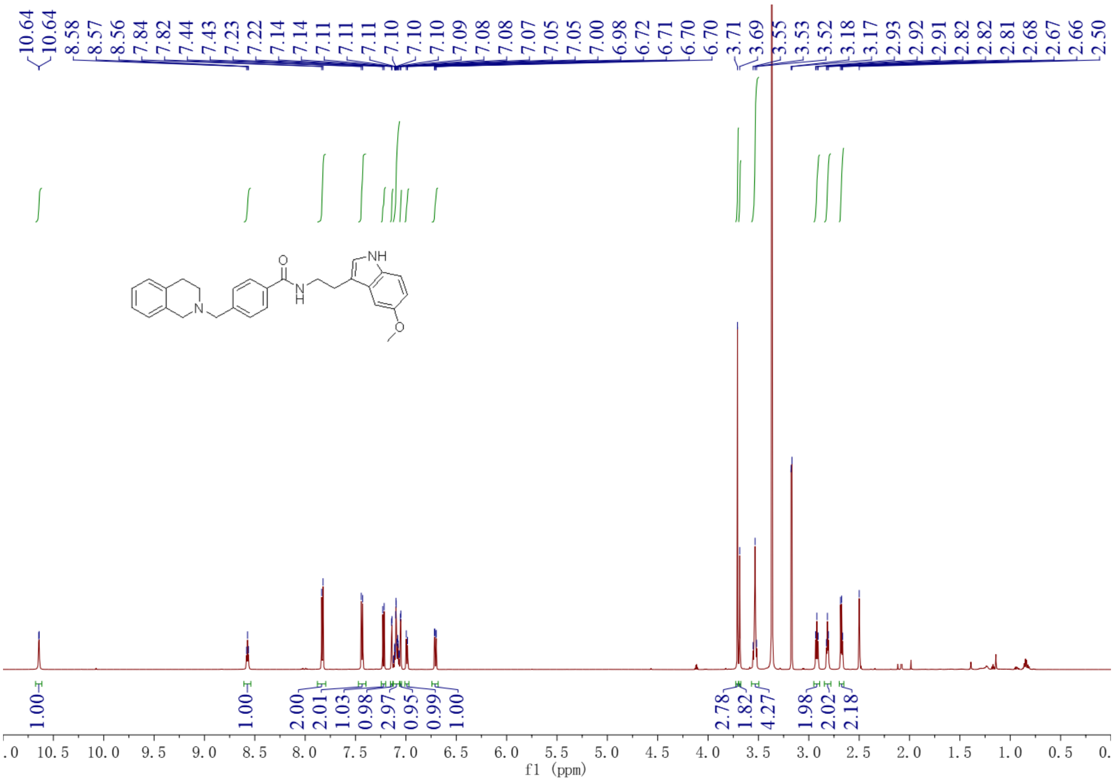

**Figure 89.** $^{13}$C NMR spectrum of **30**

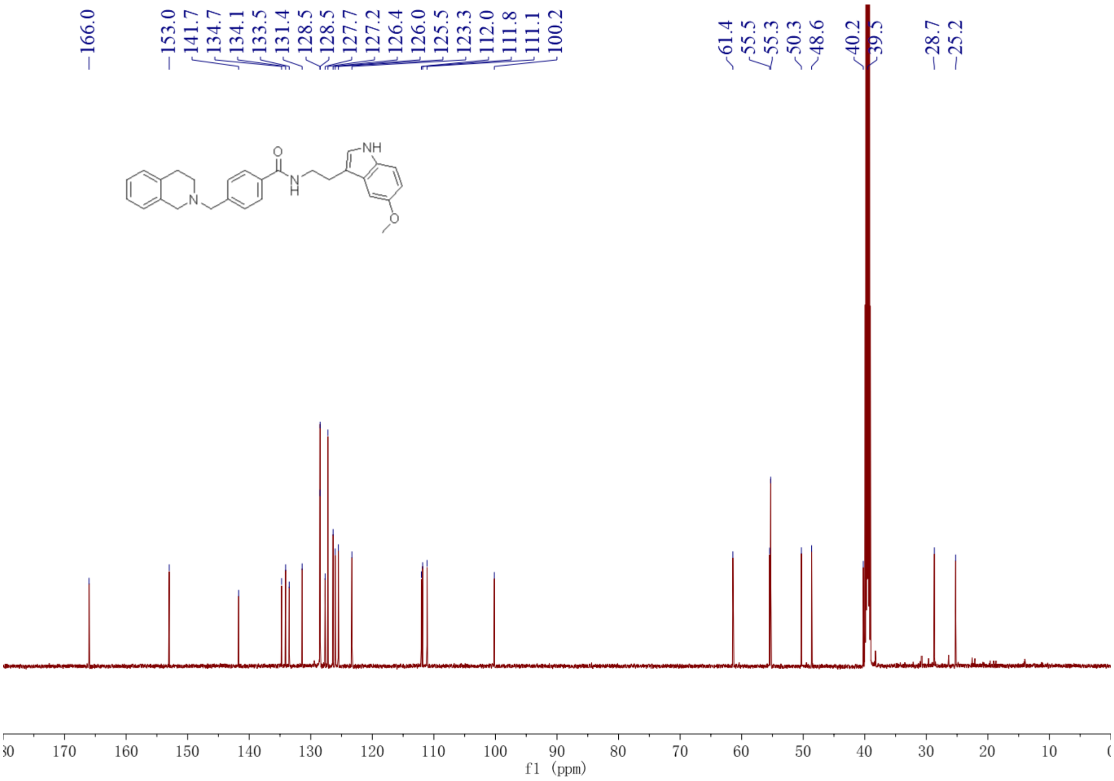

**Figure 90.** HRMS spectrum of **30**

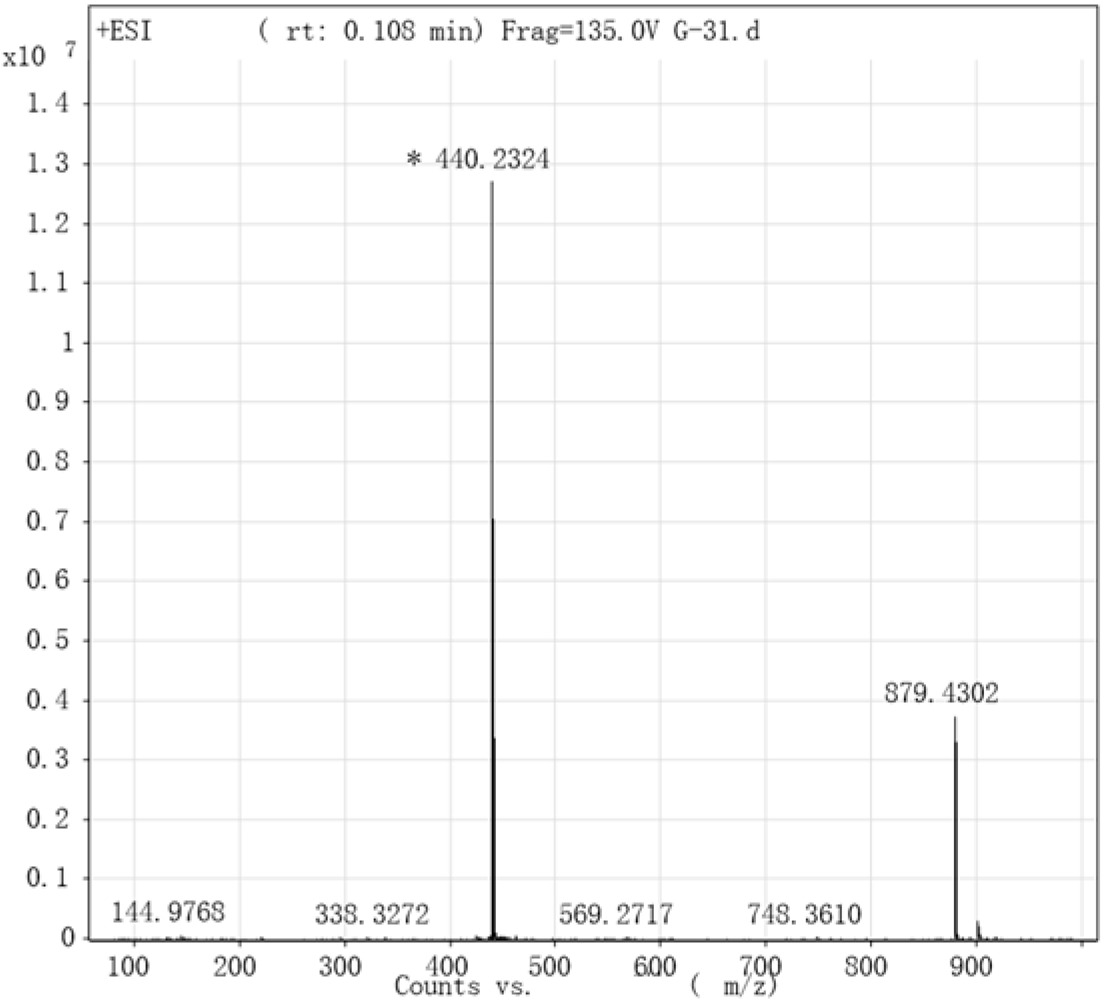

Supplement: Supplementary file 1 [file molecules-24-02568-s001.pdf]
